# Supplementary figures and images for: Genetic Co-Occurrence Network across Sequenced Microbes
Source: PLoS Comput Biol. 2011 Dec 29;7(12):e1002340. doi: 10.1371/journal.pcbi.1002340 (PMC3248385; doi:10.1371/journal.pcbi.1002340)

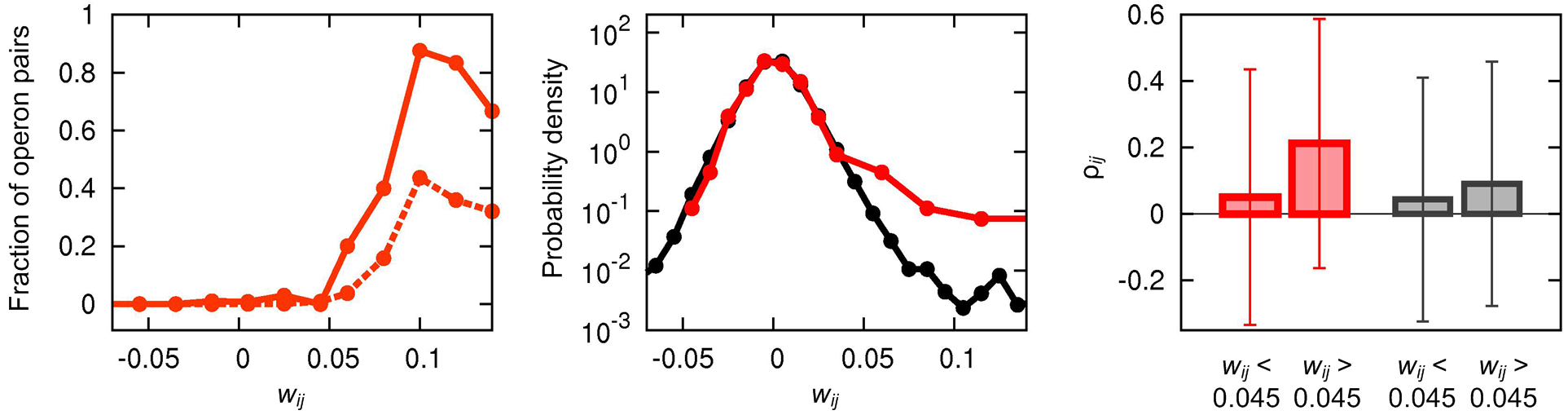

Supplement: Figure S1 — Distinct regimes of protein interactions with different wij's. In the left panel, plotted is the fraction of gene pairs belonging to the same operons among the gene pairs of given wij, which encode physically binding (solid line) or non-binding (dashed line) protein pairs. In the central panel, plotted are the probability density of wij for binding protein pairs (red) and that for arbitrary protein pairs (black), of which both were obtained by excluding the gene pairs belonging to the same operons. Considering only these non-operonic pairs, the right panel shows the averages and standard deviations of the Pearson correlation ρij's of the transcript profiles for every gene pair with wij<0.045 or wij>0.045, among binding protein pairs (red) and among arbitrary protein pairs (black). For more details, refer to Text S1. (TIF) [file pcbi.1002340.s001.tif]

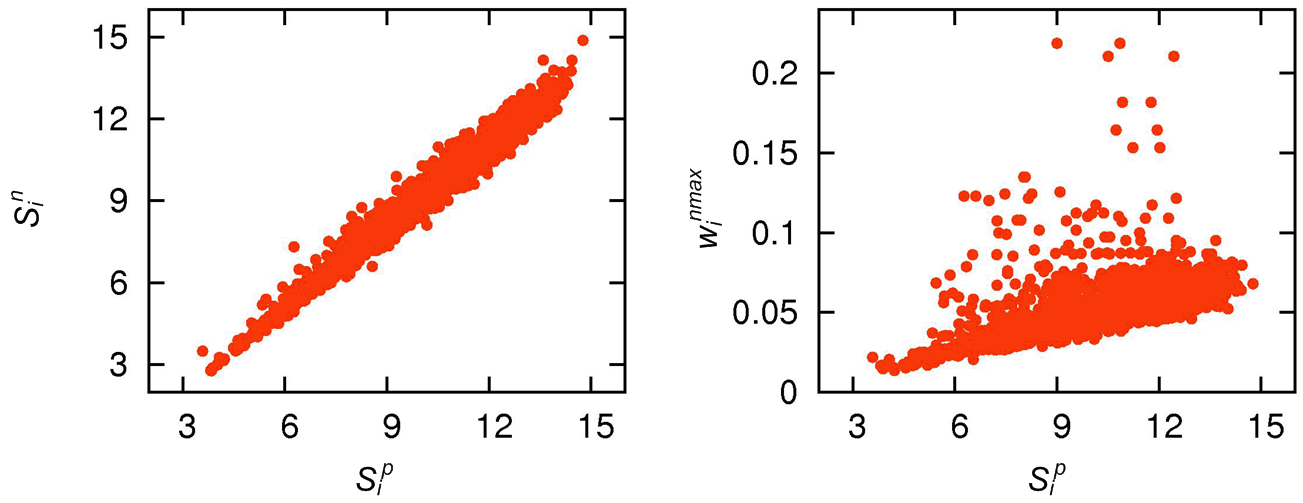

Supplement: Figure S3 — For each gene, scatter plot between Sip and Sin (left panel) and that between Sip and winmax (right panel). For more details, refer to Text S1. (TIF) [file pcbi.1002340.s003.tif]
