# Supplementary material for: Genetic Co-Occurrence Network across Sequenced Microbes
Source: PLoS Comput Biol. 2011 Dec 29;7(12):e1002340. doi: 10.1371/journal.pcbi.1002340 (PMC3248385; doi:10.1371/journal.pcbi.1002340)
Supplement: Table S1 — List of bacterial species used for evaluating correlogy and anti-correlogy between genes. (PDF) [file pcbi.1002340.s004.pdf]

Table S1. List of bacterial species used for evaluating correlogy and anti-correlogy between genes.

**Name**	**Name**
Alicyclobacillus acidocaldarius	*Aquifex aeolicus*
Aggregatibacter aphrophilus	*Candidatus Amoebophilus asiaticus*
Aggregatibacter actinomycetemcomitans	*Arthrobacter aurescens*
Acidovorax avenae	*Acidobacteria bacterium*
Alcanivorax borkumensis	*Arcobacter butzleri*
Acidobacterium capsulatum	*Acinetobacter baumannii ATCC 17978*
Acidothermus cellulolyticus	*Arthrobacter chlorophenolicus*
Acinetobacter sp. ADP1	*Acholeplasma laidlawii*
Acidiphilium cryptum JF-5	*Anaeromyxobacter dehalogenans 2CP-C*
Ammonifex degensii	*Alkalilimnicola ehrlichei*
Acidithiobacillus ferrooxidans ATCC 53993	*Anoxybacillus flavithermus*
Acidimicrobium ferrooxidans DSM 10331	*Anaeromyxobacter sp. Fw109-5*
Aeromonas hydrophila	*Acidovorax sp. JS42*
Anaplasma marginale St. Maries	*Alteromonas macleodii*
Actinosynnema mirum	*Acaryochloris marina*
Alkaliphilus metalliredigens	*Akkermansia muciniphila*
Anabaena sp. PCC7120	*Alkaliphilus oremlandii*
Anaplasma phagocytophilum	*Actinobacillus pleuropneumoniae L20 (serotype 5b)*
Accumulibacter phosphatis	*Anaerococcus prevotii*
Candidatus Azobacteroides pseudotrichonymphae	*Acetobacter pasteurianus*
Atopobium parvulum	*Agrobacterium radiobacter K84*
Arthrobacter sp. FB24	*Aeromonas salmonicida*
Actinobacillus succinogenes	*Anaerocellum thermophilum*
Agrobacterium tumefaciens C58	*Anabaena variabilis*
Agrobacterium vitis S4	*Azotobacter vinelandii*
Phytoplasma AYWB	*Azorhizobium caulinodans*
Azoarcus sp. BH72	*Burkholderia ambifaria MC40-6*
Bifidobacterium adolescentis	*Borrelia afzelii*
Burkholderia cepacia	*Bacillus anthracis Ames*
Bordetella avium	*Bacillus amyloliquefaciens*
Bdellovibrio bacteriovorus	*Brevibacillus brevis*
Bartonella bacilliformis	*Blattabacterium sp. (Blattella germanica)*
Bordetella bronchiseptica	*Borrelia burgdorferi B31*
Bacillus cereus ATCC 14579	*Baumannia cicadellinicola*
Bacillus clausii	*Burkholderia cenocepacia AU1054*
Brucella canis	*Beutenbergia cavernae*
Borrelia duttonii	*Brachybacterium faecium*
Candidatus Blochmannia floridanus	*Bacteroides fragilis YCH46*
Borrelia garinii	*Burkholderia glumae*
Bartonella grahamii	*Bacillus halodurans*
Bartonella henselae	*Borrelia hermsii*
Brachyspira hyodysenteriae	*Beijerinckia indica*
Bradyrhizobium japonicum	*Bifidobacterium animalis subsp. lactis AD011*
Bacillus licheniformis ATCC 14580	*Bifidobacterium longum NCC2705*
Burkholderia mallei ATCC 23344	*Brucella abortus 9-941*
Brucella melitensis 16M	*Brucella microti*
Brucella suis 1330	*Burkholderia multivorans ATCC 17616 (JGI)*
Brucella ovis	*Bordetella parapertussis*
Bordetella pertussis	*Burkholderia phymatum*
Candidatus Blochmannia pennsylvanicus	*Burkholderia pseudomallei K96243*
Bordetella petrii	*Bacillus pumilus*
Burkholderia phytofirmans	*Bartonella quintana*
Bradyrhizobium sp. ORS278	*Borrelia recurrentis*
Bacillus subtilis	*Burkholderia thailandensis*
Bacteroides thetaiotaomicron	*Bacillus thuringiensis 97-27*
Bartonella tribocorum	*Borrelia turicatae*
Buchnera aphidicola APS	*Burkholderia sp. 383*
Burkholderia vietnamiensis	*Bacteroides vulgatus*
Bacillus weihenstephanensis	*Burkholderia xenovorans*
Chlamydophila abortus	*Clostridium acetobutylicum*

Name	Name
Chloroflexus aggregans	*Catenulispora acidiphila*
Caulobacter sp. K31	*Corynebacterium aurimucosum*
Chloroflexus aurantiacus	*Clostridium beijerinckii*
Clostridium botulinum A ATCC 3502	*Coxiella burnetii RSA 493*
Chlamydophila caviae	*Clostridium cellulolyticum*
Chlorobium chlorochromatii	*Campylobacter concisus 13826*
Caulobacter crescentus CB15	*Cryptobacterium curtum*
Campylobacter curvus	*Clostridium difficile 630*
Corynebacterium diphtheriae	*Corynebacterium efficiens*
Chlamydophila felis	*Campylobacter fetus*
Corynebacterium glutamicum ATCC 13032 (Kyowa Hakko)	*Campylobacter hominis ATCC BAA-381*
Chloroflexus sp. Y-400-fl	*Cytophaga hutchinsonii*
Carboxydothermus hydrogenoformans	*Cellvibrio japonicus*
Campylobacter jejuni NCTC11168	*Corynebacterium jeikeium*
Clostridium kluyveri DSM 555	*Citrobacter koseri ATCC BAA-895*
Corynebacterium kroppenstedtii	*Campylobacter lari*
Chlorobium limicola	*Clavibacter michiganensis subsp. michiganensis*
Chlamydia muridarum	*Clostridium novyi*
Capnocytophaga ochracea	*Chlorobaculum parvum NCIB 8327*
Clostridium perfringens 13	*Chlorobium phaeobacteroides DSM 266*
Chitinophaga pinensis	*Chlamydophila pneumoniae CWL029*
Coprothermobacter proteolyticus	*Colwellia psychrerythraea*
Clostridium phytofermentans	*Chromohalobacter salexigens*
Caldicellulosiruptor saccharolyticus	*Clostridium tetani E88*
Chlorobaculum tepidum	*Clostridium thermocellum*
Cupriavidus taiwanensis	*Chlamydia trachomatis D/UW-3/CX (serovar D)*
Chloroherpeton thalassium	*Cronobacter turicensis*
Corynebacterium urealyticum	*Chromobacterium violaceum*
Cyanobacteria Yellowstone A-Prime	*Cyanothece sp. ATCC 51142*
Delftia acidovorans	*Desulfotomaculum acetoxidans*
Desulfatibacillum alkenivorans	*Dechloromonas aromatica*
Desulfobacterium autotrophicum	*Candidatus Desulforudis audaxviator*
Desulfomicrobium baculatum	*Dickeya dadantii*
Desulfovibrio desulfuricans G20	*Deinococcus deserti*
Dehalococcoides sp. CBDB1	*Dehalococcoides ethenogenes*
Dyadobacter fermentans	*Deinococcus geothermalis*
Diaphorobacter sp. TPSY	*Desulfovibrio magneticus*
Dichelobacter nodosus	*Desulfococcus oleovorans*
Desulfotalea psychrophila	*Deinococcus radiodurans*
Desulfotomaculum reducens	*Desulfohalobium retbaense*
Desulfovibrio salexigens	*Dinoroseobacter shibae*
Desulfitobacterium hafniense Y51	*Dictyoglomus thermophilum*
Dictyoglomus turgidum	*Desulfovibrio vulgaris Hildenborough*
Dickeya zeae	*Exiguobacterium sp. AT1b*
Aromatoleum aromaticum EbN1	*Erwinia carotovora*
Ehrlichia chaffeensis	*Ehrlichia canis*
Escherichia coli K-12 MG1655	*Eubacterium eligens*
Enterococcus faecalis	*Escherichia fergusonii*
Edwardsiella ictaluri	*Eggerthella lenta*
Erythrobacter litoralis	*Elusimicrobium minutum*
Enterobacter sp. 638	*Eubacterium rectale*
Ehrlichia ruminantium Welgevonden (South Africa)	*Enterobacter sakazakii*
Exiguobacterium sibiricum	*Erwinia tasmaniensis*
Frankia alni	*Flavobacteriaceae bacterium*
Flavobacterium johnsoniae	*Finegoldia magna*
Fervidobacterium nodosum	*Fusobacterium nucleatum*
Francisella philomiragia	*Flavobacterium psychrophilum*
Frankia sp. CcI3	*Francisella tularensis subsp. tularensis SCHU S4*
Gemmatimonas aurantiaca	*Granulobacter bethesdensis*
Geobacter bemidjiensis	*Gluconacetobacter diazotrophicus PAl 5 (Brazil)*
Geobacter sp. FRC-32	*Gramella forsetii*
Geobacillus kaustophilus	*Geobacter lovleyi*

Name	Name
Geobacter metallireducens	*Gluconobacter oxydans*
Geobacter sulfurreducens	*Geobacillus thermodenitrificans*
Geobacter uraniumreducens	*Gloeobacter violaceus*
Geobacillus sp. WCH70	*Helicobacter acinonychis*
Haemophilus parasuis	*Herminiimonas arsenicoxydans*
Herpetosiphon aurantiacus	*Hirschia baltica*
Hahella chejuensis	*Candidatus Hamiltonella defensa*
Haemophilus ducreyi	*Halorhodospira halophila*
Helicobacter hepaticus	*Haemophilus influenzae Rd KW20 (serotype d)*
Heliobacterium modesticaldum	*Halothiobacillus neapolitanus*
Hyphomonas neptunium	*Haliangium ochraceum*
Halothermothrix orenii	*Helicobacter pylori 26695*
Haemophilus somnus 129PT	*Hydrogenobaculum sp. Y04AAS1*
Idiomarina loihiensis	*Jannaschia sp. CCS1*
Jonesia denitrificans	*Kangiella koreensis*
Kosmotoga olearia	*Klebsiella pneumoniae*
Kineococcus radiotolerans	*Kocuria rhizophila*
Kytococcus sedentarius	*Lactobacillus acidophilus*
Candidatus Liberibacter asiaticus	*Leptotrichia buccalis*
Leptospira biflexa serovar Patoc Patoc 1 (Paris)	*Leptospira borgpetersenii JB197*
Lactobacillus brevis	*Lactobacillus casei ATCC 334*
Leptothrix cholodnii	*Leuconostoc citreum*
Lactobacillus delbrueckii ATCC 11842	*Lactobacillus fermentum*
Lactobacillus gasseri	*Lactobacillus helveticus*
Laribacter hongkongensis	*Leptospira interrogans serovar lai*
Listeria innocua	*Lawsonia intracellularis*
Lactobacillus johnsonii NCC 533	*Lactococcus lactis subsp. lactis IL1403*
Leuconostoc mesenteroides	*Listeria monocytogenes EGD-e*
Lactobacillus plantarum WCFS1	*Legionella pneumophila Philadelphia 1*
Lactobacillus reuteri DSM 20016	*Lactobacillus rhamnosus*
Lactobacillus sakei	*Lactobacillus salivarius*
Lysinibacillus sphaericus	*Listeria welshimeri SLCC5334*
Leifsonia xyli xyli CTCB07	*Mycoplasma agalactiae*
Mycobacterium abscessus ATCC 19977T	*Magnetospirillum magneticum*
Marinobacter aquaeolei	*Microcystis aeruginosa*
Mycoplasma arthritidis	*Mycobacterium bovis AF2122/97*
Methylococcus capsulatus	*Methylobacterium chloromethanicum*
Macrococcus caseolyticus	*Mycoplasma conjunctivae*
Mycoplasma capricolum	*Methylobacterium extorquens DM4*
Methylovorus sp. SIP3-4	*Mesorhizobium sp. BNC1*
Methylobacterium sp. 4-46	*Methylobacterium extorquens*
Methylobacillus flagellatus	*Mesoplasma florum*
Mycoplasma gallisepticum	*Mycoplasma genitalium*
Mycobacterium gilvum	*Magnetococcus sp. MC-1*
Mycoplasma hyopneumoniae 232	*Methylacidiphilum infernorum*
Mycobacterium leprae TN	*Mesorhizobium loti*
Micrococcus luteus	*Methylotenera mobilis*
Mycobacterium sp. MCS	*Mycobacterium marinum M*
Mycoplasma mobile	*Maricaulis maris*
Minibacterium massiliensis	*Marinomonas sp. MWYL1*
Mycoplasma mycoides	*Methylobacterium nodulans*
Mycobacterium avium paratuberculosis	*Mycoplasma penetrans*
Mycoplasma pneumoniae	*Methylobacterium populi*
Methylibium petroleiphilum	*Mycoplasma pulmonis*
Methylobacterium radiotolerans	*Methylocella silvestris*
Mycobacterium smegmatis	*Mannheimia succiniciproducens*
Mycoplasma synoviae	*Moorella thermoacetica*
Mycobacterium tuberculosis H37Rv	*Mycobacterium ulcerans*
Mycobacterium vanbaalenii	*Myxococcus xanthus*
Nautilia profundicola	*Novosphingobium aromaticivorans*
Nocardioides sp. JS614	*Nitrosomonas eutropha*
Nitrosomonas europaea	*Nocardia farcinica*

Name	Name
Neisseria gonorrhoeae FA 1090	*Nitrobacter hamburgensis*
Nitratiruptor sp. SB155-2	*Neisseria meningitidis MC58 (serogroup B)*
Nakamurella multipartita	*Nitrosospira multiformis*
Nitrosococcus oceani	*Nostoc punctiforme*
Neorickettsia risticii	*Neorickettsia sennetsu*
Natranaerobius thermophilus	*Nitrobacter winogradskyi*
Ochrobactrum anthropi	*Oligotropha carboxidovorans*
Oceanobacillus iheyensis	*Oenococcus oeni*
Opitutus terrae	*Orientia tsutsugamushi Boryong*
Prosthecochloris aestuarii	*Propionibacterium acnes*
Pseudomonas aeruginosa PAO1	*Candidatus Phytoplasma australiense*
Psychrobacter arcticum	*Pseudoalteromonas atlantica*
Photorhabdus asymbiotica	*Pelobacter carbinolicus*
Psychrobacter cryohalolentis	*Pectobacterium carotovorum*
Candidatus Protochlamydia amoebophila	*Paracoccus denitrificans*
Parabacteroides distasonis	*Pseudomonas entomophila*
Pseudomonas fluorescens Pf-5	*Porphyromonas gingivalis W83*
Pseudoalteromonas haloplanktis	*Pedobacter heparinus*
Psychromonas ingrahamii	*Paenibacillus sp. JDR-2*
Parvibaculum lavamentivorans	*Pelodictyon luteolum*
Photorhabdus luminescens	*Prochlorococcus marinus SS120*
Candidatus Phytoplasma mali	*Petrotoga mobilis*
Proteus mirabilis	*Pasteurella multocida*
Persephonella marina	*Pseudomonas mendocina*
Polaromonas naphthalenivorans	*Polynucleobacter necessarius*
Polynucleobacter sp. QLW-P1DMWA-1	*Polaromonas sp. JS666*
Phytoplasma OY	*Pelobacter propionicus*
Pediococcus pentosaceus	*Pelodictyon phaeoclathratiforme*
Photobacterium profundum	*Pseudomonas putida KT2440*
Psychrobacter sp. PRwf-1	*Pseudomonas stutzeri*
Pseudomonas syringae pv. tomato DC3000	*Pelotomaculum thermopropionicum*
Candidatus Pelagibacter ubique	*Chlorobium vibrioformis*
Phenylobacterium zucineum	*Rickettsia africae*
Rickettsia akari	*Rhodopirellula baltica*
Rickettsia bellii RML369-C	*Robiginitalea biformata*
Roseiflexus castenholzii DSM13941	*Rhodospirillum centenum*
Rickettsia canadensis	*Rickettsia conorii*
Roseobacter denitrificans	*Rhodococcus erythropolis*
Rhizobium etli CFN 42	*Ralstonia eutropha JMP134*
Rickettsia felis	*Rhodoferax ferrireducens*
Rhodococcus sp. RHA1	*Rhizobium sp. NGR234*
Rhizobium leguminosarum	*Candidatus Ruthia magnifica*
Ralstonia metallidurans	*Rickettsia massiliae*
Rhodococcus opacus	*Rhodopseudomonas palustris CGA009*
Ralstonia pickettii 12J	*Rickettsia peacockii*
Rickettsia prowazekii	*Rickettsia rickettsii Sheila Smith*
Roseiflexus sp. RS-1	*Rhodospirillum rubrum*
Renibacterium salmoninarum	*Ralstonia solanacearum*
Rhodobacter sphaeroides 2.4.1	*Rickettsia typhi*
Rubrobacter xylanophilus	*Sulfurihydrogenibium azorense*
Streptococcus agalactiae 2603 (serotype V)	*Sphingopyxis alaskensis*
Salinispora arenicola	*Syntrophus aciditrophicus*
Staphylococcus aureus N315	*Shewanella amazonensis*
Shewanella baltica OS155	*Shigella boydii Sb227*
Staphylococcus carnosus	*Sorangium cellulosum*
Streptomyces coelicolor	*Saccharophagus degradans*
Shewanella denitrificans	*Streptococcus dysgalactiae*
Shigella dysenteriae	*Saccharopolyspora erythraea*
Staphylococcus epidermidis ATCC 12228	*Streptococcus equi subsp. zooepidemicus MGCS10565*
Shigella flexneri 301 (serotype 2a)	*Shewanella frigidimarina*
Syntrophobacter fumaroxidans	*Sodalis glossinidius*
Streptococcus gordonii	*Streptomyces griseus*

Name	Name
Staphylococcus haemolyticus	*Shewanella sp. MR-4*
Slackia heliotrinireducens	*Shewanella halifaxensis*
Silicibacter pomeroyi	*Silicibacter sp. TM1040*
Shewanella loihica	*Streptomyces avermitilis*
Sinorhizobium medicae	*Sinorhizobium meliloti*
Stenotrophomonas maltophilia K279a	*Streptococcus mutans*
Shewanella oneidensis	*Shewanella putrefaciens*
Serratia proteamaculans	*Shewanella pealeana*
Streptococcus pneumoniae TIGR4 (virulent serotype 4)	*Streptococcus pyogenes SF370 (serotype M1)*
Salinibacter ruber	*Streptococcus sanguinis*
Shewanella sediminis	*Shigella sonnei*
Staphylococcus saprophyticus	*Streptococcus suis 05ZYH33*
Streptococcus thermophilus CNRZ1066	*Symbiobacterium thermophilum*
Salmonella typhimurium LT2	*Salinispora tropica*
Salmonella enterica subsp. enterica serovar Typhi CT18	*Streptococcus uberis*
Sulfurihydrogenibium sp. YO3AOP1	*Sulfurovum sp. NBC37-1*
Solibacter usitatus	*Saccharomonospora viridis*
Shewanella woodyi ATCC 51908	*Sphingomonas wittichii*
Syntrophomonas wolfei	*Shewanella piezotolerans WP3*
Synechococcus elongatus PCC6301	*Synechocystis sp. PCC6803*
Synechococcus sp. WH8102	*Thermosipho africanus*
Tolumonas auensis	*Thiobacillus denitrificans*
Thiomicrospira crunogena	*Treponema denticola*
Sulfurimonas denitrificans	*Thermosynechococcus elongatus*
Trichodesmium erythraeum	*Thermoanaerobacter sp. X514*
Thermobifida fusca	*Thioalkalivibrio sp. HL-EbGR7*
Thermotoga lettingae	*Thermotoga maritima*
Thermosipho melanesiensis	*Thauera sp. MZ1T*
Thermotoga neapolitana	*Treponema pallidum subsp. pallidum Nichols*
Thermoanaerobacter pseudethanolicus	*Thermotoga petrophila*
Thermomicrobium roseum	*Thermotoga sp. RQ2*
Thermoanaerobacter tengcongensis	*Thermus thermophilus HB27*
Teredinibacter turnerae	*Tropheryma whipplei Twist*
Thermodesulfovibrio yellowstonii	*Ureaplasma parvum serovar 3 ATCC 27815*
Ureaplasma urealyticum serovar 10 ATCC 33699	*Ureaplasma parvum serovar 3 ATCC 700970*
Variovorax paradoxus	*Vibrio cholerae O1*
Verminephrobacter eiseniae	*Vibrio sp. Ex25*
Vibrio fischeri	*Vibrio harveyi*
Candidatus Vesicomyosocius okutanii	*Vibrio parahaemolyticus*
Aliivibrio salmonicida LFI1238	*Vibrio splendidus*
Vibrio vulnificus CMCP6	*Wolbachia wBm*
Wigglesworthia glossinidia	*Wolbachia wMel*
Wolbachia pipientis	*Wolbachia sp. wRi*
Wolinella succinogenes	*Xanthomonas axonopodis*
Xanthobacter autotrophicus	*Xanthomonas campestris pv. campestris ATCC 33913*
Xylella fastidiosa 9a5c	*Xanthomonas oryzae KACC10331*
Yersinia enterocolitica	*Yersinia pestis CO92*
Yersinia pseudotuberculosis IP32953	*Zymomonas mobilis*
