# Supplementary material for: Genetic Co-Occurrence Network across Sequenced Microbes
Source: PLoS Comput Biol. 2011 Dec 29;7(12):e1002340. doi: 10.1371/journal.pcbi.1002340 (PMC3248385; doi:10.1371/journal.pcbi.1002340)
Supplement: Table S2 — Genes of the largest or smallest Sip's and Sin's. (PDF) [file pcbi.1002340.s005.pdf]

Table S2. Genes of the largest or smallest S_i^p's and S_i^n's.

Ten largest S_i^p's				***Ten largest S_i^n's***			
**KEGG identifier**	**Name**	**Description**	S_i^p	**KEGG identifier**	**Name**	**Description**	S_i^n
K02919	RP-L36, rpmJ	large subunit ribosomal protein L36	14.76	K02919	RP-L36, rpmJ	large subunit ribosomal protein L36	14.87
K07473	dinJ	DNA-damage-inducible protein J	14.44	K07473	dinJ	DNA-damage-inducible protein J	14.15
K07075	K07075		14.42	K06904	K06904		14.14
K07491	K07491	putative transposase	14.32	K01854	E5.4.99.9, glf	UDP-galactopyranose mutase	13.78
K05521	draG	ADP-ribosylglycohydrolase	14.27	K07075	K07075		13.75
K03453	TC.BASS	bile acid:Na+ symporter, BASS family	14.22	K00558	E2.1.1.37, DNMT, dcm	DNA (cytosine-5-)-methyltransferase	13.71
K08998	K08998	hypothetical protein	14.18	K06223	dam	DNA adenine methylase	13.59
K06223	dam	DNA adenine methylase	14.18	K00571	E2.1.1.72	site-specific DNA-methyltransferase (adenine-specific)	13.48
K00558	E2.1.1.37, DNMT, dcm	DNA (cytosine-5-)-methyltransferase	14.14	K07407	E3.2.1.22B, galA, rafA	alpha-galactosidase	13.37
K06867	K06867		14.13	K05521	draG	ADP-ribosylglycohydrolase	13.36

Ten smallest S_i^p's				***Ten smallest S_i^n's***			
**KEGG identifier**	**Name**	**Description**	S_i^p	**KEGG identifier**	**Name**	**Description**	S_i^n
K09987	K09987	hypothetical protein	3.58	K02408	fliE	flagellar hook-basal body complex protein FliE	2.78
K02390	flgE	flagellar hook protein FlgE	3.82	K02390	flgE	flagellar hook protein FlgE	2.78
K02408	fliE	flagellar hook-basal body complex protein FliE	3.84	K02401	flhB	flagellar biosynthetic protein FlhB	2.89
K02401	flhB	flagellar biosynthetic protein FlhB	3.88	K02421	fliR	flagellar biosynthetic protein FliR	3.00
K02388	flgC	flagellar basal-body rod protein FlgC	4.02	K02388	flgC	flagellar basal-body rod protein FlgC	3.02
K02421	fliR	flagellar biosynthetic protein FliR	4.02	K02420	fliQ	flagellar biosynthetic protein FliQ	3.16
K03600	sspB	stringent starvation protein B	4.06	K02417	fliNY, fliN	flagellar motor switch protein FliN/FliY	3.21
K02420	fliQ	flagellar biosynthetic protein FliQ	4.20	K03600	sspB	stringent starvation protein B	3.27
K02417	fliNY, fliN	flagellar motor switch protein FliN/FliY	4.22	K09987	K09987	hypothetical protein	3.49
K09895	K09895	hypothetical protein	4.49	K02419	fliP	flagellar biosynthetic protein FliP	3.50
