# Supplementary material for: Genetic Co-Occurrence Network across Sequenced Microbes
Source: PLoS Comput Biol. 2011 Dec 29;7(12):e1002340. doi: 10.1371/journal.pcbi.1002340 (PMC3248385; doi:10.1371/journal.pcbi.1002340)
Supplement: Table S3 — Details of the MRS constructed in this study. Gene i in each row is arrowed to gene j (wij>0) and gene j′ (wij′<0) in the MRS. The 1st through 3rd columns contain the annotation information of gene i, and the 5th through 8th columns contain information for genes j and j′ that receive incoming links from gene i. The annotation information of genes j and j′ is found in the rows in which the 1st column includes the same KEGG identifiers as those of genes j and j′. The 4th column contains the correlog group indices assigned to gene i. If gene i's belong to the same (different) correlog groups, they are assigned the same (different) indices in the 4th column. (PDF) [file pcbi.1002340.s006.pdf]

Table S3. Details of the MRS constructed in this study. Gene *i* in each row is arrowed to gene *j* (w_{ij}>0) and gene *j'* ($w_{ij'}$<0) in the MRS. The 1st through 3rd columns contain the annotation information of gene *i*, and the 5th through 8th columns contain information for genes *j* and *j'* that receive incoming links from gene *i*. The annotation information of genes *j* and *j'* is found in the rows in which the 1st column includes the same KEGG identifiers as those of genes *j* and *j'*. The 4th column contains the correlog group indices assigned to gene *i*. If gene *i*'s belong to the same (different) correlog groups, they are assigned the same (different) indices in the 4th column.

KEGG identifier (gene *i*)	Name	Description	Correlog group	KEGG identifier (gene *j*)	w_{ij}	KEGG identifier (gene *j'*)	$w_{ij'}$
K00001	E1.1.1.1, adh	alcohol dehydrogenase	G0	K04072	0.0870	K01493	-0.0568
K00003	E1.1.1.3	homoserine dehydrogenase	G1	K01733	0.1681	K12524	-0.0586
K00005	E1.1.1.6, gldA	glycerol dehydrogenase	G2	K02005	0.0625	K03502	-0.0651
K00009	mtlD	mannitol-1-phosphate 5-dehydrogenase	G3	K02798	0.1664	K01914	-0.0493
K00010	E1.1.1.18, iolG	myo-inositol 2-dehydrogenase	G4	K03335	0.1705	K07335	-0.0515
K00012	E1.1.1.22, ugd	UDPglucose 6-dehydrogenase	G5	K02445	0.0543	K01529	-0.0662
K00013	E1.1.1.23, hisD	histidinol dehydrogenase	G6	K02500	0.1014	K09790	-0.0242
K00014	E1.1.1.25, aroE	shikimate 5-dehydrogenase	G7	K01736	0.0923	K07335	-0.0460
K00016	LDH, ldh	L-lactate dehydrogenase	G8	K08483	0.0586	K08641	-0.0610
K00019	E1.1.1.30, bdh	3-hydroxybutyrate dehydrogenase	G9	K01055	0.0630	K06940	-0.0591
K00020	E1.1.1.31, mmsB	3-hydroxyisobutyrate dehydrogenase	G10	K01560	0.0636	K07778	-0.0543
K00022	E1.1.1.35, fadB	3-hydroxyacyl-CoA dehydrogenase	G11	K01692	0.1122	K03116	-0.0461
K00023	E1.1.1.36, phbB	acetoacetyl-CoA reductase	G12	K00626	0.1191	K07390	-0.0481
K00025	E1.1.1.37A	malate dehydrogenase	G13	K07749	0.0528	K00855	-0.0533
K00026	E1.1.1.37B, mdh	malate dehydrogenase	G14	K00239	0.0522	K07223	-0.0555
K00027	E1.1.1.38, sfcA, maeA	malate dehydrogenase (oxaloacetate-decarboxylating)	G15	K07319	0.0636	K00029	-0.0615
K00029	E1.1.1.40, maeB	malate dehydrogenase (oxaloacetate-decarboxylating)(NADP+)	G16	K01623	0.0468	K00027	-0.0615
K00030	IDH3	isocitrate dehydrogenase (NAD+)	G17	K01273	0.0669	K00031	-0.1644
K00031	IDH1, IDH2, icd	isocitrate dehydrogenase	G18	K01647	0.1103	K00030	-0.1644
K00033	E1.1.1.44, PGD, gnd	6-phosphogluconate dehydrogenase	G19	K00036	0.1661	K00077	-0.0514
K00036	G6PD, zwf	glucose-6-phosphate 1-dehydrogenase	G19	K00033	0.1661	K07304	-0.0481
K00040	E1.1.1.57, uxuB	fructuronate reductase	G21	K01686	0.0830	K00860	-0.0552
K00042	E1.1.1.60, garR	2-hydroxy-3-oxopropionate reductase	G22	K01706	0.0582	K05782	-0.0882

KEGG identifier (gene *i*)	Name	Description	Correlog group	KEGG identifier (gene *j*)	w_{ij}	KEGG identifier (gene *j'*)	$w_{ij'}$
K00046	E1.1.1.69, idnO	gluconate 5-dehydrogenase	G21	K01686	0.0721	K07126	-0.0659
K00050	E1.1.1.81, ttuD	hydroxypyruvate reductase	G24	K03741	0.0558	K00865	-0.0686
K00052	E1.1.1.85, leuB	3-isopropylmalate dehydrogenase	G25	K01703	0.0935	K00754	-0.0314
K00053	E1.1.1.86, ilvC	ketol-acid reductoisomerase	G26	K01687	0.0807	K03652	-0.0390
K00056	E1.1.1.93, ttuC	tartrate dehydrogenase	G27	K07246	0.2020	K09930	-0.0441
K00058	E1.1.1.95, serA	D-3-phosphoglycerate dehydrogenase	G28	K07238	0.0615	K09946	-0.0574
K00060	E1.1.1.103, tdh	threonine 3-dehydrogenase	G29	K00639	0.1366	K01479	-0.0450
K00065	E1.1.1.125, kduD	2-deoxy-D-gluconate 3-dehydrogenase	G30	K07407	0.0778	K02663	-0.0541
K00067	rfbD	dTDP-4-dehydrorhamnose reductase	G31	K01790	0.2417	K01269	-0.0503
K00074	E1.1.1.157, paaH	3-hydroxybutyryl-CoA dehydrogenase	G21	K01812	0.0541	K09117	-0.0538
K00077	E1.1.1.169, apbA	2-dehydropantoate 2-reductase	G33	K02019	0.0606	K07473	-0.0565
K00087	E1.17.1.4, xdhA, xdhB	xanthine dehydrogenase	G34	K01487	0.0944	K06013	-0.0544
K00097	E1.1.1.262, pdxA	4-hydroxythreonine-4-phosphate dehydrogenase	G35	K03474	0.0587	K10543	-0.0523
K00099	dxr	1-deoxy-D-xylulose-5-phosphate reductoisomerase	G36	K03526	0.1236	K01823	-0.0462
K00100	E1.1.1.-		G37	K00257	0.0812	K07282	-0.0673
K00101	E1.1.2.3, lldD	L-lactate dehydrogenase (cytochrome)	G38	K11209	0.0567	K00782	-0.0761
K00102	E1.1.2.4, dld	D-lactate dehydrogenase (cytochrome)	G39	K05343	0.0773	K00540	-0.0558
K00104	glcD	glycolate oxidase	G40	K11472	0.0679	K03777	-0.0529
K00108	E1.1.99.1, betA, CHDH	choline dehydrogenase	G41	K00130	0.1568	K01753	-0.0472
K00111	glpA, glpD	glycerol-3-phosphate dehydrogenase	G42	K00864	0.2033	K05801	-0.0583
K00116	E1.1.99.16, mqo	malate dehydrogenase (acceptor)	G43	K02472	0.0659	K01551	-0.0531
K00121	E1.1.1.284	S-(hydroxymethyl)glutathione dehydrogenase	G38	K03929	0.0903	K01826	-0.0632
K00123	E1.2.1.2A	formate dehydrogenase, alpha subunit	G45	K02379	0.1149	K07075	-0.0464
K00124	E1.2.1.2B1	formate dehydrogenase, beta subunit	G46	K00127	0.1044	K01077	-0.0520
K00127	E1.2.1.2G	formate dehydrogenase, gamma subunit	G46	K00124	0.1044	K00520	-0.0487
K00128	E1.2.1.3	aldehyde dehydrogenase (NAD+)	G0	K00001	0.0653	K00721	-0.0469

KEGG identifier (gene *i*)	Name	Description	Correlog group	KEGG identifier (gene *j*)	w_{ij}	KEGG identifier (gene *j'*)	$w_{ij'}$
K00130	E1.2.1.8, betB, gbsA	betaine-aldehyde dehydrogenase	G41	K00108	0.1568	K05993	-0.0655
K00135	E1.2.1.16, gabD	succinate-semialdehyde dehydrogenase (NADP+)	G50	K01207	0.0858	K00599	-0.0537
K00140	E1.2.1.27, mmsA, iolA	methylmalonate-semialdehyde dehydrogenase	G4	K03336	0.0783	K09017	-0.0410
K00145	argC	N-acetyl-gamma-glutamyl-phosphate reductase	G52	K00930	0.1677	K03569	-0.0358
K00147	proA	glutamate-5-semialdehyde dehydrogenase	G53	K00931	0.1813	K01858	-0.0500
K00151	E1.2.1.60, hpaE	5-carboxymethyl-2-hydroxymuconic-semialdehyde dehydrogenase	G54	K02509	0.1719	K03192	-0.0544
K00154	E1.2.1.68	coniferyl-aldehyde dehydrogenase	G55	K01442	0.0824	K00302	-0.0611
K00156	E1.2.2.2, poxB	pyruvate dehydrogenase (cytochrome)	G30	K00065	0.0585	K03287	-0.0716
K00161	PDHA, pdhA	pyruvate dehydrogenase E1 component subunit alpha	G8	K00162	0.3086	K03098	-0.0411
K00162	PDHB, pdhB	pyruvate dehydrogenase E1 component subunit beta	G8	K00161	0.3086	K07122	-0.0347
K00163	aceE	pyruvate dehydrogenase E1 component	G59	K06907	0.0342	K00721	-0.0353
K00164	OGDH, sucA	2-oxoglutarate dehydrogenase E1 component	G60	K00658	0.1093	K00660	-0.0297
K00166	E1.2.4.4A, bkdA1	2-oxoisovalerate dehydrogenase E1 component, alpha subunit	G61	K00167	0.1963	K09967	-0.0267
K00167	E1.2.4.4B, bkdA2	2-oxoisovalerate dehydrogenase E1 component, beta subunit	G61	K00166	0.1963	K01176	-0.0276
K00174	korA	2-oxoglutarate ferredoxin oxidoreductase subunit alpha	G63	K00175	0.2411	K05595	-0.0369
K00175	korB	2-oxoglutarate ferredoxin oxidoreductase subunit beta	G63	K00174	0.2411	K05595	-0.0369
K00176	korD	2-oxoglutarate ferredoxin oxidoreductase subunit delta	G65	K00177	0.1391	K03091	-0.0476
K00177	korG	2-oxoglutarate ferredoxin oxidoreductase subunit gamma	G65	K00176	0.1391	K03328	-0.0317
K00179	E1.2.7.8A, iorA	indolepyruvate ferredoxin oxidoreductase, alpha subunit	G67	K00180	0.3251	K06016	-0.0441
K00180	E1.2.7.8B, iorB	indolepyruvate ferredoxin oxidoreductase, beta subunit	G67	K00179	0.3251	K00705	-0.0408
K00208	fabI	enoyl-[acyl-carrier protein] reductase I	G69	K09015	0.0453	K07006	-0.0478
K00210	E1.3.1.12	prephenate dehydrogenase	G70	K04092	0.0597	K04517	-0.0951
K00215	dapB	dihydrodipicolinate reductase	G71	K01417	0.0737	K00564	-0.0541
K00219	E1.3.1.34, fadH	2,4-dienoyl-CoA reductase (NADPH2)	G72	K00883	0.0623	K07458	-0.0689
K00228	E1.3.3.3, hemF	coproporphyrinogen III oxidase	G73	K08994	0.0278	K09162	-0.0312
K00230	E1.3.3.4A, hemG	protoporphyrinogen oxidase	G30	K00065	0.0438	K03809	-0.0440

KEGG identifier (gene *i*)	Name	Description	Correlog group	KEGG identifier (gene *j*)	w_{ij}	KEGG identifier (gene *j'*)	$w_{ij'}$
K00231	E1.3.3.4B, hemY	protoporphyrinogen oxidase	G75	K01772	0.0556	K08973	-0.0568
K00239	sdhA	succinate dehydrogenase flavoprotein subunit	G14	K00240	0.1326	K01262	-0.0339
K00240	sdhB	succinate dehydrogenase iron-sulfur protein	G14	K00239	0.1326	K09686	-0.0483
K00241	sdhC	succinate dehydrogenase cytochrome b-556 subunit	G14	K00239	0.0998	K06044	-0.0352
K00242	sdhD	succinate dehydrogenase hydrophobic membrane anchor protein	G14	K00241	0.0471	K06016	-0.0354
K00243	K00243	hypothetical protein	G80	K07139	0.0512	K04084	-0.0420
K00244	frdA	fumarate reductase flavoprotein subunit	G81	K00246	0.1288	K00019	-0.0511
K00245	frdB	fumarate reductase iron-sulfur protein	G81	K00246	0.2120	K01175	-0.0387
K00246	frdC	fumarate reductase subunit C	G81	K00245	0.2120	K07275	-0.0363
K00248	E1.3.99.2, bcd	butyryl-CoA dehydrogenase	G84	K02106	0.0820	K03312	-0.0587
K00249	E1.3.99.3, ACADM, acd	acyl-CoA dehydrogenase	G85	K07213	0.0878	K07645	-0.0546
K00252	E1.3.99.7, gcdH	glutaryl-CoA dehydrogenase	G86	K01966	0.0506	K03316	-0.0438
K00253	E1.3.99.10, ivd	isovaleryl-CoA dehydrogenase	G87	K04090	0.0654	K09017	-0.0351
K00257	E1.3.99.-		G37	K00100	0.0812	K01438	-0.0529
K00259	ald	alanine dehydrogenase	G89	K01784	0.0650	K01618	-0.0508
K00260	E1.4.1.2	glutamate dehydrogenase	G90	K05569	0.0705	K03762	-0.0629
K00261	E1.4.1.3	glutamate dehydrogenase (NAD(P)+)	G37	K00100	0.0658	K03060	-0.0594
K00262	E1.4.1.4, gdhA	glutamate dehydrogenase (NADP+)	G92	K03446	0.0548	K08987	-0.0707
K00263	E1.4.1.9	leucine dehydrogenase	G93	K00453	0.0641	K06131	-0.0469
K00265	gltB	glutamate synthase (NADPH/NADH) large chain	G94	K00266	0.1308	K07106	-0.0472
K00266	gltD	glutamate synthase (NADPH/NADH) small chain	G94	K00265	0.1308	K01176	-0.0491
K00274	E1.4.3.4	monoamine oxidase	G96	K09959	0.0733	K03824	-0.0518
K00275	E1.4.3.5, pdxH	pyridoxamine 5'-phosphate oxidase	G35	K03474	0.0563	K05794	-0.0504
K00278	nadB	L-aspartate oxidase	G98	K03517	0.1590	K01081	-0.0465
K00282	E1.4.4.2A, gcvPA	glycine dehydrogenase subunit 1	G99	K00283	0.1841	K08984	-0.0290
K00283	E1.4.4.2B, gcvPB	glycine dehydrogenase subunit 2	G99	K00282	0.1841	K08984	-0.0290
K00285	dadA	D-amino-acid dehydrogenase	G101	K00344	0.0601	K03534	-0.0739
K00286	E1.5.1.2, proC	pyrroline-5-carboxylate reductase	G53	K00931	0.0790	K03555	-0.0407
K00287	E1.5.1.3, folA	dihydrofolate reductase	G103	K00560	0.1358	K03465	-0.0652

KEGG identifier (gene *i*)	Name	Description	Correlog group	KEGG identifier (gene *j*)	w_{ij}	KEGG identifier (gene *j'*)	$w_{ij'}$
K00294	E1.5.1.12, putA	1-pyrroline-5-carboxylate dehydrogenase	G104	K00318	0.1939	K05895	-0.0332
K00297	E1.5.1.20, metF	methylenetetrahydrofolate reductase (NADPH)	G71	K00215	0.0691	K00547	-0.0617
K00299	E1.5.1.29	FMN reductase	G106	K04091	0.0766	K07185	-0.0603
K00302	soxA	sarcosine oxidase, subunit alpha	G107	K06999	0.0665	K00836	-0.0650
K00311	E1.5.5.1, etf	electron-transferring-flavoprotein dehydrogenase	G108	K03522	0.0670	K06888	-0.0425
K00318	E1.5.99.8, putA	proline dehydrogenase	G104	K00294	0.1939	K07010	-0.0461
K00322	E1.6.1.1, udhA	NAD(P) transhydrogenase	G21	K00040	0.0509	K07095	-0.0494
K00324	E1.6.1.2A, pntA	NAD(P) transhydrogenase subunit alpha	G111	K00325	0.1836	K01081	-0.0406
K00325	E1.6.1.2B, pntB	NAD(P) transhydrogenase subunit beta	G111	K00324	0.1836	K07391	-0.0690
K00329	E1.6.5.3	NADH dehydrogenase	G113	K00356	0.2388	K01485	-0.0452
K00330	nuoA	NADH dehydrogenase I subunit A	G114	K00340	0.0671	K03821	-0.0583
K00331	nuoB	NADH dehydrogenase I subunit B	G115	K00337	0.0938	K00818	-0.0237
K00332	nuoC	NADH dehydrogenase I subunit C	G116	K00333	0.0937	K07403	-0.0220
K00333	nuoD	NADH dehydrogenase I subunit D	G116	K00332	0.0937	K04751	-0.0192
K00334	nuoE	NADH dehydrogenase I subunit E	G118	K00335	0.1497	K09794	-0.0383
K00335	nuoF	NADH dehydrogenase I subunit F	G118	K00334	0.1497	K07154	-0.0337
K00336	nuoG	NADH dehydrogenase I subunit G	G118	K00335	0.1107	K03928	-0.0320
K00337	nuoH	NADH dehydrogenase I subunit H	G115	K00331	0.0938	K00818	-0.0240
K00338	nuoI	NADH dehydrogenase I subunit I	G115	K00341	0.0693	K00266	-0.0262
K00339	nuoJ	NADH dehydrogenase I subunit J	G114	K00340	0.0817	K01571	-0.0259
K00340	nuoK	NADH dehydrogenase I subunit K	G114	K00339	0.0817	K01571	-0.0295
K00341	nuoL	NADH dehydrogenase I subunit L	G115	K00342	0.0753	K01572	-0.0249
K00342	nuoM	NADH dehydrogenase I subunit M	G115	K00331	0.0889	K06958	-0.0255
K00343	nuoN	NADH dehydrogenase I subunit N	G115	K00342	0.0754	K06958	-0.0248
K00344	E1.6.5.5, qor	NADPH2:quinone reductase	G101	K03762	0.0723	K10041	-0.0538
K00346	nqrA	Na+-transporting NADH:ubiquinone oxidoreductase subunit A	G129	K00347	0.1008	K01628	-0.0343
K00347	nqrB	Na+-transporting NADH:ubiquinone oxidoreductase subunit B	G129	K00349	0.1294	K01654	-0.0230
K00348	nqrC	Na+-transporting NADH:ubiquinone oxidoreductase subunit C	G129	K00347	0.1267	K01654	-0.0247

KEGG identifier (gene *i*)	Name	Description	Correlog group	KEGG identifier (gene *j*)	w_{ij}	KEGG identifier (gene *j'*)	$w_{ij'}$
K00349	nqrD	Na+-transporting NADH:ubiquinone oxidoreductase subunit D	G129	K00347	0.1294	K01654	-0.0230
K00350	nqrE	Na+-transporting NADH:ubiquinone oxidoreductase subunit E	G129	K00347	0.1294	K01654	-0.0230
K00351	nqrF	Na+-transporting NADH:ubiquinone oxidoreductase subunit F	G129	K00347	0.1268	K01654	-0.0209
K00356	E1.6.99.3	NADH dehydrogenase	G113	K00329	0.2388	K00547	-0.0567
K00362	E1.7.1.4L, nirB	nitrite reductase (NAD(P)H) large subunit	G136	K00363	0.2065	K07334	-0.0409
K00363	E1.7.1.4S, nirD	nitrite reductase (NAD(P)H) small subunit	G136	K00362	0.2065	K08224	-0.0568
K00364	E1.7.1.7, guaC	GMP reductase	G138	K01226	0.0694	K01079	-0.0636
K00366	E1.7.7.1, nirA	ferredoxin-nitrite reductase	G139	K05375	0.0813	K07141	-0.0435
K00370	narG	nitrate reductase 1, alpha subunit	G140	K00371	0.2335	K01854	-0.0274
K00371	narH	nitrate reductase 1, beta subunit	G140	K00370	0.2335	K07075	-0.0342
K00372	E1.7.99.4C	nitrate reductase catalytic subunit	G136	K00363	0.1074	K06044	-0.0675
K00373	narJ	nitrate reductase 1, delta subunit	G143	K00374	0.2111	K00836	-0.0348
K00374	narI	nitrate reductase 1, gamma subunit	G143	K00373	0.2111	K07737	-0.0279
K00375	K00375	GntR family transcriptional regulator	G145	K07005	0.0824	K01992	-0.0581
K00378	hcp	hydroxylamine reductase	G146	K06871	0.0522	K03297	-0.0417
K00380	E1.8.1.2A, cysJ	sulfite reductase (NADPH) flavoprotein alpha-component	G147	K00381	0.0698	K05993	-0.0496
K00381	E1.8.1.2B, cysI	sulfite reductase (NADPH) hemoprotein beta-component	G147	K00390	0.0937	K01436	-0.0456
K00383	E1.8.1.7, GSR, gor	glutathione reductase (NADPH)	G149	K07171	0.0626	K07458	-0.0582
K00389	yidH	putative membrane protein	G150	K06876	0.0878	K03306	-0.0762
K00390	E1.8.4.8, cysH	phosphoadenosine phosphosulfate reductase	G147	K00381	0.0937	K07458	-0.0416
K00404	ccoN	cb-type cytochrome c oxidase subunit I	G152	K00405	0.2060	K05343	-0.0431
K00405	ccoO	cb-type cytochrome c oxidase subunit II	G152	K00404	0.2060	K07122	-0.0370
K00406	ccoP	cb-type cytochrome c oxidase subunit III	G152	K00405	0.1034	K01623	-0.0375
K00407	ccoQ	cb-type cytochrome c oxidase subunit IV	G146	K00837	0.0584	K10680	-0.0559
K00411	RIP1, UQCRFS1, petA	ubiquinol-cytochrome c reductase iron-sulfur subunit	G156	K00412	0.1068	K02380	-0.0339
K00412	CYTB, petB	ubiquinol-cytochrome c reductase cytochrome b subunit	G156	K00411	0.1068	K02380	-0.0402
K00413	CYT1, CYC1, petC	ubiquinol-cytochrome c reductase cytochrome c1 subunit	G156	K00411	0.0909	K00528	-0.0331

KEGG identifier (gene *i*)	Name	Description	Correlog group	KEGG identifier (gene *j*)	w_{ij}	KEGG identifier (gene *j'*)	$w_{ij'}$
K00425	cydA	cytochrome bd-I oxidase subunit I	G159	K00426	0.3374	K08234	-0.0450
K00426	cydB	cytochrome bd-I oxidase subunit II	G159	K00425	0.3374	K03297	-0.0317
K00428	E1.11.1.5	cytochrome c peroxidase	G161	K09939	0.0643	K03322	-0.0573
K00432	E1.11.1.9	glutathione peroxidase	G162	K06885	0.0660	K01239	-0.0685
K00448	E1.13.11.3A, pcaG	protocatechuate 3,4-dioxygenase, alpha subunit	G163	K00449	0.1663	K02472	-0.0328
K00449	E1.13.11.3B, pcaH	protocatechuate 3,4-dioxygenase, beta subunit	G163	K00448	0.1663	K06904	-0.0649
K00451	E1.13.11.5, hmgA	homogentisate 1,2-dioxygenase	G165	K01555	0.1021	K01912	-0.0429
K00453	E1.13.11.11, TDO2	tryptophan 2,3-dioxygenase	G93	K01556	0.1717	K02022	-0.0425
K00457	HPD, hppD	4-hydroxyphenylpyruvate dioxygenase	G167	K00500	0.0695	K07010	-0.0357
K00459	E1.13.11.32	2-nitropropane dioxygenase	G17	K01273	0.0780	K02168	-0.0535
K00481	E1.14.13.2, pobA	p-hydroxybenzoate 3-monooxygenase	G163	K00448	0.1078	K11177	-0.0469
K00500	phhA, PAH	phenylalanine-4-hydroxylase	G167	K00457	0.0695	K01011	-0.0460
K00507	SCD, desC	stearoyl-CoA desaturase (delta-9 desaturase)	G96	K06978	0.0505	K01788	-0.0428
K00520	merA	mercuric reductase	G172	K08234	0.0614	K07341	-0.0796
K00523	ascD, ddhD, rfbI	CDP-4-dehydro-6-deoxyglucose reductase	G173	K01709	0.0518	K05982	-0.0494
K00526	E1.17.4.1B, nrdB, nrdF	ribonucleoside-diphosphate reductase beta chain	G174	K03647	0.1610	K03087	-0.0624
K00527	nrdD	ribonucleoside-triphosphate reductase	G175	K04068	0.0804	K00837	-0.0470
K00528	E1.18.1.2, fpr	ferredoxin--NADP+ reductase	G94	K00266	0.0663	K03327	-0.0611
K00537	ARSC1, arsC	arsenate reductase	G2	K00974	0.0637	K01744	-0.0472
K00540	E1.-.-.-		G146	K00754	0.0956	K00102	-0.0558
K00547	E2.1.1.10, mmuM	homocysteine S-methyltransferase	G179	K01188	0.0814	K07118	-0.0677
K00548	E2.1.1.13, metH	5-methyltetrahydrofolate--homocysteine methyltransferase	G180	K00798	0.0712	K03707	-0.0427
K00549	E2.1.1.14, metE	5-methyltetrahydropteroyltriglutamate--homocysteine	G181	K03576	0.0571	K03737	-0.0532
K00556	trmH	tRNA (guanosine-2'-O-)-methyltransferase	G182	K06287	0.0599	K00836	-0.0518
K00557	trmA	tRNA (uracil-5-)-methyltransferase	G183	K01854	0.0670	K07319	-0.0866
K00558	E2.1.1.37, DNMT, dcm	DNA (cytosine-5-)-methyltransferase	G184	K07458	0.1726	K00754	-0.0815
K00560	E2.1.1.45, thyA	thymidylate synthase	G103	K00287	0.1358	K03465	-0.1122

KEGG identifier (gene *i*)	Name	Description	Correlog group	KEGG identifier (gene *j*)	w_{ij}	KEGG identifier (gene *j'*)	$w_{ij'}$
K00563	E2.1.1.51, rrmA	rRNA (guanine-N1-)-methyltransferase	G186	K07146	0.0561	K07170	-0.0499
K00564	rsmC	ribosomal RNA small subunit methyltransferase C	G187	K05993	0.0539	K07118	-0.0637
K00567	E2.1.1.63, MGMT, ogt, ada	methylated-DNA-[protein]-cysteine S-methyltransferase	G188	K02428	0.1088	K03821	-0.0865
K00568	ubiG	3-demethylubiquinone-9 3-methyltransferase	G189	K03198	0.1419	K01154	-0.0432
K00571	E2.1.1.72	site-specific DNA-methyltransferase (adenine-specific)	G190	K01529	0.0825	K00971	-0.0619
K00573	E2.1.1.77, pcm	protein-L-isoaspartate(D-aspartate) O-methyltransferase	G107	K01697	0.0444	K06183	-0.0422
K00574	E2.1.1.79, cfa	cyclopropane-fatty-acyl-phospholipid synthase	G150	K09701	0.0653	K06988	-0.0613
K00575	cheR	chemotaxis protein methyltransferase CheR	G193	K03412	0.1669	K01473	-0.0329
K00593	cobI	precorrin-2 C20-methyltransferase	G194	K00595	0.1375	K01791	-0.0450
K00595	cobL	precorrin-6Y C5,15-methyltransferase	G194	K00593	0.1375	K06929	-0.0477
K00598	tam	trans-aconitate 2-methyltransferase	G196	K01464	0.0908	K05966	-0.0635
K00599	E2.1.1.-		G197	K08998	0.0828	K01188	-0.0729
K00605	E2.1.2.10, gcvT	aminomethyltransferase	G99	K00282	0.1506	K01442	-0.0274
K00606	E2.1.2.11, panB	3-methyl-2-oxobutanoate hydroxymethyltransferase	G199	K01918	0.1778	K07021	-0.0525
K00611	E2.1.3.3, OTC, argF, argI	ornithine carbamoyltransferase	G200	K00926	0.1367	K00824	-0.0520
K00616	E2.2.1.2, talA, talB	transaldolase	G36	K00919	0.0707	K07507	-0.0505
K00619	E2.3.1.1A, argA	amino-acid N-acetyltransferase	G202	K00973	0.0414	K01601	-0.0452
K00620	argJ	glutamate N-acetyltransferase	G52	K00145	0.1129	K01089	-0.0457
K00625	E2.3.1.8, pta	phosphate acetyltransferase	G204	K06873	0.1515	K01175	-0.0552
K00626	E2.3.1.9, atoB	acetyl-CoA C-acetyltransferase	G12	K00023	0.1191	K00930	-0.0478
K00627	DLAT, aceF, pdhC	pyruvate dehydrogenase E2 component (dihydrolipoamide	G8	K00162	0.1374	K07182	-0.0530
K00631	E2.3.1.15B, plsB	glycerol-3-phosphate O-acyltransferase	G207	K03583	0.0411	K03621	-0.0357
K00632	E2.3.1.16, fadA	acetyl-CoA acyltransferase	G11	K00022	0.0672	K07185	-0.0544
K00639	E2.3.1.29, kbl	glycine C-acetyltransferase	G29	K00060	0.1366	K01531	-0.0520
K00640	E2.3.1.30, cysE	serine O-acetyltransferase	G210	K01738	0.0992	K07010	-0.0521
K00641	E2.3.1.31, metX	homoserine O-acetyltransferase	G211	K01740	0.0859	K00651	-0.1101

KEGG identifier (gene *i*)	Name	Description	Correlog group	KEGG identifier (gene *j*)	w_{ij}	KEGG identifier (gene *j'*)	$w_{ij'}$
K00643	E2.3.1.37, ALAS	5-aminolevulinate synthase	G139	K00812	0.0342	K02168	-0.0301
K00647	fabB	3-oxoacyl-[acyl-carrier-protein] synthase I	G213	K01716	0.0529	K00428	-0.0434
K00651	metA	homoserine O-succinyltransferase	G211	K01740	0.0847	K00641	-0.1101
K00652	bioF	8-amino-7-oxononanoate synthase	G215	K01935	0.1309	K11754	-0.0479
K00656	E2.3.1.54, pflD	formate C-acetyltransferase	G216	K04069	0.1421	K07185	-0.0415
K00657	E2.3.1.57, speG	diamine N-acetyltransferase	G217	K07050	0.0594	K03317	-0.0653
K00658	DLST, sucB	2-oxoglutarate dehydrogenase E2 component (dihydrolipoamide	G60	K00164	0.1093	K00660	-0.0301
K00660	E2.3.1.74, bcsA	chalcone synthase	G219	K02074	0.0635	K01560	-0.0524
K00661	E2.3.1.79, maa	maltose O-acetyltransferase	G22	K07727	0.0780	K00854	-0.0639
K00666	K00666	fatty-acyl-CoA synthase	G221	K03297	0.0759	K03762	-0.0568
K00673	astA	arginine N-succinyltransferase	G222	K01484	0.1168	K07341	-0.0369
K00674	dapD	2,3,4,5-tetrahydropyridine-2-carboxylate N-succinyltransferase	G223	K01778	0.0952	K03856	-0.0419
K00677	lpxA	UDP-N-acetylglucosamine acyltransferase	G224	K00748	0.0630	K01295	-0.0369
K00680	E2.3.1.-		G146	K00754	0.1000	K03892	-0.0783
K00681	ggt	gamma-glutamyltranspeptidase	G226	K00839	0.0596	K02823	-0.0540
K00684	aat	leucyl/phenylalanyl-tRNA--protein transferase	G227	K06891	0.0688	K01476	-0.0349
K00685	E2.3.2.8, ate1	arginine-tRNA-protein transferase	G228	K07336	0.0323	K01654	-0.0310
K00688	E2.4.1.1, glgP, PYG	starch phosphorylase	G229	K00703	0.1253	K01486	-0.0383
K00694	bcsA	cellulose synthase (UDP-forming)	G230	K01179	0.1199	K09472	-0.0555
K00697	E2.4.1.15, otsA	alpha,alpha-trehalose-phosphate synthase (UDP-forming)	G231	K01087	0.1895	K09939	-0.0379
K00700	E2.4.1.18, glgB	1,4-alpha-glucan branching enzyme	G229	K00975	0.1383	K07112	-0.0415
K00703	E2.4.1.21, glgA	starch synthase	G229	K00975	0.1629	K03321	-0.0423
K00705	E2.4.1.25, malQ	4-alpha-glucanotransferase	G39	K06044	0.0815	K05801	-0.0689
K00721	DPM1	dolichol-phosphate mannosyltransferase	G235	K00966	0.0871	K07141	-0.0741
K00748	lpxB	lipid-A-disaccharide synthase	G224	K00677	0.0630	K01990	-0.0171
K00754	E2.4.1.-		G146	K01423	0.1360	K00558	-0.0815
K00756	E2.4.2.2, pdp	pyrimidine-nucleoside phosphorylase	G238	K01619	0.0689	K00758	-0.1817
K00757	E2.4.2.3, udp	uridine phosphorylase	G239	K02647	0.0603	K06013	-0.0621
K00758	E2.4.2.4, deoA	thymidine phosphorylase	G240	K05812	0.0672	K00756	-0.1817
K00759	E2.4.2.7, apt	adenine phosphoribosyltransferase	G241	K00761	0.0526	K09800	-0.0474

KEGG identifier (gene *i*)	Name	Description	Correlog group	KEGG identifier (gene *j*)	w_{ij}	KEGG identifier (gene *j'*)	$w_{ij'}$
K00760	E2.4.2.8, hpt	hypoxanthine phosphoribosyltransferase	G13	K03295	0.0518	K07146	-0.0421
K00761	E2.4.2.9, upp	uracil phosphoribosyltransferase	G241	K03602	0.0694	K03325	-0.0460
K00763	E2.4.2.11, pncB	nicotinate phosphoribosyltransferase	G244	K01440	0.0670	K09760	-0.0640
K00765	E2.4.2.17, hisG	ATP phosphoribosyltransferase	G6	K00013	0.0926	K00936	-0.0336
K00766	E2.4.2.18, trpD	anthranilate phosphoribosyltransferase	G246	K01609	0.1446	K02433	-0.0489
K00767	E2.4.2.19, nadC	nicotinate-nucleotide pyrophosphorylase (carboxylating)	G98	K03517	0.1904	K07213	-0.0445
K00768	E2.4.2.21, cobU, cobT	nicotinate-nucleotide--dimethylbenzimidazole	G248	K02233	0.1318	K10778	-0.0327
K00769	gpt	xanthine phosphoribosyltransferase	G249	K00852	0.0461	K07334	-0.0410
K00772	E2.4.2.28, mtaP	5'-methylthioadenosine phosphorylase	G250	K09931	0.0819	K01243	-0.0642
K00782	ykgG	hypothetical protein	G251	K03303	0.1143	K00101	-0.0761
K00783	ybeA	hypothetical protein	G252	K04094	0.0549	K02055	-0.0441
K00784	E3.1.26.11, elaC	ribonuclease Z	G13	K07313	0.0549	K03322	-0.0558
K00788	E2.5.1.3, thiE	thiamine-phosphate pyrophosphorylase	G254	K00878	0.1662	K05515	-0.0556
K00793	E2.5.1.9A, ribE	riboflavin synthase alpha chain	G255	K11752	0.1405	K01537	-0.0569
K00794	ribH	riboflavin synthase beta chain	G255	K00793	0.1354	K01222	-0.0358
K00795	E2.5.1.10, ispA	geranyltranstransferase	G257	K07011	0.0767	K03409	-0.0571
K00796	E2.5.1.15, folP	dihydropteroate synthase	G162	K00950	0.2163	K01255	-0.0622
K00797	E2.5.1.16, SRM, speE	spermidine synthase	G259	K01611	0.1701	K07734	-0.0547
K00798	E2.5.1.17, cobO, btuR	cob(I)alamin adenosyltransferase	G180	K00548	0.0712	K00558	-0.0459
K00799	E2.5.1.18, gst	glutathione S-transferase	G189	K00970	0.0577	K09159	-0.0586
K00805	E2.5.1.30	trans-hexaprenyltranstransferase	G239	K10947	0.0660	K02523	-0.0864
K00812	E2.6.1.1A, aspB	aspartate aminotransferase	G139	K05375	0.0541	K07034	-0.0530
K00813	E2.6.1.1B, aspC	aspartate aminotransferase	G241	K03602	0.0788	K00832	-0.0892
K00817	hisC	histidinol-phosphate aminotransferase	G265	K04486	0.0606	K01190	-0.0470
K00818	E2.6.1.11, argD	acetylornithine aminotransferase	G266	K03892	0.0685	K00821	-0.0657
K00819	E2.6.1.13, rocD	ornithine--oxo-acid transaminase	G235	K00721	0.0669	K00694	-0.0503
K00821	argD, dapC	acetylornithine/N-succinyldiaminopimelate aminotransferase	G268	K01069	0.0607	K00818	-0.0657

KEGG identifier (gene *i*)	Name	Description	Correlog group	KEGG identifier (gene *j*)	w_{ij}	KEGG identifier (gene *j'*)	$w_{ij'}$
K00822	E2.6.1.18	beta-alanine--pyruvate transaminase	G269	K09471	0.0593	K11747	-0.0444
K00823	E2.6.1.19, gabT	4-aminobutyrate aminotransferase	G270	K07250	0.2120	K03707	-0.0595
K00824	E2.6.1.21, dat	D-alanine transaminase	G54	K09930	0.0535	K00611	-0.0520
K00831	E2.6.1.52, serC	phosphoserine aminotransferase	G33	K00077	0.0436	K01007	-0.0415
K00832	tyrB	aromatic-amino-acid transaminase	G273	K01696	0.0595	K00813	-0.0892
K00833	E2.6.1.62, bioA	adenosylmethionine-8-amino-7-oxononanoate aminotransferase	G215	K01935	0.2343	K03523	-0.0450
K00836	E2.6.1.76, ectB	diaminobutyrate-2-oxoglutarate transaminase	G187	K01795	0.0594	K00302	-0.0650
K00837	E2.6.1.-		G146	K01423	0.0948	K01537	-0.0669
K00839	E2.6.1.-B	aminotransferase	G226	K03458	0.0760	K07021	-0.0706
K00842	E2.6.1.-E, patB	aminotransferase	G73	K00917	0.0630	K09936	-0.0595
K00845	glk	glucokinase	G279	K07305	0.0532	K00997	-0.0721
K00847	E2.7.1.4, scrK	fructokinase	G138	K01193	0.0951	K07112	-0.0480
K00849	galK	galactokinase	G281	K00965	0.0807	K00558	-0.0586
K00850	PFK, pfk	6-phosphofructokinase	G282	K01624	0.0643	K00567	-0.0579
K00851	E2.7.1.12, gntK, idnK	gluconokinase	G187	K03299	0.0595	K06896	-0.0567
K00852	E2.7.1.15, rbsK	ribokinase	G249	K01239	0.0944	K03295	-0.0568
K00854	E2.7.1.17, xylB	xylulokinase	G285	K01805	0.1556	K00661	-0.0639
K00855	E2.7.1.19, prkB	phosphoribulokinase	G286	K01601	0.1118	K00025	-0.0533
K00856	E2.7.1.20, ADK	adenosine kinase	G221	K07737	0.0410	K01791	-0.0581
K00857	E2.7.1.21, tdk	thymidine kinase	G204	K01489	0.0641	K09157	-0.0459
K00860	E2.7.1.25, cysC	adenylylsulfate kinase	G289	K00958	0.0902	K00040	-0.0552
K00864	E2.7.1.30, glpK	glycerol kinase	G42	K00111	0.2033	K01262	-0.0520
K00865	E2.7.1.31, glxK	glycerate kinase	G239	K02647	0.0540	K00050	-0.0686
K00867	coaA	type I pantothenate kinase	G292	K13038	0.0590	K03525	-0.1015
K00868	E2.7.1.35, pdxK	pyridoxine kinase	G55	K01442	0.0651	K02032	-0.0673
K00872	E2.7.1.39A, thrB	homoserine kinase	G1	K01733	0.1214	K02204	-0.0516
K00874	kdgK	2-dehydro-3-deoxygluconokinase	G295	K01625	0.1059	K03469	-0.0496
K00876	E2.7.1.48, udk	uridine kinase	G93	K03402	0.0642	K00919	-0.0639
K00877	E2.7.1.49	hydroxymethylpyrimidine kinase	G297	K00941	0.1632	K01299	-0.0467
K00878	E2.7.1.50, thiM	hydroxyethylthiazole kinase	G254	K00788	0.1662	K01464	-0.0561

KEGG identifier (gene *i*)	Name	Description	Correlog group	KEGG identifier (gene *j*)	w_{ij}	KEGG identifier (gene *j'*)	$w_{ij'}$
K00882	E2.7.1.56, fruK	1-phosphofructokinase	G299	K02770	0.1032	K09787	-0.0534
K00883	E2.7.1.58, dgoK	2-dehydro-3-deoxygalactonokinase	G72	K00219	0.0623	K08978	-0.0561
K00886	ppgK	polyphosphate glucokinase	G301	K11105	0.0494	K01077	-0.0518
K00891	E2.7.1.71, aroK	shikimate kinase	G302	K01735	0.1284	K00261	-0.0499
K00901	E2.7.1.107, DGK, dgkA	diacylglycerol kinase	G303	K07722	0.0521	K07080	-0.0480
K00906	aceK	isocitrate dehydrogenase kinase/phosphatase	G304	K03776	0.0533	K08984	-0.0469
K00912	lpxK	tetraacyldisaccharide 4'-kinase	G305	K02517	0.0503	K07473	-0.0225
K00917	E2.7.1.144, lacC	tagatose 6-phosphate kinase	G73	K08994	0.0679	K07341	-0.0572
K00919	ispE	4-diphosphocytidyl-2-C-methyl-D-erythritol kinase	G36	K00099	0.1139	K00876	-0.0639
K00925	E2.7.2.1, ackA	acetate kinase	G204	K00625	0.1466	K03284	-0.0678
K00926	arcC	carbamate kinase	G200	K01478	0.2087	K05801	-0.0733
K00930	argB	acetylglutamate kinase	G52	K00145	0.1677	K05794	-0.0568
K00931	proB	glutamate 5-kinase	G53	K00147	0.1813	K03652	-0.0397
K00936	E2.7.3.-		G312	K01362	0.0869	K02429	-0.0565
K00937	ppk	polyphosphate kinase	G146	K01514	0.0693	K07778	-0.0624
K00941	E2.7.4.7, thiD	phosphomethylpyrimidine kinase	G297	K00877	0.1632	K02488	-0.0498
K00946	E2.7.4.16, thiL	thiamine-monophosphate kinase	G315	K04773	0.0467	K00949	-0.0407
K00949	E2.7.6.2, THI80	thiamine pyrophosphokinase	G316	K07075	0.0850	K09686	-0.0615
K00950	E2.7.6.3, folK	2-amino-4-hydroxy-6-hydroxymethyldihydropteridine pyrophosphokinase	G162	K00796	0.2163	K02032	-0.0617
K00951	relA	GTP pyrophosphokinase	G162	K00796	0.0745	K01139	-0.0800
K00952	E2.7.7.1, nadR	nicotinamide-nucleotide adenylyltransferase	G190	K01529	0.1021	K00680	-0.0588
K00956	cysN	sulfate adenylyltransferase subunit 1	G320	K00957	0.1647	K00958	-0.0540
K00957	cysD	sulfate adenylyltransferase subunit 2	G320	K00956	0.1647	K00958	-0.0677
K00958	E2.7.7.4C, met3	sulfate adenylyltransferase	G289	K00860	0.0902	K00957	-0.0677
K00963	E2.7.7.9, galU	UTP--glucose-1-phosphate uridylyltransferase	G42	K00864	0.0698	K01262	-0.0481
K00965	E2.7.7.12	UDPglucose--hexose-1-phosphate uridylyltransferase	G281	K00849	0.0807	K00795	-0.0536
K00966	E2.7.7.13	mannose-1-phosphate guanylyltransferase	G235	K00721	0.0871	K01659	-0.0623
K00970	E2.7.7.19, pcnB	poly(A) polymerase	G189	K03198	0.0772	K00974	-0.1091
K00971	E2.7.7.22, manC	mannose-1-phosphate guanylyltransferase	G327	K01840	0.0705	K06044	-0.0685

KEGG identifier (gene *i*)	Name	Description	Correlog group	KEGG identifier (gene *j*)	w_{ij}	KEGG identifier (gene *j'*)	$w_{ij'}$
K00973	E2.7.7.24, rfbA	glucose-1-phosphate thymidylyltransferase	G202	K01710	0.1871	K03973	-0.0592
K00974	cca	tRNA nucleotidyltransferase (CCA-adding enzyme)	G2	K02005	0.0706	K00970	-0.1091
K00975	glgC	glucose-1-phosphate adenylyltransferase	G229	K00703	0.1629	K03300	-0.0440
K00978	rfbF	glucose-1-phosphate cytidylyltransferase	G173	K01709	0.3982	K09825	-0.0511
K00979	kdsB	3-deoxy-manno-octulosonate cytidylyltransferase (CMP-KDO synthetase)	G332	K01627	0.0725	K07737	-0.0204
K00982	glnE	glutamate-ammonia-ligase adenylyltransferase	G90	K07090	0.0349	K04079	-0.0271
K00983	E2.7.7.43, neuA, CMAS	N-acylneuraminate cytidylyltransferase	G334	K01654	0.2825	K01611	-0.0597
K00989	rph	ribonuclease PH	G43	K07148	0.0436	K03502	-0.0351
K00990	glnD	[protein-PII] uridylyltransferase	G305	K02517	0.0389	K03547	-0.0297
K00991	ispD	2-C-methyl-D-erythritol 4-phosphate cytidylyltransferase	G337	K01770	0.1546	K12506	-0.1255
K00997	E2.7.8.7, acpS	holo-[acyl-carrier protein] synthase	G204	K01489	0.0622	K00845	-0.0721
K00998	E2.7.8.8, pssA	phosphatidylserine synthase	G339	K01613	0.1730	K07636	-0.0420
K01006	ppdK	pyruvate,orthophosphate dikinase	G340	K09773	0.0599	K01907	-0.0483
K01007	E2.7.9.2, ppsA	pyruvate, water dikinase	G24	K07182	0.0436	K01572	-0.0483
K01008	E2.7.9.3, selD	selenide, water dikinase	G342	K03833	0.1637	K06904	-0.0458
K01010	E2.8.1.1, sseA	thiosulfate sulfurtransferase	G200	K03758	0.0625	K03386	-0.0623
K01011	E2.8.1.2	3-mercaptopyruvate sulfurtransferase	G344	K07245	0.0636	K01010	-0.0533
K01012	E2.8.1.6, bioB	biotin synthetase	G215	K01935	0.1521	K03523	-0.0493
K01028	E2.8.3.5A, scoA	3-oxoacid CoA-transferase subunit A	G315	K01029	0.2349	K01907	-0.0653
K01029	E2.8.3.5B, scoB	3-oxoacid CoA-transferase subunit B	G315	K01028	0.2349	K01907	-0.0660
K01042	selA	L-seryl-tRNA(Ser) seleniumtransferase	G342	K03833	0.2505	K07093	-0.0478
K01046	E3.1.1.3	triacylglycerol lipase	G146	K00754	0.0805	K12574	-0.0643
K01048	E3.1.1.5, pldB	lysophospholipase	G146	K01046	0.0667	K02919	-0.0549
K01053	E3.1.1.17	gluconolactonase	G351	K05396	0.0706	K07491	-0.0652
K01055	E3.1.1.24, pcaD	3-oxoadipate enol-lactonase	G9	K00019	0.0630	K09931	-0.0537
K01057	PGLS, pgl, devB	6-phosphogluconolactonase	G19	K00036	0.1356	K08369	-0.0439
K01058	E3.1.1.32, pldA	phospholipase A1	G354	K07798	0.0501	K09939	-0.0585
K01061	E3.1.1.45	carboxymethylenebutenolidase	G42	K00111	0.0449	K07089	-0.0450

KEGG identifier (gene *i*)	Name	Description	Correlog group	KEGG identifier (gene *j*)	w_{ij}	KEGG identifier (gene *j'*)	$w_{ij'}$
K01069	E3.1.2.6, gloB	hydroxyacylglutathione hydrolase	G268	K00821	0.0607	K06910	-0.0495
K01077	E3.1.3.1, phoA, phoB	alkaline phosphatase	G357	K03449	0.0577	K06223	-0.0641
K01079	E3.1.3.3, serB	phosphoserine phosphatase	G358	K07166	0.0695	K00364	-0.0636
K01081	E3.1.3.5	5'-nucleotidase	G184	K00558	0.0644	K11717	-0.0630
K01087	E3.1.3.12, otsB	trehalose-phosphatase	G231	K00697	0.1895	K09930	-0.0459
K01089	hisB	imidazoleglycerol-phosphate dehydratase	G361	K11755	0.0570	K01693	-0.0862
K01090	E3.1.3.16	protein phosphatase	G327	K01840	0.0708	K00756	-0.0590
K01091	E3.1.3.18, gph	phosphoglycolate phosphatase	G162	K01633	0.0678	K01118	-0.0624
K01092	E3.1.3.25, IMPA, suhB	myo-inositol-1(or 4)-monophosphatase	G364	K01724	0.0691	K01950	-0.0562
K01095	pgpA	phosphatidylglycerophosphatase A	G30	K07118	0.0409	K04755	-0.0467
K01104	E3.1.3.48	protein-tyrosine phosphatase	G366	K06188	0.0605	K03799	-0.0547
K01114	plcC	phospholipase C	G200	K00926	0.0702	K07011	-0.0632
K01118	acpD	FMN-dependent NADH-azoreductase	G368	K06904	0.0667	K01091	-0.0624
K01119	cpdB	2',3'-cyclic-nucleotide 2'-phosphodiesterase	G369	K02000	0.0814	K01788	-0.0667
K01126	E3.1.4.46, glpQ	glycerophosphoryl diester phosphodiesterase	G183	K01854	0.0643	K03695	-0.0571
K01129	dgt	dGTPase	G163	K00449	0.0638	K06148	-0.0486
K01139	spoT	guanosine-3',5'-bis(diphosphate) 3'-pyrophosphohydrolase	G372	K01618	0.0829	K00951	-0.0800
K01141	E3.1.11.1, sbcB	exodeoxyribonuclease I	G373	K03925	0.0418	K10026	-0.0344
K01142	E3.1.11.2, xthA	exodeoxyribonuclease III	G204	K00997	0.0594	K01151	-0.0618
K01147	rnb	exoribonuclease II	G89	K00259	0.0368	K03629	-0.0396
K01151	E3.1.21.2, nfo	deoxyribonuclease IV	G55	K03827	0.0462	K01142	-0.0618
K01153	hsdR	type I restriction enzyme, R subunit	G15	K03427	0.3793	K01934	-0.0443
K01154	hsdS	type I restriction enzyme, S subunit	G15	K01153	0.2989	K07154	-0.0730
K01159	ruvC	crossover junction endodeoxyribonuclease RuvC	G379	K03613	0.0843	K03571	-0.0870
K01175	E3.1.-.-		G37	K00257	0.0600	K01338	-0.0744
K01176	E3.2.1.1, amyA, malS	alpha-amylase	G381	K10112	0.0660	K05820	-0.0560
K01179	E3.2.1.4	endoglucanase	G230	K00694	0.1199	K03091	-0.0637
K01187	E3.2.1.20, malZ	alpha-glucosidase	G368	K03316	0.0887	K01273	-0.0691
K01188	E3.2.1.21	beta-glucosidase	G179	K00547	0.0814	K00599	-0.0729
K01190	lacZ	beta-galactosidase	G30	K12308	0.0893	K08987	-0.0753

KEGG identifier (gene *i*)	Name	Description	Correlog group	KEGG identifier (gene *j*)	w_{ij}	KEGG identifier (gene *j'*)	$w_{ij'}$
K01193	E3.2.1.26, sacA	beta-fructofuranosidase	G138	K02809	0.1118	K01531	-0.0636
K01207	E3.2.1.52, nagZ	beta-N-acetylhexosaminidase	G50	K00135	0.0858	K00754	-0.0608
K01222	E3.2.1.86A, celF	6-phospho-beta-glucosidase	G388	K03478	0.1055	K00868	-0.0557
K01226	E3.2.1.93, treC	trehalose-6-phosphate hydrolase	G138	K01193	0.0867	K03496	-0.0825
K01236	E3.2.1.141	maltooligosyltrehalose trehalohydrolase	G39	K06044	0.1930	K10543	-0.0578
K01239	E3.2.2.1, iunH	purine nucleosidase	G249	K00852	0.0944	K00432	-0.0685
K01241	amn	AMP nucleosidase	G161	K03773	0.0384	K00219	-0.0402
K01243	mtnN, mtn, pfs	S-adenosylhomocysteine/5'-methylthioadenosine nucleosidase	G393	K07173	0.0621	K01251	-0.0683
K01246	E3.2.2.20, tag	DNA-3-methyladenine glycosylase I	G381	K02824	0.0549	K00756	-0.0529
K01247	alkA	DNA-3-methyladenine glycosylase II	G395	K10778	0.1036	K03707	-0.0575
K01251	E3.3.1.1, ahcY	adenosylhomocysteinase	G396	K06169	0.0403	K07173	-0.0756
K01255	CARP, pepA	leucyl aminopeptidase	G397	K05592	0.0450	K00796	-0.0622
K01256	pepN	aminopeptidase N	G398	K01644	0.0450	K00721	-0.0380
K01258	pepT	tripeptide aminopeptidase	G281	K00965	0.0537	K00782	-0.0479
K01259	E3.4.11.5, pip	proline iminopeptidase	G146	K01423	0.0728	K07749	-0.0662
K01262	E3.4.11.9, pepP	X-Pro aminopeptidase	G90	K05569	0.0795	K01271	-0.0951
K01269	E3.4.11.-	aminopeptidase	G24	K03409	0.0772	K07506	-0.0666
K01270	E3.4.13.3, pepD	aminoacylhistidine dipeptidase	G90	K01262	0.0581	K07126	-0.0533
K01271	E3.4.13.9, pepQ	X-Pro dipeptidase	G39	K00102	0.0751	K01262	-0.0951
K01273	E3.4.13.19, DPEP1	membrane dipeptidase	G17	K00459	0.0780	K01187	-0.0691
K01284	dcp	peptidyl-dipeptidase Dcp	G406	K05800	0.0660	K01426	-0.0520
K01286	E3.4.16.4	D-alanyl-D-alanine carboxypeptidase	G184	K07458	0.0767	K09803	-0.0560
K01295	E3.4.17.11	glutamate carboxypeptidase	G21	K00040	0.0661	K08641	-0.0795
K01297	ldcA	muramoyltetrapeptide carboxypeptidase	G409	K07115	0.0841	K06223	-0.0810
K01299	E3.4.17.19	carboxypeptidase Taq	G149	K07323	0.0712	K01552	-0.0625
K01304	E3.4.19.3, pcp	pyroglutamyl-peptidase	G411	K06956	0.0565	K02348	-0.0578
K01322	E3.4.21.26, PREP	prolyl oligopeptidase	G90	K05570	0.0622	K07319	-0.0589
K01338	E3.4.21.53, lon	ATP-dependent Lon protease	G413	K03499	0.0499	K01175	-0.0744
K01354	ptrB	oligopeptidase B	G414	K05794	0.0613	K11741	-0.0493
K01356	lexA	repressor LexA	G13	K07507	0.0495	K01119	-0.0436
K01362	E3.4.21.-		G312	K00936	0.0869	K00758	-0.0621

KEGG identifier (gene *i*)	Name	Description	Correlog group	KEGG identifier (gene *j*)	w_{ij}	KEGG identifier (gene *j'*)	$w_{ij'}$
K01414	prlC	oligopeptidase A	G219	K00660	0.0467	K08602	-0.0381
K01417	E3.4.24.-		G71	K00215	0.0737	K11749	-0.0922
K01419	hslV, clpQ	ATP-dependent HslUV protease, peptidase subunit HslV	G419	K03667	0.1731	K00757	-0.0432
K01420	fnr	CRP/FNR family transcriptional regulator, anaerobic regulatory	G316	K07075	0.0536	K02027	-0.0506
K01421	yhgE	putative membrane protein	G249	K00852	0.0542	K09165	-0.0507
K01423	E3.4.-.-		G146	K00754	0.1360	K06202	-0.0645
K01424	E3.5.1.1, ansA, ansB	L-asparaginase	G266	K03892	0.0533	K01753	-0.0462
K01425	E3.5.1.2, glsA	glutaminase	G37	K01436	0.0626	K02004	-0.0511
K01426	E3.5.1.4, amiE	amidase	G425	K01532	0.0641	K07218	-0.0559
K01428	ureC	urease alpha subunit	G426	K01429	0.1615	K01551	-0.0185
K01429	ureB	urease beta subunit	G426	K01430	0.1637	K06978	-0.0226
K01430	ureA	urease gamma subunit	G426	K01429	0.1637	K06978	-0.0257
K01433	purU	formyltetrahydrofolate deformylase	G301	K11105	0.0719	K01938	-0.0569
K01436	E3.5.1.14	aminoacylase	G37	K00100	0.0801	K06904	-0.0693
K01438	E3.5.1.16, argE	acetylornithine deacetylase	G184	K07458	0.0554	K00257	-0.0529
K01439	dapE	succinyl-diaminopimelate desuccinylase	G223	K00674	0.0881	K07461	-0.0579
K01440	E3.5.1.19	nicotinamidase	G244	K08281	0.2300	K06904	-0.0601
K01442	E3.5.1.24	choloylglycine hydrolase	G55	K03827	0.0841	K06929	-0.0628
K01443	E3.5.1.25, nagA, AMDHD2	N-acetylglucosamine-6-phosphate deacetylase	G435	K02564	0.0915	K00754	-0.0560
K01447	E3.5.1.28A, cwlA, xlyA, xlyB	N-acetylmuramoyl-L-alanine amidase	G414	K03574	0.0923	K04047	-0.0637
K01448	E3.5.1.28B, amiA, amiB, amiC	N-acetylmuramoyl-L-alanine amidase	G437	K09888	0.0553	K01772	-0.0451
K01451	E3.5.1.32, hipO	hippurate hydrolase	G219	K02074	0.0698	K06871	-0.0665
K01464	E3.5.2.2, DPYS	dihydropyrimidinase	G196	K00598	0.0908	K00878	-0.0561
K01467	E3.5.2.6, ampC, penP	beta-lactamase	G146	K01046	0.0671	K07054	-0.0598
K01468	E3.5.2.7, hutI	imidazolonepropionase	G441	K01712	0.2028	K07473	-0.0383
K01473	hyuA	N-methylhydantoinase A	G442	K01474	0.3521	K07483	-0.0462
K01474	hyuB	N-methylhydantoinase B	G442	K01473	0.3521	K07028	-0.0519
K01476	E3.5.3.1, rocF, arg	arginase	G444	K04750	0.0665	K07004	-0.0549
K01478	E3.5.3.6, arcA	arginine deiminase	G200	K00926	0.2087	K12942	-0.0619

KEGG identifier (gene *i*)	Name	Description	Correlog group	KEGG identifier (gene *j*)	w_{ij}	KEGG identifier (gene *j'*)	$w_{ij'}$
K01479	E3.5.3.8, hutG	formiminoglutamase	G441	K01468	0.1174	K07404	-0.0609
K01480	E3.5.3.11, speB	agmatinase	G89	K00259	0.0547	K10536	-0.0880
K01484	astB	succinylarginine dihydrolase	G222	K00673	0.1168	K01442	-0.0437
K01485	E3.5.4.1, codA	cytosine deaminase	G230	K01179	0.1052	K01262	-0.0834
K01486	ade	adenine deaminase	G196	K01464	0.0744	K06978	-0.0473
K01487	E3.5.4.3, guaD	guanine deaminase	G34	K00087	0.0944	K07238	-0.0641
K01488	E3.5.4.4, ADA, add	adenosine deaminase	G190	K07778	0.0646	K07483	-0.0588
K01489	E3.5.4.5, cdd	cytidine deaminase	G204	K00625	0.0833	K00926	-0.0527
K01493	comEB	dCMP deaminase	G161	K02919	0.0578	K01494	-0.1124
K01494	E3.5.4.13, dcd	dCTP deaminase	G455	K04477	0.0409	K01493	-0.1124
K01495	E3.5.4.16, folE	GTP cyclohydrolase I	G162	K00796	0.0876	K09007	-0.0872
K01496	hisI	phosphoribosyl-AMP cyclohydrolase	G457	K01523	0.1251	K11755	-0.1079
K01497	E3.5.4.25, ribA	GTP cyclohydrolase II	G458	K02858	0.1775	K03496	-0.0436
K01501	E3.5.5.1	nitrilase	G459	K02650	0.0645	K03098	-0.0571
K01512	E3.6.1.7, acyP	acylphosphatase	G460	K03092	0.0722	K03606	-0.0617
K01514	E3.6.1.11, ppx	exopolyphosphatase	G146	K00837	0.0868	K01623	-0.0576
K01515	E3.6.1.13	ADP-ribose pyrophosphatase	G90	K02238	0.0473	K07213	-0.0490
K01520	E3.6.1.23, dut	dUTP pyrophosphatase	G90	K01262	0.0631	K03320	-0.0688
K01523	hisE	phosphoribosyl-ATP pyrophosphohydrolase	G457	K01496	0.1251	K11755	-0.0852
K01524	gppA	guanosine-5'-triphosphate,3'-diphosphate pyrophosphatase	G381	K01176	0.0629	K03297	-0.0745
K01525	apaH	bis(5'-nucleosyl)-tetraphosphatase (symmetrical)	G301	K11105	0.0302	K03976	-0.0262
K01529	E3.6.1.-		G190	K00952	0.1021	K01739	-0.0697
K01531	E3.6.3.2, mgtA, mgtB	Mg2+-importing ATPase	G468	K06925	0.0852	K01193	-0.0636
K01532	E3.6.3.3, zntA	Cd2+-exporting ATPase	G425	K11065	0.0807	K09946	-0.0630
K01533	E3.6.3.4, ATP7, copA	Cu2+-exporting ATPase	G470	K06213	0.0531	K03321	-0.0662
K01537	E3.6.3.8	Ca2+-transporting ATPase	G471	K03453	0.0762	K00837	-0.0669
K01546	kdpA	K+-transporting ATPase ATPase A chain	G472	K01547	0.2106	K06917	-0.0280
K01547	kdpB	K+-transporting ATPase ATPase B chain	G472	K01548	0.2134	K03672	-0.0308

KEGG identifier (gene i)	Name	Description	Correlog group	KEGG identifier (gene j)	w_{ij}	KEGG identifier (gene j')	$w_{ij'}$
K01548	kdpC	K+-transporting ATPase ATPase C chain	G472	K01547	0.2134	K00065	-0.0395
K01551	E3.6.3.16, arsA	arsenite-transporting ATPase	G475	K02230	0.0761	K06904	-0.0673
K01552	E3.6.3.-		G476	K07028	0.0906	K07054	-0.0709
K01555	E3.7.1.2, FAH	fumarylacetoacetase	G165	K00451	0.1021	K01442	-0.0561
K01556	E3.7.1.3	kynureninase	G93	K00453	0.1717	K02433	-0.0554
K01560	E3.8.1.2	2-haloacid dehalogenase	G10	K00020	0.0636	K03574	-0.0583
K01571	E4.1.1.3A, oadA	oxaloacetate decarboxylase, alpha subunit	G480	K01572	0.1605	K01960	-0.0583
K01572	E4.1.1.3B, oadB	oxaloacetate decarboxylase, beta subunit	G480	K01571	0.1605	K02919	-0.0561
K01575	E4.1.1.5, alsD	acetolactate decarboxylase	G138	K01193	0.0784	K01607	-0.0616
K01579	panD	aspartate 1-decarboxylase	G199	K01918	0.0813	K09928	-0.0492
K01580	E4.1.1.15, gadB	glutamate decarboxylase	G200	K01114	0.0674	K01786	-0.0561
K01581	E4.1.1.17, ODC1, speC, speF	ornithine decarboxylase	G37	K00100	0.0483	K04561	-0.0483
K01585	E4.1.1.19S, speA	arginine decarboxylase	G486	K12251	0.0434	K04750	-0.0471
K01586	lysA	diaminopimelate decarboxylase	G487	K01755	0.0808	K03569	-0.0579
K01589	purK	5-(carboxyamino)imidazole ribonucleotide synthase	G488	K11175	0.0592	K00860	-0.0421
K01595	E4.1.1.31, ppc	phosphoenolpyruvate carboxylase	G489	K07034	0.0495	K03761	-0.0623
K01596	E4.1.1.32, pckA, PEPCK	phosphoenolpyruvate carboxykinase (GTP)	G490	K06988	0.0390	K01610	-0.0635
K01599	E4.1.1.37, hemE	uroporphyrinogen decarboxylase	G75	K01772	0.0730	K01950	-0.0363
K01601	rbcL	ribulose-bisphosphate carboxylase large chain	G286	K00855	0.1118	K05520	-0.0516
K01607	E4.1.1.44, pcaC	4-carboxymuconolactone decarboxylase	G9	K01055	0.0580	K01575	-0.0616
K01609	E4.1.1.48, trpC	indole-3-glycerol phosphate synthase	G246	K00766	0.1446	K00832	-0.0374
K01610	E4.1.1.49, pckA	phosphoenolpyruvate carboxykinase (ATP)	G486	K12251	0.0509	K01596	-0.0635
K01611	E4.1.1.50, speD	S-adenosylmethionine decarboxylase	G259	K00797	0.1701	K00983	-0.0597
K01613	E4.1.1.65, psd	phosphatidylserine decarboxylase	G339	K00998	0.1730	K03784	-0.0433
K01618	E4.1.1.-		G372	K01139	0.0829	K04565	-0.0571
K01619	E4.1.2.4, deoC	deoxyribose-phosphate aldolase	G238	K00756	0.0689	K01262	-0.0447
K01620	E4.1.2.5, ltaA	threonine aldolase	G500	K07461	0.0611	K07300	-0.0574
K01623	ALDO, fbaB	fructose-bisphosphate aldolase, class I	G16	K07112	0.0703	K01624	-0.0863

KEGG identifier (gene *i*)	Name	Description	Correlog group	KEGG identifier (gene *j*)	w_{ij}	KEGG identifier (gene *j'*)	$w_{ij'}$
K01624	FBA, fbaA	fructose-bisphosphate aldolase, class II	G282	K00850	0.0643	K01623	-0.0863
K01625	eda	2-dehydro-3-deoxyphosphogluconate aldolase	G295	K00874	0.1059	K01779	-0.0598
K01626	E2.5.1.54, aroF, aroG, aroH	3-deoxy-7-phosphoheptulonate synthase	G302	K01735	0.0360	K03856	-0.0783
K01627	kdsA	2-dehydro-3-deoxyphosphooctonate aldolase (KDO 8-P synthase)	G332	K06041	0.0858	K07737	-0.0262
K01628	fucA	L-fuculose-phosphate aldolase	G489	K07034	0.0694	K02022	-0.0594
K01633	E4.1.2.25, folB	dihydroneopterin aldolase	G162	K00950	0.1053	K00215	-0.0490
K01637	E4.1.3.1, aceA	isocitrate lyase	G508	K01638	0.1502	K01795	-0.0435
K01638	E2.3.3.9, aceB, glcB	malate synthase	G508	K01637	0.1502	K03284	-0.0370
K01640	E4.1.3.4, hmgL	hydroxymethylglutaryl-CoA lyase	G86	K01966	0.0592	K07114	-0.0520
K01644	citE	citrate lyase subunit beta	G398	K05966	0.1713	K01934	-0.0474
K01647	CS, gltA	citrate synthase	G18	K00031	0.1103	K06013	-0.0471
K01649	E2.3.3.13, leuA	2-isopropylmalate synthase	G25	K01704	0.0810	K03892	-0.0330
K01652	E2.2.1.6L, ilvB, ilvG, ilvI	acetolactate synthase I/II/III large subunit	G26	K01687	0.0899	K01488	-0.0472
K01653	E2.2.1.6S, ilvH, ilvN	acetolactate synthase I/III small subunit	G26	K00053	0.0619	K03406	-0.0411
K01654	E2.5.1.56, neuB	N-acetylneuraminate synthase	G334	K00983	0.2825	K01436	-0.0556
K01655	E2.3.3.14	homocitrate synthase	G517	K02594	0.2488	K09118	-0.0344
K01657	E4.1.3.27A, trpE	anthranilate synthase component I	G518	K01658	0.0822	K00788	-0.0365
K01658	E4.1.3.27B, trpD	anthranilate synthase component II	G518	K01657	0.0822	K01664	-0.0534
K01659	E2.3.3.5, prpC	2-methylcitrate synthase	G520	K01720	0.1348	K00966	-0.0623
K01661	menB	naphthoate synthase	G521	K01911	0.1255	K03179	-0.0401
K01662	dxs	1-deoxy-D-xylulose-5-phosphate synthase	G36	K00099	0.1110	K01091	-0.0524
K01664	pabA	para-aminobenzoate synthetase component II	G523	K01665	0.0926	K00262	-0.0637
K01665	pabB	para-aminobenzoate synthetase component I	G523	K02619	0.1237	K03312	-0.0534
K01666	E4.1.3.39, mhpE	4-hydroxy 2-oxovalerate aldolase	G525	K01725	0.0656	K11177	-0.0662
K01669	E4.1.99.3, phrB	deoxyribodipyrimidine photo-lyase	G526	K02068	0.0639	K07567	-0.0555
K01673	E4.2.1.1A, cynT	carbonic anhydrase	G527	K07053	0.0417	K05595	-0.0425
K01674	E4.2.1.1B, cah	carbonic anhydrase	G396	K03534	0.0738	K06076	-0.0654

KEGG identifier (gene *i*)	Name	Description	Correlog group	KEGG identifier (gene *j*)	w_{ij}	KEGG identifier (gene *j'*)	$w_{ij'}$
K01676	E4.2.1.2A, fumA, fumB	fumarate hydratase, class I	G529	K06013	0.0532	K01552	-0.0461
K01679	E4.2.1.2B, fumC	fumarate hydratase, class II	G18	K01681	0.0579	K03305	-0.0552
K01681	ACO, acnA	aconitate hydratase 1	G18	K01647	0.0734	K01682	-0.0513
K01682	acnB	aconitate hydratase 2	G532	K07124	0.0423	K01681	-0.0513
K01685	uxaA	altronate hydrolase	G5	K01753	0.0606	K07106	-0.0551
K01686	uxuA	mannonate dehydratase	G21	K01812	0.1533	K05521	-0.0615
K01687	E4.2.1.9, ilvD	dihydroxy-acid dehydratase	G26	K01652	0.0899	K00067	-0.0303
K01690	edd	phosphogluconate dehydratase	G295	K01625	0.1022	K07182	-0.0411
K01692	E4.2.1.17, paaG	enoyl-CoA hydratase	G11	K00022	0.1122	K00389	-0.0493
K01693	E4.2.1.19, hisB	imidazoleglycerol-phosphate dehydratase	G6	K00013	0.0580	K01089	-0.0862
K01695	E4.2.1.20A, trpA	tryptophan synthase alpha chain	G273	K01696	0.2059	K00813	-0.0735
K01696	E4.2.1.20B, trpB	tryptophan synthase beta chain	G273	K01695	0.2059	K00813	-0.0670
K01697	E4.2.1.22, CBS	cystathionine beta-synthase	G107	K06999	0.0631	K00042	-0.0539
K01698	E4.2.1.24, hemB	porphobilinogen synthase	G542	K01749	0.1149	K01618	-0.0314
K01703	leuC	3-isopropylmalate/(R)-2-methylmalate dehydratase large subunit	G25	K01704	0.1315	K01914	-0.0361
K01704	leuD	3-isopropylmalate/(R)-2-methylmalate dehydratase small subunit	G25	K01703	0.1315	K03317	-0.0281
K01706	E4.2.1.40, gudD	glucarate dehydratase	G22	K00661	0.0675	K06867	-0.0798
K01709	rfbG	CDP-glucose 4,6-dehydratase	G173	K00978	0.3982	K10680	-0.0608
K01710	E4.2.1.46, rfbB	dTDP-glucose 4,6-dehydratase	G202	K00973	0.1871	K09985	-0.0684
K01711	E4.2.1.47, gmd	GDPmannose 4,6-dehydratase	G548	K02377	0.2439	K07127	-0.0570
K01712	E4.2.1.49, hutU	urocanate hydratase	G441	K01468	0.2028	K09157	-0.0366
K01716	fabA	3-hydroxydecanoyl-[acyl-carrier-protein] dehydratase	G213	K00647	0.0529	K03811	-0.0338
K01719	E4.2.1.75, hemD	uroporphyrinogen-III synthase	G542	K01749	0.1045	K02919	-0.0504
K01720	E4.2.1.79, prpD	2-methylcitrate dehydratase	G520	K03417	0.1456	K03672	-0.0591
K01724	E4.2.1.96, PCBD, phhB	4a-hydroxytetrahydrobiopterin dehydratase	G364	K03821	0.0720	K00287	-0.0548
K01725	E4.2.1.104, cynS	cyanate lyase	G525	K09958	0.0889	K01273	-0.0602

KEGG identifier (gene *i*)	Name	Description	Correlog group	KEGG identifier (gene *j*)	w_{ij}	KEGG identifier (gene *j'*)	$w_{ij'}$
K01733	E4.2.3.1, thrC	threonine synthase	G1	K00003	0.1681	K03758	-0.0562
K01734	E4.2.3.3, mgsA	methylglyoxal synthase	G556	K09967	0.0531	K03503	-0.0611
K01735	E4.2.3.4, ARO1	3-dehydroquinate synthase	G302	K00891	0.1284	K02652	-0.0399
K01736	E4.2.3.5, aroC	chorismate synthase	G7	K00014	0.0923	K07727	-0.0371
K01737	E4.2.3.12, ptpS	6-pyruvoyl tetrahydrobiopterin synthase	G559	K06920	0.0991	K03800	-0.0465
K01738	cysK	cysteine synthase A	G210	K00640	0.0992	K07082	-0.0471
K01739	E2.5.1.48, metB	cystathionine gamma-synthase	G162	K00951	0.0511	K01529	-0.0697
K01740	E2.5.1.49, metY	O-acetylhomoserine (thiol)-lyase	G211	K00641	0.0859	K03927	-0.0596
K01744	aspA	aspartate ammonia-lyase	G54	K02509	0.0618	K03518	-0.0526
K01745	E4.3.1.3, hutH	histidine ammonia-lyase	G441	K01712	0.1784	K07145	-0.0412
K01749	E2.5.1.61, hemC	hydroxymethylbilane synthase	G542	K01698	0.1149	K01256	-0.0288
K01750	E4.3.1.12, ocd	ornithine cyclodeaminase	G532	K01761	0.0550	K05350	-0.0671
K01752	E4.3.1.17, sdaA	L-serine dehydratase	G251	K00782	0.0528	K01426	-0.0497
K01753	dsdA	D-serine dehydratase	G5	K07395	0.0664	K07322	-0.0691
K01754	E4.3.1.19, ilvA, tdcB	threonine dehydratase	G26	K01687	0.0756	K02221	-0.0471
K01755	E4.3.2.1, argH	argininosuccinate lyase	G487	K01940	0.2321	K05540	-0.0385
K01759	E4.4.1.5, GLO1, gloA	lactoylglutathione lyase	G268	K01069	0.0552	K03741	-0.0501
K01760	E4.4.1.8, metC	cystathionine beta-lyase	G223	K01778	0.0710	K07778	-0.0557
K01761	E4.4.1.11, mdeA	methionine-gamma-lyase	G532	K07334	0.0625	K10543	-0.0676
K01768	E4.6.1.1	adenylate cyclase	G89	K03287	0.0845	K03478	-0.0574
K01770	ispF	2-C-methyl-D-erythritol 2,4-cyclodiphosphate synthase	G337	K00991	0.1546	K12506	-0.1349
K01772	E4.99.1.1, hemH	ferrochelatase	G75	K01599	0.0730	K09793	-0.0493
K01776	E5.1.1.3, murI	glutamate racemase	G292	K13038	0.0773	K11085	-0.0496
K01778	dapF	diaminopimelate epimerase	G223	K00674	0.0952	K06153	-0.0525
K01779	E5.1.1.13	aspartate racemase	G179	K01188	0.0617	K03761	-0.0658
K01782	E5.1.2.3	3-hydroxybutyryl-CoA epimerase	G11	K00022	0.0719	K06988	-0.0465
K01784	galE, GALE	UDP-glucose 4-epimerase	G89	K02823	0.0682	K03295	-0.0528
K01785	E5.1.3.3, galM	aldose 1-epimerase	G281	K00849	0.0751	K02478	-0.0597
K01786	araD	L-ribulose-5-phosphate 4-epimerase	G373	K01804	0.1690	K01580	-0.0561

KEGG identifier (gene *i*)	Name	Description	Correlog group	KEGG identifier (gene *j*)	w_{ij}	KEGG identifier (gene *j'*)	$w_{ij'}$
K01788	nanE	N-acylglucosamine-6-phosphate 2-epimerase	G468	K07101	0.0959	K01119	-0.0667
K01790	rfbC	dTDP-4-dehydrorhamnose 3,5-epimerase	G31	K00067	0.2417	K01190	-0.0670
K01791	wecB	UDP-N-acetylglucosamine 2-epimerase	G43	K02472	0.1095	K00856	-0.0581
K01795	E5.1.3.-		G187	K05993	0.0813	K07126	-0.0664
K01800	E5.2.1.2, maiA	maleylacetoacetate isomerase	G165	K00451	0.0603	K09946	-0.0435
K01802	E5.2.1.8	peptidylprolyl isomerase	G589	K07102	0.0693	K01262	-0.0935
K01804	araA	L-arabinose isomerase	G373	K01786	0.1690	K11209	-0.0493
K01805	xylA	xylose isomerase	G285	K00854	0.1556	K01761	-0.0556
K01807	E5.3.1.6A, rpiA	ribose 5-phosphate isomerase A	G19	K00036	0.0370	K01808	-0.0716
K01808	E5.3.1.6B, rpiB	ribose 5-phosphate isomerase B	G50	K00135	0.0809	K01807	-0.0716
K01809	E5.3.1.8, manA	mannose-6-phosphate isomerase	G327	K00971	0.0689	K00459	-0.0467
K01812	uxaC	glucuronate isomerase	G21	K01686	0.1533	K00983	-0.0531
K01814	E5.3.1.16, hisA	phosphoribosylformimino-5-aminoimidazole carboxamide ribotide	G6	K02500	0.1023	K05350	-0.0332
K01816	E5.3.1.22, gip	hydroxypyruvate isomerase	G468	K03307	0.0576	K07814	-0.0580
K01817	E5.3.1.24, trpF	phosphoribosylanthranilate isomerase	G273	K01695	0.1172	K07037	-0.0605
K01821	E5.3.2.-	4-oxalocrotonate tautomerase	G251	K00782	0.0533	K00886	-0.0485
K01823	E5.3.3.2, idi	isopentenyl-diphosphate delta-isomerase	G468	K07101	0.0723	K03527	-0.0547
K01825	E5.3.3.8, DCI	dodecenoyl-CoA delta-isomerase	G11	K01782	0.0637	K05596	-0.0483
K01826	E5.3.3.10, hpaF	5-carboxymethyl-2-hydroxymuconate isomerase	G54	K00151	0.1336	K00121	-0.0632
K01835	E5.4.2.2, pgm	phosphoglucomutase	G603	K01897	0.0727	K09922	-0.0459
K01839	deoB	phosphopentomutase	G604	K03784	0.0795	K06885	-0.0578
K01840	E5.4.2.8, manB	phosphomannomutase	G327	K09810	0.0870	K02003	-0.0627
K01843	E5.4.3.2, kamA	lysine 2,3-aminomutase	G606	K04568	0.0816	K02010	-0.0521
K01845	E5.4.3.8, hemL	glutamate-1-semialdehyde 2,1-aminomutase	G542	K01698	0.1060	K03458	-0.0468
K01847	MUT	methylmalonyl-CoA mutase	G608	K07588	0.1139	K02347	-0.0451
K01854	E5.4.99.9, glf	UDP-galactopyranose mutase	G183	K00557	0.0670	K00836	-0.0621
K01857	E5.5.1.2, pcaB	3-carboxy-cis,cis-muconate cycloisomerase	G163	K00448	0.1559	K00065	-0.0404
K01858	E5.5.1.4, INO1	myo-inositol-1-phosphate synthase	G468	K06925	0.0498	K00147	-0.0500
K01878	glyQ	glycyl-tRNA synthetase alpha chain	G612	K01879	0.1357	K01880	-0.1229

KEGG identifier (gene *i*)	Name	Description	Correlog group	KEGG identifier (gene *j*)	w_{ij}	KEGG identifier (gene *j'*)	$w_{ij'}$
K01879	glyS	glycyl-tRNA synthetase beta chain	G612	K01878	0.1357	K01880	-0.1202
K01880	GARS, glyS1	glycyl-tRNA synthetase	G508	K02553	0.0411	K01878	-0.1229
K01886	QARS, glnS	glutaminyl-tRNA synthetase	G615	K03698	0.0527	K02434	-0.0417
K01893	NARS, asnS	asparaginyl-tRNA synthetase	G616	K01914	0.0390	K07039	-0.0372
K01894	gluQ	glutamyl-Q tRNA(Asp) synthetase	G617	K07712	0.0383	K01581	-0.0326
K01895	ACSS, acs	acetyl-CoA synthetase	G16	K01623	0.0386	K01779	-0.0435
K01897	ACSL, fadD	long-chain acyl-CoA synthetase	G603	K01835	0.0727	K01980	-0.0464
K01902	sucD	succinyl-CoA synthetase alpha subunit	G620	K01903	0.2217	K03312	-0.0449
K01903	sucC	succinyl-CoA synthetase beta subunit	G620	K01902	0.2217	K00567	-0.0417
K01907	AACS, acsA	acetoacetyl-CoA synthetase	G532	K01761	0.0545	K01029	-0.0660
K01908	E6.2.1.17, prpE	propionyl-CoA synthetase	G520	K01720	0.0543	K08974	-0.0461
K01909	E6.2.1.20, aas	long-chain-fatty-acid--[acyl-carrier-protein] ligase	G624	K05939	0.2418	K09117	-0.0381
K01911	menE	O-succinylbenzoic acid--CoA ligase	G521	K01661	0.1255	K03179	-0.0323
K01912	E6.2.1.30, paaK	phenylacetate-CoA ligase	G87	K04090	0.0595	K11747	-0.0545
K01914	asnA	aspartate--ammonia ligase	G616	K12267	0.0612	K02027	-0.0696
K01916	E6.3.1.5, nadE	NAD+ synthase	G13	K06929	0.0721	K01950	-0.2107
K01918	E6.3.2.1, panC	pantoate--beta-alanine ligase	G199	K00606	0.1778	K07021	-0.0465
K01919	gshA	glutamate--cysteine ligase	G73	K00917	0.0525	K06897	-0.0405
K01920	E6.3.2.3, gshB	glutathione synthase	G73	K01919	0.0399	K00102	-0.0318
K01926	rex	AT-rich DNA-binding protein	G490	K04063	0.0394	K07734	-0.0347
K01930	E6.3.2.17	folylpolyglutamate synthase	G162	K00950	0.0991	K11754	-0.1534
K01934	E6.3.3.2	5-formyltetrahydrofolate cyclo-ligase	G606	K01843	0.0656	K01262	-0.0867
K01935	bioD	dethiobiotin synthetase	G215	K00833	0.2343	K05515	-0.0409
K01938	E6.3.4.3, fhs	formate--tetrahydrofolate ligase	G636	K08156	0.0747	K01433	-0.0569
K01940	E6.3.4.5, argG	argininosuccinate synthase	G487	K01755	0.2321	K06158	-0.0366
K01946	E6.3.4.14, accC	biotin carboxylase	G638	K01961	0.1459	K02168	-0.0495
K01950	E6.3.5.1, NADSYN1, QNS1, nadE	NAD+ synthase (glutamine-hydrolysing)	G146	K00680	0.0527	K01916	-0.2107
K01953	E6.3.5.4, asnB	asparagine synthase (glutamine-hydrolysing)	G13	K07003	0.0624	K04767	-0.0590

KEGG identifier (gene *i*)	Name	Description	Correlog group	KEGG identifier (gene *j*)	w_{ij}	KEGG identifier (gene *j'*)	$w_{ij'}$
K01959	E6.4.1.1A, pycA	pyruvate carboxylase subunit A	G641	K01960	0.2688	K02188	-0.0483
K01960	E6.4.1.1B, pycB	pyruvate carboxylase subunit B	G641	K01959	0.2688	K01571	-0.0583
K01961	ACCC	acetyl-CoA carboxylase, biotin carboxylase subunit	G638	K01946	0.1459	K03699	-0.0495
K01962	ACCA	acetyl-CoA carboxylase carboxyl transferase subunit alpha	G644	K01963	0.1948	K01966	-0.0362
K01963	ACCD	acetyl-CoA carboxylase carboxyl transferase subunit beta	G644	K01962	0.1948	K01966	-0.0323
K01966	E6.4.1.3B, pccB	propionyl-CoA carboxylase beta chain	G86	K01640	0.0592	K06897	-0.0446
K01968	E6.4.1.4A	3-methylcrotonyl-CoA carboxylase alpha subunit	G468	K01823	0.0502	K00694	-0.0405
K01971	E6.5.1.1, lig	DNA ligase (ATP)	G648	K10979	0.1686	K03782	-0.0550
K01975	ligT	2'-5' RNA ligase	G649	K07403	0.0582	K03457	-0.0668
K01977	16SrRNA, rrs	16S ribosomal RNA	G650	K01980	0.3975	K00919	-0.0482
K01980	23SrRNA, rrl	23S ribosomal RNA	G650	K01977	0.3975	K00882	-0.0470
K01985	5SrRNA, rrf	5S ribosomal RNA	G650	K01977	0.3429	K02238	-0.0615
K01989	ABC.X4.S	putative ABC transport system substrate-binding protein	G653	K05833	0.2394	K07001	-0.0404
K01990	ABC-2.A	ABC-2 type transport system ATP-binding protein	G654	K01992	0.2332	K07341	-0.0649
K01991	ABC-2.OM, wza	polysaccharide export outer membrane protein	G532	K09803	0.0617	K06175	-0.0482
K01992	ABC-2.P	ABC-2 type transport system permease protein	G654	K01990	0.2332	K00375	-0.0581
K01993	ABC-2.TX	HlyD family secretion protein	G654	K01990	0.0840	K06147	-0.0547
K01995	livG	branched-chain amino acid transport system ATP-binding protein	G658	K01996	0.1736	K01478	-0.0290
K01996	livF	branched-chain amino acid transport system ATP-binding protein	G658	K01995	0.1736	K09698	-0.0338
K01997	livH	branched-chain amino acid transport system permease protein	G658	K01995	0.1717	K07038	-0.0300
K01998	livM	branched-chain amino acid transport system permease protein	G658	K01997	0.1652	K01487	-0.0352
K01999	livK	branched-chain amino acid transport system substrate-binding protein	G658	K01997	0.1442	K07263	-0.0390
K02000	proV	glycine betaine/proline transport system ATP-binding protein	G369	K02001	0.2678	K07148	-0.0524
K02001	proW	glycine betaine/proline transport system permease protein	G369	K02000	0.2678	K11209	-0.0594
K02002	proX	glycine betaine/proline transport system substrate-binding protein	G369	K02001	0.1247	K05782	-0.0515

KEGG identifier (gene *i*)	Name	Description	Correlog group	KEGG identifier (gene *j*)	w_{ij}	KEGG identifier (gene *j'*)	$w_{ij'}$
K02003	ABC.CD.A		G666	K02004	0.1282	K01950	-0.0710
K02004	ABC.CD.P		G666	K02003	0.1282	K02647	-0.0639
K02005	ABC.CD.TX	HlyD family secretion protein	G2	K00974	0.0706	K01607	-0.0573
K02006	cbiO	cobalt/nickel transport system ATP-binding protein	G669	K02008	0.1942	K01322	-0.0585
K02007	cbiM	cobalt/nickel transport system permease protein	G669	K02008	0.1002	K00854	-0.0489
K02008	cbiQ	cobalt/nickel transport system permease protein	G669	K02006	0.1942	K03555	-0.0445
K02010	ABC.FE.A	iron(III) transport system ATP-binding protein	G672	K02011	0.1254	K06137	-0.0703
K02011	ABC.FE.P	iron(III) transport system permease protein	G672	K02012	0.2789	K10041	-0.0550
K02012	ABC.FE.S	iron(III) transport system substrate-binding protein	G672	K02011	0.2789	K01207	-0.0407
K02013	ABC.FEV.A	iron complex transport system ATP-binding protein	G675	K02015	0.2417	K01295	-0.0405
K02014	ABC.FEV.OM	iron complex outermembrane recepter protein	G676	K03832	0.0481	K01740	-0.0349
K02015	ABC.FEV.P	iron complex transport system permease protein	G675	K02013	0.2417	K08641	-0.0394
K02016	ABC.FEV.S	iron complex transport system substrate-binding protein	G675	K02015	0.1648	K01179	-0.0523
K02017	modC	molybdate transport system ATP-binding protein	G679	K02020	0.0656	K01501	-0.0453
K02018	modB	molybdate transport system permease protein	G679	K02020	0.1780	K03409	-0.0430
K02019	modE	molybdate transport system regulatory protein	G33	K00077	0.0606	K07089	-0.0478
K02020	modA	molybdate transport system substrate-binding protein	G679	K02018	0.1780	K07665	-0.0370
K02022	ABC.MR.TX		G683	K07062	0.0560	K01628	-0.0594
K02023	ABC.MS.A	multiple sugar transport system ATP-binding protein	G684	K02027	0.1662	K01533	-0.0630
K02025	ABC.MS.P	multiple sugar transport system permease protein	G685	K02026	0.2666	K03712	-0.0513
K02026	ABC.MS.P1	multiple sugar transport system permease protein	G685	K02025	0.2666	K07667	-0.0425
K02027	ABC.MS.S	multiple sugar transport system substrate-binding protein	G684	K02023	0.1662	K01914	-0.0696
K02028	ABC.PA.A	polar amino acid transport system ATP-binding protein	G688	K02029	0.1814	K01551	-0.0535
K02029	ABC.PA.P	polar amino acid transport system permease protein	G688	K02030	0.2102	K07124	-0.0537
K02030	ABC.PA.S	polar amino acid transport system substrate-binding protein	G688	K02029	0.2102	K01273	-0.0625
K02031	ABC.PE.A	peptide/nickel transport system ATP-binding protein	G691	K02032	0.2142	K06890	-0.0426

KEGG identifier (gene *i*)	Name	Description	Correlog group	KEGG identifier (gene *j*)	w_{ij}	KEGG identifier (gene *j'*)	$w_{ij'}$
K02032	ABC.PE.A1	peptide/nickel transport system ATP-binding protein	G691	K02031	0.2142	K06148	-0.0732
K02033	ABC.PE.P	peptide/nickel transport system permease protein	G693	K02034	0.1955	K07778	-0.0474
K02034	ABC.PE.P1	peptide/nickel transport system permease protein	G693	K02033	0.1955	K01447	-0.0306
K02035	ABC.PE.S	peptide/nickel transport system substrate-binding protein	G693	K02033	0.1584	K02005	-0.0468
K02036	pstB	phosphate transport system ATP-binding protein	G696	K02040	0.1762	K00571	-0.0381
K02037	pstC	phosphate transport system permease protein	G697	K02038	0.2651	K06941	-0.0375
K02038	pstA	phosphate transport system permease protein	G697	K02037	0.2651	K08987	-0.0357
K02039	phoU	phosphate transport system protein	G696	K02036	0.1088	K02822	-0.0519
K02040	pstS	phosphate transport system substrate-binding protein	G696	K02036	0.1762	K03602	-0.0743
K02041	phnC	phosphonate transport system ATP-binding protein	G701	K02042	0.3093	K08234	-0.0438
K02042	phnE	phosphonate transport system permease protein	G701	K02041	0.3093	K03435	-0.0385
K02044	phnD	phosphonate transport system substrate-binding protein	G701	K02041	0.2463	K01665	-0.0432
K02045	cysA	sulfate transport system ATP-binding protein	G704	K02047	0.1770	K07727	-0.0392
K02046	cysU	sulfate transport system permease protein	G705	K02048	0.1756	K09930	-0.0308
K02047	cysW	sulfate transport system permease protein	G704	K02045	0.1770	K07727	-0.0275
K02048	cysP, sbp	sulfate transport system substrate-binding protein	G705	K02046	0.1756	K06990	-0.0318
K02049	ABC.SN.A, ssuB, tauB	sulfonate/nitrate/taurine transport system ATP-binding protein	G708	K02050	0.2759	K07282	-0.0480
K02050	ABC.SN.P, ssuC, tauC	sulfonate/nitrate/taurine transport system permease protein	G708	K02049	0.2759	K02919	-0.0534
K02051	ABC.SN.S, ssuA, tauA	sulfonate/nitrate/taurine transport system substrate-binding protein	G708	K02050	0.2006	K03827	-0.0603
K02052	ABC.SP.A	putative spermidine/putrescine transport system ATP-binding protein	G711	K02054	0.1386	K11741	-0.0573
K02053	ABC.SP.P	putative spermidine/putrescine transport system permease protein	G711	K02054	0.2916	K11070	-0.0708
K02054	ABC.SP.P1	putative spermidine/putrescine transport system permease protein	G711	K02053	0.2916	K11071	-0.0664
K02055	ABC.SP.S	putative spermidine/putrescine transport system substrate-binding	G711	K02053	0.1764	K02435	-0.0637
K02056	ABC.SS.A	simple sugar transport system ATP-binding protein	G715	K02057	0.3125	K00839	-0.0494

KEGG identifier (gene *i*)	Name	Description	Correlog group	KEGG identifier (gene *j*)	w_{ij}	KEGG identifier (gene *j'*)	$w_{ij'}$
K02057	ABC.SS.P	simple sugar transport system permease protein	G715	K02056	0.3125	K01788	-0.0422
K02058	ABC.SS.S	simple sugar transport system substrate-binding protein	G715	K02057	0.1540	K07506	-0.0632
K02063	ABC.VB1.P , thiP	thiamine transport system permease protein	G718	K02064	0.2395	K07239	-0.0332
K02064	ABC.VB1.S , tbpA	thiamine transport system substrate-binding protein	G718	K02063	0.2395	K07239	-0.0332
K02065	ABC.X1.A	putative ABC transport system ATP-binding protein	G720	K02066	0.1746	K03856	-0.0362
K02066	ABC.X1.P	putative ABC transport system permease protein	G720	K02065	0.1746	K03856	-0.0478
K02067	ABC.X1.S	putative ABC transport system substrate-binding protein	G720	K02066	0.0838	K01501	-0.0447
K02068	ABC.X2.A	putative ABC transport system ATP-binding protein	G526	K02069	0.1919	K09017	-0.0849
K02069	ABC.X2.P	putative ABC transport system permease protein	G526	K02068	0.1919	K11741	-0.0708
K02071	ABC.MET.A, metN	D-methionine transport system ATP-binding protein	G725	K02072	0.1954	K05515	-0.0525
K02072	ABC.MET.P, metI	D-methionine transport system permease protein	G725	K02073	0.2135	K05515	-0.0382
K02073	ABC.MET.S, metQ	D-methionine transport system substrate-binding protein	G725	K02072	0.2135	K02023	-0.0575
K02074	ABC.ZM.A	zinc/manganese transport system ATP-binding protein	G219	K02075	0.1912	K07127	-0.0505
K02075	ABC.ZM.P	zinc/manganese transport system permease protein	G219	K02077	0.2956	K07491	-0.0437
K02077	ABC.ZM.S	zinc/manganese transport system substrate-binding protein	G219	K02075	0.2956	K01226	-0.0493
K02106	atoE	short-chain fatty acids transporter	G84	K00248	0.0820	K05350	-0.0602
K02116	atpI	ATP synthase protein I	G649	K07403	0.0570	K01487	-0.0429
K02160	ACCB, bccP	acetyl-CoA carboxylase biotin carboxyl carrier protein	G638	K01961	0.1098	K01442	-0.0444
K02168	betT	high-affinity choline transport protein	G41	K00108	0.0925	K07118	-0.0715
K02169	bioC	biotin synthesis protein BioC	G735	K02170	0.0701	K08978	-0.0409
K02170	bioH	biotin biosynthesis protein BioH	G735	K02169	0.0701	K00101	-0.0406
K02188	cbiD	cobalamin biosynthesis protein CbiD	G737	K02189	0.1115	K01959	-0.0483
K02189	cbiG	cobalamin biosynthesis protein CbiG	G737	K05936	0.1326	K01193	-0.0395
K02192	bfd	bacterioferritin-associated ferredoxin	G150	K00389	0.0526	K00249	-0.0540
K02193	ccmA	heme exporter ATP-binding protein CcmA	G740	K02200	0.0792	K05710	-0.0343
K02194	ccmB	heme exporter membrane protein CcmB	G741	K02198	0.1172	K07399	-0.0310
K02195	ccmC	heme exporter membrane protein CcmC	G741	K02198	0.1246	K07399	-0.0306
K02196	ccmD	cytochrome c-type biogenesis protein CcmD	G30	K03556	0.0529	K07012	-0.0485

KEGG identifier (gene *i*)	Name	Description	Correlog group	KEGG identifier (gene *j*)	w_{ij}	KEGG identifier (gene *j'*)	$w_{ij'}$
K02197	ccmE	cytochrome c-type biogenesis protein CcmE	G741	K02194	0.1095	K03310	-0.0275
K02198	ccmF	cytochrome c-type biogenesis protein CcmF	G741	K02195	0.1246	K07399	-0.0299
K02199	ccmG, dsbE	cytochrome c biogenesis protein CcmG, thiol:disulfide interchange	G740	K02200	0.1064	K02040	-0.0371
K02200	ccmH	cytochrome c-type biogenesis protein CcmH	G740	K02199	0.1064	K00449	-0.0392
K02204	E2.7.1.39B, thrB	homoserine kinase type II	G437	K09924	0.0535	K00872	-0.0516
K02217	ftnA, ftn	ferritin 1	G749	K04757	0.0561	K11065	-0.0514
K02221	yggT	YggT family protein	G750	K03589	0.0580	K00966	-0.0487
K02224	cobB	hydrogenobyrinic acid a,c-diamide synthase	G146	K00837	0.0637	K07341	-0.0565
K02225	cobC1, cobC	cobalamin biosynthetic protein CobC	G752	K04719	0.0545	K01058	-0.0461
K02227	E6.3.1.10, cobD, cbiB	adenosylcobinamide-phosphate synthase CobD	G753	K02232	0.1320	K03449	-0.0381
K02229	E1.14.13.83, cobG	precorrin-3B synthase	G475	K02230	0.0599	K03795	-0.0725
K02230	cobN	cobaltochelatase CobN	G475	K03404	0.0927	K00795	-0.0432
K02231	cobP, cobU	adenosylcobinamide kinase	G248	K02233	0.1532	K03453	-0.0336
K02232	E6.3.5.10, cobQ, cbiP	adenosylcobyric acid synthase	G753	K02227	0.1320	K00795	-0.0345
K02233	E2.7.8.26, cobS, cobV	adenosylcobinamide-GDP ribazoletransferase	G248	K02231	0.1532	K00257	-0.0367
K02234	cobW	cobalamin biosynthesis protein CobW	G475	K02230	0.0701	K07497	-0.0406
K02237	comEA	competence protein ComEA	G760	K06901	0.0532	K02970	-0.0504
K02238	comEC	competence protein ComEC	G90	K01262	0.0609	K01985	-0.0615
K02242	comFC	competence protein ComFC	G28	K00058	0.0417	K01091	-0.0543
K02258	COX11	cytochrome c oxidase subunit XI assembly protein	G763	K02259	0.0693	K05916	-0.0392
K02259	COX15	cytochrome c oxidase subunit XV assembly protein	G763	K02276	0.0841	K07407	-0.0309
K02274	coxA	cytochrome c oxidase subunit I	G763	K02275	0.1442	K07289	-0.0288
K02275	coxB	cytochrome c oxidase subunit II	G763	K02274	0.1442	K01580	-0.0374
K02276	coxC	cytochrome c oxidase subunit III	G763	K02274	0.1201	K07289	-0.0323
K02278	cpaA, tadV	prepilin peptidase CpaA	G768	K02282	0.1049	K01011	-0.0483
K02279	cpaB, rcpC	pilus assembly protein CpaB	G768	K02282	0.1501	K09922	-0.0316
K02280	cpaC, rcpA	pilus assembly protein CpaC	G768	K02279	0.1260	K11741	-0.0311
K02282	cpaE, tadZ	pilus assembly protein CpaE	G768	K02279	0.1501	K01243	-0.0315
K02283	cpaF, tadA	pilus assembly protein CpaF	G772	K12511	0.1389	K06904	-0.0356
K02291	E2.5.1.32, crtB	phytoene synthase	G773	K07181	0.0460	K07645	-0.0472
K02297	cyoA	cytochrome o ubiquinol oxidase subunit II	G774	K02300	0.1689	K07458	-0.0303
K02298	cyoB	cytochrome o ubiquinol oxidase subunit I	G774	K02299	0.1591	K07313	-0.0364
K02299	cyoC	cytochrome o ubiquinol oxidase subunit III	G774	K02297	0.1615	K10680	-0.0324

KEGG identifier (gene *i*)	Name	Description	Correlog group	KEGG identifier (gene *j*)	w_{ij}	KEGG identifier (gene *j'*)	$w_{ij'}$
K02300	cyoD	cytochrome o ubiquinol oxidase operon protein cyoD	G774	K02297	0.1689	K00389	-0.0375
K02301	cyoE	protoheme IX farnesyltransferase	G763	K02274	0.0790	K01840	-0.0360
K02302	cysG	uroporphyrin-III C-methyltransferase	G320	K00957	0.0532	K02303	-0.0872
K02303	MET1, cobA	uroporphyrin-III C-methyltransferase	G542	K01749	0.0643	K02302	-0.0872
K02334	dpo	DNA polymerase bacteriophage-type	G113	K00356	0.0536	K03284	-0.0559
K02336	DPO2, polB	DNA polymerase II	G84	K02106	0.0438	K03317	-0.0408
K02339	DPO3C, holC	DNA polymerase III subunit chi	G43	K00989	0.0317	K01451	-0.0259
K02342	DPO3E, dnaQ	DNA polymerase III subunit epsilon	G90	K05566	0.0804	K07335	-0.0541
K02346	DPO4, dinB	DNA polymerase IV	G21	K06889	0.0594	K01081	-0.0515
K02347	dpx	DNA polymerase (family X)	G455	K04477	0.1951	K01847	-0.0451
K02348	elaA	ElaA protein	G787	K06888	0.0600	K05820	-0.0617
K02361	entC	isochorismate synthase	G788	K05844	0.0597	K01077	-0.0615
K02371	fabK	enoyl-[acyl carrier protein] reductase II	G789	K03777	0.0483	K04757	-0.0558
K02372	fabZ	3R-hydroxymyristoyl ACP dehydrase	G750	K02221	0.0490	K09117	-0.0502
K02377	E1.1.1.271, fcl	GDP-L-fucose synthase	G548	K01711	0.2439	K01985	-0.0535
K02379	fdhD	FdhD protein	G45	K00123	0.1149	K03969	-0.0460
K02380	fdhE	FdhE protein	G342	K03833	0.1256	K01934	-0.0563
K02386	flgA	flagella basal body P-ring formation protein FlgA	G794	K02393	0.0756	K06223	-0.0278
K02387	flgB	flagellar basal-body rod protein FlgB	G795	K02389	0.0474	K00065	-0.0211
K02388	flgC	flagellar basal-body rod protein FlgC	G795	K02389	0.0538	K00065	-0.0162
K02389	flgD	flagellar basal-body rod modification protein FlgD	G795	K02388	0.0538	K10907	-0.0229
K02390	flgE	flagellar hook protein FlgE	G795	K02388	0.0438	K00065	-0.0164
K02391	flgF	flagellar basal-body rod protein FlgF	G794	K02386	0.0494	K03563	-0.0364
K02392	flgG	flagellar basal-body rod protein FlgG	G794	K02393	0.0423	K08224	-0.0261
K02393	flgH	flagellar L-ring protein precursor FlgH	G794	K02386	0.0756	K03563	-0.0270
K02394	flgI	flagellar P-ring protein precursor FlgI	G794	K02393	0.0748	K00040	-0.0289
K02395	flgJ	flagellar protein FlgJ	G803	K03572	0.0476	K03409	-0.0565
K02396	flgK	flagellar hook-associated protein 1 FlgK	G804	K02417	0.0391	K06897	-0.0195
K02397	flgL	flagellar hook-associated protein 3 FlgL	G805	K02406	0.0601	K07313	-0.0233
K02398	flgM	negative regulator of flagellin synthesis FlgM	G806	K02413	0.0561	K07313	-0.0375

KEGG identifier (gene *i*)	Name	Description	Correlog group	KEGG identifier (gene *j*)	w_{ij}	KEGG identifier (gene *j'*)	$w_{ij'}$
K02399	flgN	flagella synthesis protein FlgN	G806	K02413	0.0449	K03563	-0.0365
K02400	flhA	flagellar biosynthesis protein FlhA	G804	K02417	0.0438	K03741	-0.0284
K02401	flhB	flagellar biosynthetic protein FlhB	G809	K02421	0.0431	K07645	-0.0146
K02404	flhF	flagellar biosynthesis protein FlhF	G810	K04562	0.0642	K03885	-0.0462
K02405	fliA	RNA polymerase sigma factor for flagellar operon FliA	G806	K02413	0.0547	K01286	-0.0302
K02406	fliC	flagellin	G805	K02397	0.0601	K07032	-0.0266
K02407	fliD	flagellar hook-associated protein 2	G813	K02422	0.0745	K07497	-0.0264
K02408	fliE	flagellar hook-basal body complex protein FliE	G814	K02420	0.0431	K03827	-0.0167
K02409	fliF	flagellar M-ring protein FliF	G805	K02397	0.0475	K00836	-0.0278
K02410	fliG	flagellar motor switch protein FliG	G816	K02556	0.0417	K01628	-0.0349
K02411	fliH	flagellar assembly protein FliH	G817	K02412	0.0459	K05820	-0.0331
K02412	fliI	flagellum-specific ATP synthase	G817	K02411	0.0459	K01269	-0.0174
K02413	fliJ	flagellar FliJ protein	G806	K02418	0.0571	K07313	-0.0377
K02414	fliK	flagellar hook-length control protein FliK	G37	K01175	0.0556	K01607	-0.0525
K02415	fliL	flagellar FliL protein	G794	K02392	0.0364	K03975	-0.0292
K02416	fliM	flagellar motor switch protein FliM	G805	K02409	0.0469	K07250	-0.0256
K02417	fliNY, fliN	flagellar motor switch protein FliN/FliY	G804	K02400	0.0438	K07460	-0.0135
K02418	fliOZ, fliO	flagellar protein FliO/FliZ	G806	K02413	0.0571	K07098	-0.0360
K02419	fliP	flagellar biosynthetic protein FliP	G816	K02556	0.0415	K02439	-0.0153
K02420	fliQ	flagellar biosynthetic protein FliQ	G814	K02408	0.0431	K01823	-0.0154
K02421	fliR	flagellar biosynthetic protein FliR	G809	K02401	0.0431	K07032	-0.0179
K02422	fliS	flagellar protein FliS	G813	K02407	0.0745	K07011	-0.0282
K02426	sufE	cysteine desulfuration protein SufE	G476	K01552	0.0407	K04488	-0.0669
K02427	ftsJ	cell division protein methyltransferase FtsJ	G830	K11749	0.0720	K01423	-0.0602
K02428	mazG	nucleoside-triphosphate pyrophosphatase	G188	K00567	0.1088	K07001	-0.0742
K02429	fucP	MFS transporter, FHS family, L-fucose permease	G179	K01188	0.0711	K11741	-0.0719
K02433	gatA	aspartyl-tRNA(Asn)/glutamyl-tRNA (Gln) amidotransferase subunit A	G833	K06179	0.1134	K01556	-0.0554
K02434	gatB	aspartyl-tRNA(Asn)/glutamyl-tRNA (Gln) amidotransferase subunit B	G833	K02433	0.1086	K01886	-0.0417
K02435	gatC	aspartyl-tRNA(Asn)/glutamyl-tRNA (Gln) amidotransferase subunit C	G833	K02434	0.0929	K02055	-0.0637
K02437	gcvH	glycine cleavage system H protein	G99	K00605	0.1038	K07067	-0.0363
K02438	glgX	glycogen operon protein GlgX	G229	K00700	0.1045	K07727	-0.0507
K02439	glpE	thiosulfate sulfurtransferase	G838	K02742	0.0333	K01501	-0.0266
K02440	GLPF	glycerol uptake facilitator protein	G42	K00864	0.1047	K07341	-0.0615
K02441	glpG	GlpG protein	G13	K07507	0.0420	K07386	-0.0322
K02444	glpR	DeoR family transcriptional regulator, glycerol-3-phosphate regulon	G22	K00661	0.0458	K07334	-0.0409

KEGG identifier (gene *i*)	Name	Description	Correlog group	KEGG identifier (gene *j*)	w_{ij}	KEGG identifier (gene *j'*)	$w_{ij'}$
K02445	glpT	MFS transporter, OPA family, glycerol-3-phosphate transporter	G5	K01753	0.0568	K00842	-0.0554
K02446	glpX	fructose-1,6-bisphosphatase II	G40	K00104	0.0453	K09933	-0.0376
K02452	gspC	general secretion pathway protein C	G844	K02460	0.0770	K02022	-0.0381
K02453	gspD	general secretion pathway protein D	G845	K02454	0.0943	K09765	-0.0342
K02454	gspE	general secretion pathway protein E	G845	K02455	0.1082	K07795	-0.0320
K02455	gspF	general secretion pathway protein F	G845	K02454	0.1082	K05946	-0.0336
K02456	gspG	general secretion pathway protein G	G845	K02454	0.0932	K03734	-0.0364
K02457	gspH	general secretion pathway protein H	G844	K02459	0.1033	K00210	-0.0350
K02458	gspI	general secretion pathway protein I	G844	K02459	0.1106	K02503	-0.0355
K02459	gspJ	general secretion pathway protein J	G844	K02458	0.1106	K07684	-0.0294
K02460	gspK	general secretion pathway protein K	G844	K02459	0.0943	K02414	-0.0406
K02461	gspL	general secretion pathway protein L	G844	K02457	0.0919	K04092	-0.0254
K02462	gspM	general secretion pathway protein M	G854	K02463	0.1136	K06904	-0.0423
K02463	gspN	general secretion pathway protein N	G854	K02462	0.1136	K01442	-0.0618
K02471	ABC.YDDA	putative ATP-binding cassette transporter	G468	K01816	0.0551	K02566	-0.0484
K02472	wecC	UDP-N-acetyl-D-mannosaminuronic acid dehydrogenase	G43	K01791	0.1095	K10536	-0.0608
K02474	wbpO	UDP-N-acetyl-D-galactosamine dehydrogenase	G858	K03328	0.0646	K07226	-0.0679
K02477	K02477	two-component system, LytT family, response regulator	G859	K02478	0.1236	K01975	-0.0588
K02478	K02478	two-component system, LytT family, sensor kinase	G859	K02477	0.1236	K01785	-0.0597
K02483	K02483	two-component system, OmpR family, response regulator	G861	K02484	0.0838	K01297	-0.0619
K02484	K02484	two-component system, OmpR family, sensor kinase	G861	K02483	0.0838	K01826	-0.0631
K02488	pleD	two-component system, PleD related family, response regulator	G863	K09816	0.0628	K07347	-0.0809
K02492	E1.2.1.70, hemA	glutamyl-tRNA reductase	G542	K01845	0.0943	K07473	-0.0336
K02494	hemM	outer membrane lipoprotein LolB	G865	K07278	0.0459	K09806	-0.0427
K02496	hemX	uroporphyrin-III C-methyltransferase	G672	K03554	0.0292	K09928	-0.0225
K02498	hemY	HemY protein	G867	K11183	0.0316	K07126	-0.0310
K02500	hisF	cyclase HisF	G6	K01814	0.1023	K05350	-0.0301
K02501	hisH	glutamine amidotransferase	G6	K02500	0.1002	K01990	-0.0254

KEGG identifier (gene *i*)	Name	Description	Correlog group	KEGG identifier (gene *j*)	w_{ij}	KEGG identifier (gene *j'*)	$w_{ij'}$
K02502	hisZ	ATP phosphoribosyltransferase regulatory subunit	G457	K01523	0.0459	K02342	-0.0408
K02503	hit	Hit-like protein involved in cell-cycle regulation	G871	K06948	0.0829	K07058	-0.0575
K02509	hpaH	2-oxo-hept-3-ene-1,7-dioate hydratase	G54	K00151	0.1719	K11811	-0.0713
K02510	hpaI	2,4-dihydroxyhept-2-ene-1,7-dioic acid aldolase	G54	K02509	0.1331	K06987	-0.0492
K02517	htrB	lipid A biosynthesis lauroyl acyltransferase	G305	K00912	0.0503	K07075	-0.0414
K02523	ispB	octaprenyl-diphosphate synthase	G875	K07004	0.0459	K00805	-0.0864
K02527	kdtA	3-deoxy-D-manno-octulosonic-acid transferase	G876	K09949	0.0479	K06904	-0.0169
K02529	lacI, galR	LacI family transcriptional regulator	G39	K01236	0.0402	K01273	-0.0420
K02533	lasT	tRNA/rRNA methyltransferase	G315	K06916	0.0502	K09165	-0.0380
K02535	lpxC	UDP-3-O-[3-hydroxymyristoyl] N-acetylglucosamine deacetylase	G224	K00748	0.0577	K01816	-0.0261
K02536	lpxD	UDP-3-O-[3-hydroxymyristoyl] glucosamine N-acyltransferase	G224	K00748	0.0593	K02342	-0.0258
K02548	menA	1,4-dihydroxy-2-naphthoate octaprenyltransferase	G521	K01911	0.0740	K01190	-0.0584
K02549	menC	O-succinylbenzoate synthase	G882	K02551	0.0853	K02483	-0.0405
K02551	menD	2-succinyl-5-enolpyruvyl-6-hydroxy-3-cyclohexene-1-carboxylate	G882	K02552	0.1036	K03186	-0.0322
K02552	menF	menaquinone-specific isochorismate synthase	G882	K02551	0.1036	K02361	-0.0364
K02553	rraA, menG	regulator of ribonuclease activity A	G508	K01637	0.0535	K07238	-0.0494
K02556	motA	chemotaxis protein MotA	G816	K02557	0.0419	K00065	-0.0181
K02557	motB	chemotaxis protein MotB	G816	K02556	0.0419	K01795	-0.0336
K02558	mpl	UDP-N-acetylmuramate: L-alanyl-gamma-D-glutamyl-meso-diaminopimelate	G471	K01537	0.0337	K02843	-0.0311
K02564	nagB, GNPDA	glucosamine-6-phosphate deaminase	G435	K01443	0.0915	K01916	-0.0502
K02566	nagD	NagD protein	G859	K02478	0.0455	K00839	-0.0686
K02567	napA	periplasmic nitrate reductase NapA	G891	K02570	0.1807	K09778	-0.0384
K02568	napB	cytochrome c-type protein NapB	G891	K02570	0.1725	K08994	-0.0344
K02569	napC	cytochrome c-type protein NapC	G891	K02570	0.1390	K08994	-0.0435
K02570	napD	periplasmic nitrate reductase NapD	G891	K02567	0.1807	K01273	-0.0348
K02575	narK, narU, nasA	MFS transporter, NNP family, nitrate/nitrite transporter	G136	K00362	0.0836	K07046	-0.0445
K02584	nifA	Nif-specific regulatory protein	G896	K02587	0.0845	K03760	-0.0513
K02585	nifB	nitrogen fixation protein NifB	G896	K02591	0.1541	K01580	-0.0336
K02586	nifD	nitrogenase molybdenum-iron protein alpha chain	G896	K02591	0.1753	K07238	-0.0310
K02587	nifE	nitrogenase molybdenum-cofactor synthesis protein NifE	G896	K02588	0.1483	K01436	-0.0378

KEGG identifier (gene *i*)	Name	Description	Correlog group	KEGG identifier (gene *j*)	w_{ij}	KEGG identifier (gene *j'*)	$w_{ij'}$
K02588	nifH	nitrogenase iron protein NifH	G896	K02591	0.1575	K08999	-0.0291
K02591	nifK	nitrogenase molybdenum-iron protein beta chain	G896	K02586	0.1753	K00966	-0.0231
K02594	nifV	homocitrate synthase NifV	G517	K01655	0.2488	K01753	-0.0311
K02609	paaA	phenylacetic acid degradation protein	G903	K02612	0.1603	K07027	-0.0254
K02610	paaB	phenylacetic acid degradation protein	G903	K02612	0.1580	K07027	-0.0249
K02611	paaC	phenylacetic acid degradation protein	G903	K02612	0.1634	K07027	-0.0251
K02612	paaD	phenylacetic acid degradation protein	G903	K02611	0.1634	K09946	-0.0250
K02613	paaE	phenylacetic acid degradation NADH oxidoreductase	G903	K02610	0.1136	K07130	-0.0421
K02614	paaI	phenylacetic acid degradation protein	G67	K00179	0.0712	K07483	-0.0516
K02616	paaX	phenylacetic acid degradation operon negative regulatory protein	G903	K02610	0.0823	K00151	-0.0448
K02619	pabC	4-amino-4-deoxychorismate lyase	G523	K01665	0.1237	K00721	-0.0537
K02621	parC	topoisomerase IV subunit A	G911	K02622	0.1176	K01159	-0.0373
K02622	parE	topoisomerase IV subunit B	G911	K02621	0.1176	K06142	-0.0760
K02624	pcaR	IclR family transcriptional regulator, pca regulon regulatory	G163	K00481	0.0749	K09930	-0.0624
K02647	cdaR	carbohydrate diacid regulator	G239	K10947	0.0620	K02004	-0.0639
K02650	pilA	type IV pilus assembly protein PilA	G459	K02663	0.0706	K03498	-0.0443
K02651	flp, pilA	pilus assembly protein Flp/PilA	G768	K02278	0.1020	K01179	-0.0540
K02652	pilB	type IV pilus assembly protein PilB	G917	K02653	0.1155	K01735	-0.0399
K02653	pilC	type IV pilus assembly protein PilC	G917	K02652	0.1155	K07814	-0.0382
K02654	pilD, pppA	leader peptidase (prepilin peptidase)	G917	K02669	0.0556	K00680	-0.0475
K02655	pilE	type IV pilus assembly protein PilE	G920	K02672	0.0608	K03296	-0.0291
K02656	pilF	type IV pilus assembly protein PilF	G920	K08084	0.0362	K06048	-0.0425
K02662	pilM	type IV pilus assembly protein PilM	G459	K02664	0.1000	K01551	-0.0333
K02663	pilN	type IV pilus assembly protein PilN	G459	K02662	0.0772	K03776	-0.0646
K02664	pilO	type IV pilus assembly protein PilO	G459	K02662	0.1000	K03816	-0.0362
K02665	pilP	type IV pilus assembly protein PilP	G920	K02671	0.0688	K09939	-0.0289
K02666	pilQ	type IV pilus assembly protein PilQ	G917	K02653	0.0654	K06001	-0.0351
K02669	pilT	twitching motility protein PilT	G917	K02652	0.0902	K06867	-0.0483
K02670	pilU	twitching motility protein PilU	G920	K02665	0.0557	K12372	-0.0316
K02671	pilV	type IV pilus assembly protein PilV	G920	K02672	0.0869	K07464	-0.0374

KEGG identifier (gene *i*)	Name	Description	Correlog group	KEGG identifier (gene *j*)	w_{ij}	KEGG identifier (gene *j'*)	$w_{ij'}$
K02672	pilW	type IV pilus assembly protein PilW	G920	K02671	0.0869	K08994	-0.0432
K02674	pilY1	type IV pilus assembly protein PilY1	G920	K02672	0.0710	K03606	-0.0487
K02686	priB	primosomal replication protein N	G161	K09939	0.0338	K00926	-0.0349
K02687	prmA	ribosomal protein L11 methyltransferase	G89	K07460	0.0499	K07473	-0.0287
K02742	sprT	SprT protein	G838	K07080	0.0340	K12267	-0.0341
K02759	PTS-Cel-EIIA, celC	PTS system, cellobiose-specific IIA component	G935	K02760	0.2169	K02445	-0.0491
K02760	PTS-Cel-EIIB, celA	PTS system, cellobiose-specific IIB component	G935	K02759	0.2169	K07491	-0.0389
K02761	PTS-Cel-EIIC, celB	PTS system, cellobiose-specific IIC component	G935	K02759	0.1589	K07084	-0.0440
K02768	PTS-Fru-EIIA, fruB	PTS system, fructose-specific IIA component	G867	K11183	0.1486	K06959	-0.0419
K02769	PTS-Fru-EIIB, fruA	PTS system, fructose-specific IIB component	G299	K02770	0.2410	K07386	-0.0369
K02770	PTS-Fru-EIIC, fruA	PTS system, fructose-specific IIC component	G299	K02769	0.2410	K07407	-0.0336
K02777	PTS-Glc-EIIA, crr	PTS system, glucose-specific IIA component	G941	K02779	0.0764	K07238	-0.0455
K02778	PTS-Glc-EIIB, ptsG	PTS system, glucose-specific IIB component	G941	K02779	0.3492	K06223	-0.0494
K02779	PTS-Glc-EIIC, ptsG	PTS system, glucose-specific IIC component	G941	K02778	0.3492	K06223	-0.0500
K02793	PTS-Man-EIIA, manX	PTS system, mannose-specific IIA component	G944	K02794	0.1560	K03300	-0.0335
K02794	PTS-Man-EIIB, manX	PTS system, mannose-specific IIB component	G944	K02795	0.1690	K00854	-0.0412
K02795	PTS-Man-EIIC, manY	PTS system, mannose-specific IIC component	G944	K02794	0.1690	K03827	-0.0345
K02796	PTS-Man-EIID, manZ	PTS system, mannose-specific IID component	G944	K02795	0.1249	K03455	-0.0355
K02798	PTS-Mtl-EIIA, mtlA	PTS system, mannitol-specific IIA component	G3	K02799	0.2174	K05995	-0.0450
K02799	PTS-Mtl-EIIB, mtlA	PTS system, mannitol-specific IIB component	G3	K02800	0.3097	K02435	-0.0453
K02800	PTS-Mtl-EIIC, mtlA	PTS system, mannitol-specific IIC component	G3	K02799	0.3097	K02435	-0.0453
K02803	PTS-Nag-EIIB, nagE	PTS system, N-acetylglucosamine-specific IIB component	G951	K02804	0.3007	K03496	-0.0356
K02804	PTS-Nag-EIIC, nagE	PTS system, N-acetylglucosamine-specific IIC component	G951	K02803	0.3007	K03823	-0.0372
K02806	PTS-Ntr-EIIA, ptsN	PTS system, nitrogen regulatory IIA component	G603	K01835	0.0389	K02002	-0.0323
K02809	PTS-Scr-EIIB, scrA	PTS system, sucrose-specific IIB component	G138	K02810	0.3904	K01007	-0.0412

KEGG identifier (gene *i*)	Name	Description	Correlog group	KEGG identifier (gene *j*)	w_{ij}	KEGG identifier (gene *j'*)	$w_{ij'}$
K02810	PTS-Scr-EIIC, scrA	PTS system, sucrose-specific IIC component	G138	K02809	0.3904	K01007	-0.0412
K02821	PTS-Ula-EIIA, ulaC, sgaA	PTS system, ascorbate-specific IIA component	G956	K02822	0.1865	K02919	-0.0514
K02822	PTS-Ula-EIIB, ulaB, sgaB	PTS system, ascorbate-specific IIB component	G956	K03475	0.2607	K00754	-0.0588
K02823	pyrDII	dihydroorotate dehydrogenase electron transfer subunit	G89	K03287	0.0835	K07075	-0.0636
K02824	pyrP, uraA	uracil permease	G381	K01524	0.0599	K07112	-0.0566
K02825	pyrR	pyrimidine operon attenuation protein	G372	K01139	0.0416	K07319	-0.0370
K02837	RF-3, prfC	peptide chain release factor RF-3	G871	K03699	0.0409	K01975	-0.0451
K02841	waaC, rfaC	heptosyltransferase I	G962	K02843	0.1048	K07075	-0.0456
K02843	waaF, rfaF	heptosyltransferase II	G962	K02841	0.1048	K07171	-0.0456
K02858	ribB	3,4-dihydroxy 2-butanone 4-phosphate synthase	G458	K01497	0.1775	K01934	-0.0431
K02907	RP-L30, rpmD	large subunit ribosomal protein L30	G965	K07568	0.0711	K00344	-0.0492
K02919	RP-L36, rpmJ	large subunit ribosomal protein L36	G161	K02970	0.0826	K12267	-0.0679
K02970	RP-S21, rpsU	small subunit ribosomal protein S21	G161	K02919	0.0826	K02237	-0.0504
K03060	rpoZ	DNA-directed RNA polymerase subunit omega	G162	K07015	0.0573	K00261	-0.0594
K03071	secB	preprotein translocase subunit SecB	G292	K03978	0.0388	K02907	-0.0375
K03072	secD	preprotein translocase subunit SecD	G970	K03074	0.1557	K12257	-0.1074
K03074	secF	preprotein translocase subunit SecF	G970	K03072	0.1557	K12257	-0.0990
K03087	SIG2, rpoS	RNA polymerase nonessential primary-like sigma factor	G24	K03741	0.0514	K05810	-0.0779
K03088	SIG3.2, rpoE	RNA polymerase sigma-70 factor, ECF subfamily	G368	K06904	0.0508	K07058	-0.0585
K03089	SIG3.3.1, rpoH	RNA polymerase sigma-32 factor	G223	K01439	0.0330	K00363	-0.0301
K03090	SIG3.3.2, sigB	RNA polymerase sigma-B factor	G749	K04757	0.0813	K02614	-0.0487
K03091	SIG3.4	RNA polymerase sporulation-specific sigma factor	G976	K07738	0.0513	K01179	-0.0637
K03092	SIG54, rpoN	RNA polymerase sigma-54 factor	G460	K01512	0.0722	K03087	-0.0489
K03098	blc	outer membrane lipoprotein Blc	G150	K09701	0.1059	K07218	-0.0613
K03113	SUI1	translation initiation factor SUI1	G858	K02474	0.0368	K00389	-0.0380
K03116	tatA	sec-independent protein translocase protein TatA	G980	K03118	0.1809	K06201	-0.0500
K03117	tatB	sec-independent protein translocase protein TatB	G90	K07090	0.0386	K01580	-0.0344

KEGG identifier (gene *i*)	Name	Description	Correlog group	KEGG identifier (gene *j*)	w_{ij}	KEGG identifier (gene *j'*)	$w_{ij'}$
K03118	tatC	sec-independent protein translocase protein TatC	G980	K03116	0.1809	K06864	-0.0334
K03119	E1.14.11.17, tauD	taurine dioxygenase	G468	K03307	0.0540	K07120	-0.0748
K03147	thiC	thiamine biosynthesis protein ThiC	G297	K00941	0.0673	K00005	-0.0456
K03148	thiF	adenylyltransferase ThiF	G985	K03149	0.0802	K09686	-0.0504
K03149	thiG	thiamine biosynthesis ThiG	G985	K03154	0.1899	K00878	-0.0453
K03150	thiH	thiamine biosynthesis ThiH	G985	K03148	0.0705	K03153	-0.0550
K03151	thiI	thiamine biosynthesis protein ThiI	G988	K09762	0.0679	K03700	-0.0577
K03152	thiJ	4-methyl-5(b-hydroxyethyl)-thiazole monophosphate biosynthesis	G113	K00356	0.0597	K03700	-0.0999
K03153	thiO	glycine oxidase	G985	K03154	0.0802	K03150	-0.0550
K03154	thiS	thiamine biosynthesis ThiS	G985	K03149	0.1899	K00878	-0.0475
K03169	topB	DNA topoisomerase III	G486	K10536	0.0585	K07497	-0.0633
K03179	ubiA	4-hydroxybenzoate octaprenyltransferase	G993	K03186	0.0650	K01661	-0.0401
K03181	ubiC	chorismate--pyruvate lyase	G92	K09806	0.0449	K07313	-0.0443
K03182	ubiD	3-octaprenyl-4-hydroxybenzoate carboxy-lyase UbiD	G993	K03186	0.2216	K03497	-0.0442
K03183	ubiE	ubiquinone/menaquinone biosynthesis methyltransferase	G521	K02548	0.0505	K07584	-0.0490
K03184	ubiF	2-octaprenyl-3-methyl-6-methoxy-1,4-benzoquinol hydroxylase	G489	K07034	0.0393	K06134	-0.0546
K03185	ubiH	2-octaprenyl-6-methoxyphenol hydroxylase	G993	K03182	0.0419	K07334	-0.0272
K03186	ubiX	3-octaprenyl-4-hydroxybenzoate carboxy-lyase UbiX	G993	K03182	0.2216	K03497	-0.0501
K03187	ureE	urease accessory protein	G1000	K03189	0.1164	K07341	-0.0394
K03188	ureF	urease accessory protein	G1000	K03189	0.1449	K01438	-0.0223
K03189	ureG	urease accessory protein	G1000	K03190	0.1456	K05595	-0.0207
K03190	ureD, ureH	urease accessory protein	G1000	K03189	0.1456	K05595	-0.0207
K03192	ureJ	urease accessory protein	G442	K01474	0.0571	K00151	-0.0544
K03195	virB10	type IV secretion system protein VirB10	G1005	K03204	0.1533	K01934	-0.0361
K03196	virB11	type IV secretion system protein VirB11	G1006	K03197	0.1440	K01934	-0.0382
K03197	virB2	type IV secretion system protein VirB2	G1006	K03196	0.1440	K02669	-0.0462
K03198	virB3	type IV secretion system protein VirB3	G189	K00568	0.1419	K00951	-0.0537
K03199	virB4	type IV secretion system protein VirB4	G1005	K03195	0.1318	K00754	-0.0532
K03200	virB5	type IV secretion system protein VirB5	G1006	K03197	0.1330	K01686	-0.0547
K03203	virB8	type IV secretion system protein VirB8	G1005	K03199	0.1265	K03446	-0.0524
K03204	virB9	type IV secretion system protein VirB9	G1005	K03195	0.1533	K01934	-0.0353
K03205	virD4	type IV secretion system protein VirD4	G1006	K03196	0.1060	K01447	-0.0563

KEGG identifier (gene *i*)	Name	Description	Correlog group	KEGG identifier (gene *j*)	w_{ij}	KEGG identifier (gene *j'*)	$w_{ij'}$
K03215	rumA	RNA methyltransferase, TrmA family	G190	K06148	0.0388	K06867	-0.0324
K03216	cspR	RNA methyltransferase, TrmH family, group 2	G13	K00760	0.0502	K03325	-0.0542
K03269	lpxH	UDP-2,3-diacylglucosamine hydrolase	G75	K01772	0.0289	K09949	-0.0540
K03270	kdsC	3-deoxy-D-manno-octulosonate 8-phosphate phosphatase (KDO 8-P	G1017	K07567	0.0369	K01295	-0.0314
K03271	gmhA	phosphoheptose isomerase	G962	K03274	0.0735	K01153	-0.0422
K03272	gmhC, hldE, waaE, rfaE	D-beta-D-heptose 7-phosphate kinase	G962	K03274	0.0684	K01623	-0.0321
K03273	gmhB	D-glycero-D-manno-heptose 1,7-bisphosphate phosphatase	G962	K03271	0.0531	K09017	-0.0485
K03274	rfaD	ADP-L-glycero-D-manno-heptose 6-epimerase	G962	K02843	0.0735	K06910	-0.0473
K03281	TC.CIC	chloride channel protein, CIC family	G13	K07497	0.0619	K09922	-0.0610
K03282	TC.MSCL	large conductance mechanosensitive channel, MscL family	G1017	K05995	0.0714	K06442	-0.0563
K03284	TC.MIT	metal ion transporter, MIT family	G52	K06223	0.0731	K00925	-0.0678
K03286	TC.OOP	OmpA-OmpF porin, OOP family	G149	K07323	0.0546	K06876	-0.0466
K03287	TC.OMF	outer membrane factor, OMF family	G89	K01768	0.0845	K00156	-0.0716
K03293	TC.AAT	amino acid transporter, AAT family	G55	K09936	0.0495	K06153	-0.0412
K03294	TC.APA, frlA	basic amino acid/polyamine antiporter, APA family	G532	K05916	0.0518	K02647	-0.0576
K03295	TC.CDF	cation efflux system protein, CDF family	G13	K07313	0.0654	K00852	-0.0568
K03296	TC.HAE1	hydrophobic/amphiphilic exporter-1 (mainly G- bacteria), HAE1 family	G1030	K12340	0.0882	K00836	-0.0502
K03297	TC.SMR	small multidrug resistance protein, SMR family	G221	K00666	0.0759	K07130	-0.0750
K03299	TC.GNTP	gluconate:H+ symporter, GntP family	G187	K05993	0.0606	K01426	-0.0545
K03300	TC.CITMHS	citrate-Mg2+:H+ or citrate-Ca2+:H+ symporter, CitMHS family	G398	K05966	0.0832	K00520	-0.0594
K03303	TC.LCTP	lactate transporter, LctP family	G251	K00782	0.1143	K07464	-0.0596
K03305	TC.POT	proton-dependent oligopeptide transporter, POT family	G5	K02445	0.0557	K06985	-0.0598
K03306	TC.PIT	inorganic phosphate transporter, PiT family	G1036	K07220	0.1765	K00389	-0.0762
K03307	TC.SSS	solute:Na+ symporter, SSS family	G468	K01788	0.0674	K06901	-0.0558
K03308	TC.NSS	neurotransmitter:Na+ symporter, NSS family	G28	K03310	0.0612	K07737	-0.0478
K03310	TC.AGCS	alanine or glycine:cation symporter, AGCS family	G28	K07238	0.0614	K06925	-0.0638

KEGG identifier (gene *i*)	Name	Description	Correlog group	KEGG identifier (gene *j*)	w_{ij}	KEGG identifier (gene *j'*)	$w_{ij'}$
K03311	TC.LIVCS	branched-chain amino acid:cation transporter, LIVCS family	G54	K01744	0.0601	K00661	-0.0552
K03312	TC.ESS	glutamate:Na+ symporter, ESS family	G24	K03741	0.0673	K00248	-0.0587
K03313	TC.NHAA, nhaA	Na+:H+ antiporter, NhaA family	G184	K00558	0.0674	K07275	-0.0612
K03315	TC.NHAC, nhaC	Na+:H+ antiporter, NhaC family	G1017	K05995	0.0519	K01426	-0.0457
K03316	TC.CPA1	monovalent cation:H+ antiporter, CPA1 family	G368	K01187	0.0887	K07301	-0.0502
K03317	TC.CNT	concentrative nucleoside transporter, CNT family	G226	K00839	0.0621	K00657	-0.0653
K03320	TC.AMT	ammonium transporter, Amt family	G715	K02058	0.0568	K01520	-0.0688
K03321	TC.SULP	sulfate permease, SulP family	G187	K01795	0.0694	K01533	-0.0662
K03322	mntH	manganese transport protein	G395	K03790	0.0642	K00428	-0.0573
K03325	TC.ACR3	arsenite transporter, ACR3 family	G366	K06897	0.0644	K00721	-0.0612
K03327	TC.MATE	multidrug resistance protein, MATE family	G1050	K03745	0.0517	K00528	-0.0611
K03328	TC.PST	polysaccharide transporter, PST family	G858	K02474	0.0646	K07737	-0.0716
K03335	iolE	inosose dehydratase	G4	K03336	0.1846	K00540	-0.0373
K03336	iolD	3D-(3,5/4)-trihydroxycyclohexane-1,2-dione hydrolase	G4	K03335	0.1846	K03885	-0.0317
K03337	iolB	5-deoxy-glucuronate isomerase	G1054	K03338	0.2004	K06925	-0.0364
K03338	iolC	5-dehydro-2-deoxygluconokinase	G1054	K03337	0.2004	K07473	-0.0427
K03382	E3.5.99.3	hydroxyatrazine ethylaminohydrolase	G1056	K07127	0.0794	K06001	-0.0593
K03385	nrfA	formate-dependent nitrite reductase, periplasmic cytochrome c552	G161	K00428	0.0526	K07182	-0.0542
K03386	E1.11.1.15, PRDX, ahpC	peroxiredoxin (alkyl hydroperoxide reductase subunit C)	G189	K03198	0.0847	K01010	-0.0623
K03387	ahpF	alkyl hydroperoxide reductase subunit F	G475	K09771	0.0537	K07458	-0.0510
K03402	argR, ahrC	transcriptional regulator of arginine metabolism	G93	K01556	0.0703	K05515	-0.0423
K03404	chlD	magnesium chelatase subunit ChlD	G475	K03405	0.1757	K07506	-0.0522
K03405	chlI	magnesium chelatase subunit ChlI	G475	K03404	0.1757	K08156	-0.0420
K03406	mcp	methyl-accepting chemotaxis protein	G1063	K03407	0.0820	K02484	-0.0482
K03407	cheA	two-component system, chemotaxis family, sensor kinase CheA	G1063	K03408	0.0980	K04047	-0.0267
K03408	cheW	purine-binding chemotaxis protein CheW	G1063	K03407	0.0980	K07047	-0.0338
K03409	cheX	chemotaxis protein CheX	G24	K01269	0.0772	K00795	-0.0571
K03410	cheC	chemotaxis protein CheC	G41	K02168	0.0482	K07075	-0.0464
K03411	cheD	chemotaxis protein CheD	G67	K04767	0.0578	K07347	-0.0426

KEGG identifier (gene *i*)	Name	Description	Correlog group	KEGG identifier (gene *j*)	w_{ij}	KEGG identifier (gene *j'*)	$w_{ij'}$
K03412	cheB	protein-glutamate methylesterase, two-component system, chemotaxis	G193	K00575	0.1669	K02106	-0.0394
K03413	cheY	two-component system, chemotaxis family, response regulator CheY	G1063	K03407	0.0612	K03926	-0.0295
K03414	cheZ	chemotaxis protein CheZ	G773	K03415	0.0404	K09005	-0.0341
K03415	cheV	two-component system, chemotaxis family, response regulator CheV	G773	K07181	0.0768	K12942	-0.0607
K03417	E4.1.3.30, prpB	methylisocitrate lyase	G520	K01720	0.1456	K00019	-0.0514
K03426	E3.6.1.22, NUDT12, nudC	NAD+ diphosphatase	G358	K07166	0.0504	K00567	-0.0439
K03427	hsdM	type I restriction enzyme M protein	G15	K01153	0.3793	K07458	-0.0492
K03431	glmM	phosphoglucosamine mutase	G1076	K04042	0.0738	K03116	-0.0467
K03435	fruR1, fruR	LacI family transcriptional regulator, fructose operon	G867	K11183	0.0736	K00632	-0.0519
K03436	fruR2, fruR	DeoR family transcriptional regulator, fructose operon	G299	K02770	0.0796	K11183	-0.0465
K03437	spoU	RNA methyltransferase, TrmH family	G684	K02023	0.0632	K01552	-0.0578
K03439	E2.1.1.33	tRNA (guanine-N7-)-methyltransferase	G146	K06867	0.0644	K02003	-0.0559
K03442	TC.MSCS	small conductance mechanosensitive ion channel, MscS family	G1081	K07150	0.0611	K03707	-0.0593
K03446	emrB	MFS transporter, DHA2 family, multidrug resistance protein B	G92	K03543	0.1337	K03203	-0.0524
K03449	MFS.CP	MFS transporter, CP family, cyanate transporter	G357	K01077	0.0577	K01423	-0.0631
K03453	TC.BASS	bile acid:Na+ symporter, BASS family	G471	K08978	0.0840	K00520	-0.0721
K03455	TC.KEF	monovalent cation:H+ antiporter-2, CPA2 family	G182	K06287	0.0600	K00262	-0.0606
K03457	TC.NCS1	nucleobase:cation symporter-1, NCS1 family	G34	K01487	0.0742	K06904	-0.0675
K03458	TC.NCS2	nucleobase:cation symporter-2, NCS2 family	G226	K00839	0.0760	K08974	-0.0588
K03465	E2.1.1.148, thyX, thy1	thymidylate synthase (FAD)	G760	K06901	0.1010	K00560	-0.1122
K03469	E3.1.26.4A, RNASEH1, rnhA	ribonuclease HI	G1089	K08972	0.0587	K00874	-0.0496
K03473	E1.1.1.290, pdxB	erythronate-4-phosphate dehydrogenase	G226	K03683	0.0322	K03777	-0.0287
K03474	E2.6.99.2, pdxJ	pyridoxine 5-phosphate synthase	G35	K00097	0.0587	K08681	-0.0334

KEGG identifier (gene *i*)	Name	Description	Correlog group	KEGG identifier (gene *j*)	w_{ij}	KEGG identifier (gene *j'*)	$w_{ij'}$
K03475	PTS-Ula-EIIC, ulaA, sgaT	PTS system, ascorbate-specific IIC component	G956	K02822	0.2607	K00754	-0.0627
K03478	ydjC	hypothetical protein	G388	K01222	0.1055	K09967	-0.0581
K03496	parA, soj	chromosome partitioning protein	G1094	K03497	0.1648	K01226	-0.0825
K03497	parB, spo0J	chromosome partitioning protein, ParB family	G1094	K03496	0.1648	K02010	-0.0702
K03498	trkH	trk system potassium uptake protein TrkH	G413	K03499	0.1704	K00432	-0.0678
K03499	trkA	trk system potassium uptake protein TrkA	G413	K03498	0.1704	K05985	-0.0535
K03500	rsmB, sun	ribosomal RNA small subunit methyltransferase B	G956	K03475	0.0528	K00230	-0.0356
K03502	DPO5C, umuC	DNA polymerase V	G1099	K03503	0.2899	K01529	-0.0685
K03503	DPO5D, umuD	DNA polymerase V	G1099	K03502	0.2899	K06199	-0.0631
K03517	nadA	quinolinate synthase	G98	K00767	0.1904	K03282	-0.0469
K03518	E1.2.99.2S, coxS	carbon-monoxide dehydrogenase small subunit	G1102	K03519	0.2296	K01744	-0.0526
K03519	E1.2.99.2M, cutM, coxM	carbon-monoxide dehydrogenase medium subunit	G1102	K03518	0.2296	K09958	-0.0413
K03521	fixA, etfB	electron transfer flavoprotein beta subunit	G108	K03522	0.2136	K05993	-0.0341
K03522	fixB, etfA	electron transfer flavoprotein alpha subunit	G108	K03521	0.2136	K02433	-0.0324
K03523	bioY	putative biotin biosynthesis protein BioY	G36	K00919	0.0864	K01114	-0.0550
K03525	coaX	type III pantothenate kinase	G90	K02238	0.0594	K00867	-0.1015
K03526	E1.17.7.1, gcpE, ispG	(E)-4-hydroxy-3-methylbut-2-enyl-diphosphate synthase	G36	K03527	0.1610	K02907	-0.0432
K03527	E1.17.1.2, lytB, ispH	4-hydroxy-3-methylbut-2-enyl diphosphate reductase	G36	K03526	0.1610	K01823	-0.0547
K03528	zipA	cell division protein ZipA	G1110	K03563	0.0325	K00523	-0.0248
K03529	smc	chromosome segregation protein	G1111	K05896	0.1535	K00560	-0.0321
K03534	rhaM	L-rhamnose mutarotase	G396	K05350	0.0740	K00285	-0.0739
K03543	emrA	multidrug resistance protein A	G92	K03446	0.1337	K09939	-0.0546
K03546	sbcC	exonuclease SbcC	G1114	K03547	0.2833	K01179	-0.0455
K03547	sbcD	exonuclease SbcD	G1114	K03546	0.2833	K09930	-0.0450
K03548	perM	putative permease	G174	K05810	0.0645	K01297	-0.0680
K03549	kup	KUP system potassium uptake protein	G226	K05782	0.0458	K03498	-0.0456
K03554	rdgC	recombination associated protein RdgC	G672	K11312	0.0378	K03672	-0.0450
K03555	mutS	DNA mismatch repair protein MutS	G803	K03572	0.1172	K02008	-0.0445
K03556	malT	LuxR family transcriptional regulator, maltose regulon positive	G30	K00065	0.0612	K01436	-0.0594
K03557	fis	Fis family transcriptional regulator, factor for inversion	G976	K07738	0.0315	K00571	-0.0267

KEGG identifier (gene *i*)	Name	Description	Correlog group	KEGG identifier (gene *j*)	w_{ij}	KEGG identifier (gene *j'*)	$w_{ij'}$
K03558	cvpA	membrane protein required for colicin V production	G1111	K05896	0.0723	K03087	-0.0651
K03559	exbD	biopolymer transport protein ExbD	G1123	K03561	0.0513	K06194	-0.0386
K03560	tolR	biopolymer transport protein TolR	G26	K07320	0.0240	K01659	-0.0189
K03561	exbB	biopolymer transport protein ExbB	G1123	K03559	0.0513	K07684	-0.0450
K03562	tolQ	biopolymer transport protein TolQ	G1126	K03641	0.0729	K09008	-0.0452
K03563	csrA	carbon storage regulator	G1110	K04061	0.0400	K01580	-0.0379
K03564	BCP, PRXQ, DOT5	peroxiredoxin Q/BCP	G52	K03284	0.0716	K01322	-0.0466
K03565	recX	regulatory protein	G1129	K03704	0.0434	K05516	-0.0570
K03566	gcvA	LysR family transcriptional regulator, glycine cleavage system	G11	K06076	0.0521	K01081	-0.0365
K03567	gcvR	glycine cleavage system transcriptional repressor	G476	K07028	0.0433	K03919	-0.0372
K03568	tldD	TldD protein	G1132	K03592	0.1792	K09157	-0.0399
K03569	mreB	rod shape-determining protein MreB and related proteins	G1133	K05837	0.1199	K01586	-0.0579
K03570	mreC	rod shape-determining protein MreC	G1133	K03569	0.1144	K09008	-0.0566
K03571	mreD	rod shape-determining protein MreD	G1133	K03570	0.0701	K01159	-0.0870
K03572	mutL	DNA mismatch repair protein MutL	G803	K03555	0.1172	K06925	-0.0606
K03574	MUTT, NUDT1, MTH1	7,8-dihydro-8-oxoguanine triphosphatase	G414	K01447	0.0923	K07778	-0.0739
K03575	mutY	A/G-specific adenine glycosylase	G13	K07313	0.0483	K07464	-0.0447
K03576	metR	LysR family transcriptional regulator, regulator for metE and metH	G181	K00549	0.0571	K03782	-0.0352
K03577	acrR	TetR/AcrR family transcriptional regulator, acrAB operon repressor	G1140	K08310	0.0457	K01795	-0.0470
K03578	hrpA	ATP-dependent helicase HrpA	G1141	K03684	0.0344	K04750	-0.0325
K03579	hrpB	ATP-dependent helicase HrpB	G219	K00660	0.0412	K09017	-0.0405
K03580	hepA	ATP-dependent helicase HepA	G358	K10805	0.0326	K03197	-0.0365
K03581	recD	exodeoxyribonuclease V alpha subunit	G207	K03582	0.1054	K01533	-0.0591
K03582	recB	exodeoxyribonuclease V beta subunit	G207	K03583	0.1752	K07443	-0.0303
K03583	recC	exodeoxyribonuclease V gamma subunit	G207	K03582	0.1752	K00549	-0.0322
K03585	acrA	membrane fusion protein	G334	K00983	0.0409	K02919	-0.0377
K03586	ftsL	cell division protein FtsL	G490	K07645	0.0315	K05794	-0.0250
K03587	ftsI	cell division protein FtsI (penicillin-binding protein 3)	G500	K01620	0.0588	K06985	-0.0743
K03589	ftsQ	cell division protein FtsQ	G750	K02221	0.0580	K00818	-0.0587
K03590	ftsA	cell division protein FtsA	G750	K03589	0.0443	K03695	-0.0355
K03592	pmbA	PmbA protein	G1132	K03568	0.1792	K01930	-0.0641
K03593	mrp	ATP-binding protein involved in chromosome partitioning	G52	K03564	0.0580	K00891	-0.0383
K03594	bfr	bacterioferritin	G150	K02192	0.0523	K03602	-0.0533

KEGG identifier (gene *i*)	Name	Description	Correlog group	KEGG identifier (gene *j*)	w_{ij}	KEGG identifier (gene *j'*)	$w_{ij'}$
K03597	rseA	sigma-E factor negative regulatory protein RseA	G1155	K03598	0.0415	K03098	-0.0289
K03598	rseB	sigma-E factor negative regulatory protein RseB	G1155	K03597	0.0415	K09922	-0.0362
K03599	sspA	stringent starvation protein A	G1157	K03672	0.0355	K07127	-0.0343
K03600	sspB	stringent starvation protein B	G1157	K03672	0.0255	K03590	-0.0204
K03602	xseB	exodeoxyribonuclease VII small subunit	G241	K08301	0.1013	K02040	-0.0743
K03605	hyaD	hydrogenase 1 maturation protease	G303	K03620	0.1793	K03741	-0.0548
K03606	wcaJ	putative colanic acid biosysnthesis UDP-glucose lipid carrier	G396	K01674	0.0648	K01512	-0.0617
K03607	proQ	ProP effector	G344	K03893	0.0541	K03435	-0.0506
K03608	minE	cell division topological specificity factor	G1163	K03609	0.1277	K06001	-0.0426
K03609	minD	septum site-determining protein MinD	G1163	K03610	0.1477	K07012	-0.0298
K03610	minC	septum site-determining protein MinC	G1163	K03609	0.1477	K06213	-0.0351
K03611	dsbB	disulfide bond formation protein DsbB	G1166	K05540	0.0540	K00970	-0.0401
K03612	rnfG	electron transport complex protein RnfG	G379	K03617	0.1199	K09931	-0.0279
K03613	rnfE	electron transport complex protein RnfE	G379	K03617	0.1166	K08998	-0.0597
K03614	rnfD	electron transport complex protein RnfD	G379	K03617	0.1211	K09931	-0.0234
K03615	rnfC	electron transport complex protein RnfC	G379	K03614	0.1151	K06910	-0.0302
K03616	rnfB	electron transport complex protein RnfB	G1171	K09801	0.0594	K03707	-0.0286
K03617	rnfA	electron transport complex protein RnfA	G379	K03614	0.1211	K09931	-0.0271
K03620	hyaC	Ni/Fe-hydrogenase 1 B-type cytochrome subunit	G303	K03605	0.1793	K07243	-0.0547
K03621	plsX	fatty acid/phospholipid synthesis protein	G1174	K08591	0.1436	K00100	-0.0379
K03629	recF	DNA replication and repair protein RecF	G36	K00919	0.0794	K03926	-0.0469
K03630	radC	DNA repair protein RadC	G1176	K07347	0.0859	K01154	-0.0539
K03634	lolA	outer membrane lipoproteins carrier protein	G219	K01414	0.0345	K03621	-0.0318
K03635	moaE	molybdenum cofactor biosynthesis protein E	G1178	K03636	0.0914	K07407	-0.0319
K03636	moaD	molybdenum cofactor biosynthesis protein D	G1178	K03635	0.0914	K01010	-0.0337
K03637	moaC	molybdenum cofactor biosynthesis protein C	G1180	K03639	0.1378	K07029	-0.0375
K03638	moaB	molybdenum cofactor biosynthesis protein B	G281	K00965	0.0516	K03831	-0.0690
K03639	moaA	molybdenum cofactor biosynthesis protein A	G1180	K03637	0.1378	K02548	-0.0286

KEGG identifier (gene *i*)	Name	Description	Correlog group	KEGG identifier (gene *j*)	w_{ij}	KEGG identifier (gene *j'*)	$w_{ij'}$
K03640	pal	peptidoglycan-associated lipoprotein	G90	K01262	0.0423	K02919	-0.0470
K03641	tolB	TolB protein	G1126	K03562	0.0729	K02003	-0.0582
K03642	rlpA	rare lipoprotein A	G993	K03186	0.0439	K01356	-0.0394
K03643	lptE, rlpB	LPS-assembly lipoprotein	G1186	K11719	0.0284	K00970	-0.0167
K03644	lipA	lipoic acid synthetase	G1187	K03801	0.1253	K07067	-0.0500
K03646	tolA	colicin import membrane protein	G26	K06966	0.0410	K03577	-0.0418
K03647	nrdI	protein involved in ribonucleotide reduction	G174	K00526	0.1610	K01674	-0.0464
K03648	UNG, UDG	uracil-DNA glycosylase	G173	K01709	0.0440	K02334	-0.0537
K03651	icc	Icc protein	G980	K07443	0.0408	K09136	-0.0498
K03652	MPG	DNA-3-methyladenine glycosylase	G1192	K03926	0.0758	K05515	-0.0476
K03654	recQ	ATP-dependent DNA helicase RecQ	G22	K07727	0.0578	K00005	-0.0434
K03656	rep	ATP-dependent DNA helicase Rep	G24	K03409	0.0350	K00556	-0.0299
K03665	hflX	GTP-binding protein HflX	G1195	K06217	0.0884	K01686	-0.0506
K03666	hfq	host factor-I protein	G292	K03978	0.0612	K07568	-0.0326
K03667	hslU	ATP-dependent HslUV protease ATP-binding subunit HslU	G419	K01419	0.1731	K00757	-0.0399
K03669	mdoH	membrane glycosyltransferase	G1198	K03670	0.2612	K07181	-0.0464
K03670	mdoG	periplasmic glucans biosynthesis protein	G1198	K03669	0.2612	K07181	-0.0474
K03672	trxC	thioredoxin 2	G1157	K07095	0.0598	K01720	-0.0591
K03673	dsbA	thiol:disulfide interchange protein DsbA	G292	K06153	0.0493	K07568	-0.0401
K03676	grxC, GLRX, GLRX2	glutaredoxin 3	G161	K02970	0.0459	K09939	-0.0545
K03677	cysQ	CysQ protein	G289	K00860	0.0705	K02168	-0.0429
K03683	rnt	ribonuclease T	G226	K08316	0.0329	K00262	-0.0219
K03684	rnd	ribonuclease D	G1141	K12297	0.0349	K00556	-0.0398
K03688	ubiB, aarF	ubiquinone biosynthesis protein	G189	K00568	0.0421	K08602	-0.0456
K03690	yigP	hypothetical protein	G475	K03387	0.0350	K07322	-0.0386
K03694	clpA	ATP-dependent Clp protease ATP-binding subunit ClpA	G227	K06891	0.0691	K07101	-0.0466
K03695	clpB	ATP-dependent Clp protease ATP-binding subunit ClpB	G238	K00756	0.0539	K03885	-0.0699
K03696	clpC	ATP-dependent Clp protease ATP-binding subunit ClpC	G803	K03572	0.0595	K01795	-0.0495
K03698	cbf	CMP-binding protein	G615	K01886	0.0527	K12257	-0.0517
K03699	tlyC	putative hemolysin	G871	K06948	0.0737	K07003	-0.0544
K03700	recU	recombination protein U	G1213	K07024	0.0771	K03152	-0.0999
K03704	cspA	cold shock protein (beta-ribbon, CspA family)	G1129	K09760	0.0715	K03700	-0.0771
K03705	hrcA	heat-inducible transcriptional repressor	G368	K03088	0.0484	K01129	-0.0398
K03707	tenA	transcriptional activator TenA	G254	K00878	0.0981	K00823	-0.0595
K03708	ctsR	transcriptional regulator CtsR	G36	K06962	0.0501	K03527	-0.0388

KEGG identifier (gene *i*)	Name	Description	Correlog group	KEGG identifier (gene *j*)	w_{ij}	KEGG identifier (gene *j'*)	$w_{ij'}$
K03710	K03710	GntR family transcriptional regulator	G162	K00950	0.0665	K03778	-0.0487
K03711	fur	Fur family transcriptional regulator, ferric uptake regulator	G396	K05350	0.0664	K12257	-0.0511
K03712	marR	MarR family transcriptional regulator	G471	K01537	0.0665	K00005	-0.0592
K03717	nhaR	LysR family transcriptional regulator, transcriptional activator of	G683	K07274	0.0434	K03415	-0.0476
K03718	asnC	Lrp/AsnC family transcriptional regulator, regulator for asnA, asnC	G200	K01114	0.0536	K02168	-0.0480
K03719	lrp	Lrp/AsnC family transcriptional regulator, leucine-responsive	G13	K03809	0.0514	K02445	-0.0506
K03721	tyrR	transcriptional regulator of aroF, aroG, tyrA and aromatic amino	G11	K08984	0.0324	K06896	-0.0282
K03722	dinG	ATP-dependent DNA helicase DinG	G1133	K03571	0.0570	K12506	-0.0424
K03724	lhr	ATP-dependent helicase Lhr and Lhr-like helicase	G90	K02342	0.0509	K07795	-0.0552
K03732	rhlB	ATP-dependent RNA helicase RhlB	G532	K07334	0.0332	K03975	-0.0284
K03733	xerC	integrase/recombinase XerC	G86	K01966	0.0570	K02484	-0.0454
K03734	apbE	thiamine biosynthesis lipoprotein	G161	K09939	0.0596	K12942	-0.0568
K03735	E4.3.1.7L, eutB	ethanolamine ammonia-lyase large subunit	G1230	K03736	0.3157	K07213	-0.0373
K03736	E4.3.1.7S, eutC	ethanolamine ammonia-lyase small subunit	G1230	K03735	0.3157	K06889	-0.0402
K03737	E1.2.7.-P, nifJ	putative pyruvate-flavodoxin oxidoreductase	G985	K03150	0.0513	K07002	-0.0540
K03741	ARSC2, arsC	arsenate reductase	G24	K06001	0.0734	K03605	-0.0548
K03742	cinA	competence/damage-inducible protein CinA	G21	K00046	0.0461	K03743	-0.0972
K03743	K03743		G188	K00567	0.0677	K03742	-0.0972
K03744	lemA	LemA protein	G1236	K06872	0.1367	K01423	-0.0530
K03745	slyX	SlyX protein	G1050	K03327	0.0517	K06137	-0.0350
K03746	hns	DNA-binding protein H-NS	G292	K01776	0.0491	K09806	-0.0508
K03747	smg	Smg protein	G138	K00364	0.0461	K00154	-0.0425
K03749	dedD	DedD protein	G442	K07288	0.0484	K08224	-0.0314
K03750	moeA	molybdopterin biosynthesis protein MoeA	G1180	K03639	0.1252	K00651	-0.0314
K03751	moeB	molybdopterin biosynthesis protein MoeB	G1242	K04774	0.0519	K02224	-0.0454
K03752	mobA	molybdopterin-guanine dinucleotide biosynthesis protein A	G1178	K03635	0.0683	K03496	-0.0475
K03753	mobB	molybdopterin-guanine dinucleotide biosynthesis protein B	G1178	K03752	0.0595	K09922	-0.0467
K03758	arcD	arginine:ornithine antiporter	G200	K01478	0.0951	K12267	-0.0709
K03760	eptA	phosphoethanolamine transferase	G373	K05349	0.0499	K02584	-0.0513
K03761	kgtP	MFS transporter, MHS family, alpha-ketoglutarate permease	G200	K01114	0.0678	K01779	-0.0658

KEGG identifier (gene *i*)	Name	Description	Correlog group	KEGG identifier (gene *j*)	w_{ij}	KEGG identifier (gene *j'*)	$w_{ij'}$
K03762	proP	MFS transporter, MHS family, proline/betaine transporter	G101	K00344	0.0723	K00260	-0.0629
K03763	DPO3A2, polC	DNA polymerase III subunit alpha, Gram-positive type	G162	K07584	0.0464	K07164	-0.0370
K03767	PPIA	peptidyl-prolyl cis-trans isomerase A (cyclophilin A)	G425	K01426	0.0585	K03458	-0.0515
K03768	PPIB, ppiB	peptidyl-prolyl cis-trans isomerase B (cyclophilin B)	G96	K06978	0.0594	K02058	-0.0498
K03769	ppiC	peptidyl-prolyl cis-trans isomerase C	G204	K00925	0.0547	K10563	-0.0618
K03770	ppiD	peptidyl-prolyl cis-trans isomerase D	G13	K07258	0.0485	K09125	-0.0405
K03771	surA	peptidyl-prolyl cis-trans isomerase SurA	G750	K03589	0.0381	K00124	-0.0276
K03772	fkpA	FKBP-type peptidyl-prolyl cis-trans isomerase FkpA	G37	K01175	0.0541	K09134	-0.0524
K03773	fklB	FKBP-type peptidyl-prolyl cis-trans isomerase FklB	G161	K00428	0.0428	K09765	-0.0367
K03774	slpA	FKBP-type peptidyl-prolyl cis-trans isomerase SlpA	G672	K02010	0.0387	K03665	-0.0324
K03775	slyD	FKBP-type peptidyl-prolyl cis-trans isomerase SlyD	G251	K00782	0.0383	K06182	-0.0386
K03776	aer	aerotaxis receptor	G304	K00906	0.0533	K02663	-0.0646
K03777	dld	D-lactate dehydrogenase	G789	K07046	0.0729	K07118	-0.0773
K03778	ldhA	D-lactate dehydrogenase	G0	K00001	0.0650	K01417	-0.0549
K03781	katE, CAT	catalase	G10	K01560	0.0614	K03782	-0.0820
K03782	katG	catalase/peroxidase	G354	K07798	0.0810	K03781	-0.0820
K03783	punA	purine-nucleoside phosphorylase	G210	K00640	0.0455	K03784	-0.0543
K03784	deoD	purine-nucleoside phosphorylase	G604	K01839	0.0795	K03783	-0.0543
K03785	aroD	3-dehydroquinate dehydratase I	G7	K00014	0.0753	K03786	-0.2188
K03786	aroQ, qutE	3-dehydroquinate dehydratase II	G7	K00014	0.0581	K03785	-0.2188
K03787	surE	5'-nucleotidase	G113	K06147	0.0439	K09967	-0.0576
K03789	rimI	ribosomal-protein-alanine N-acetyltransferase	G162	K01091	0.0513	K05516	-0.0413
K03790	rimJ	ribosomal-protein-alanine N-acetyltransferase	G395	K10778	0.0754	K07171	-0.0524
K03795	cbiX	sirohydrochlorin cobaltochelatase	G194	K00593	0.0824	K02229	-0.0725
K03797	E3.4.21.102, prc, ctpA	carboxyl-terminal processing protease	G249	K06726	0.0526	K07021	-0.0453
K03799	htpX	heat shock protein HtpX	G1236	K03744	0.0546	K01104	-0.0547
K03800	lplA	lipoate-protein ligase A	G8	K00627	0.0781	K07038	-0.0522
K03801	lipB	lipoyl(octanoyl) transferase	G1187	K03644	0.1253	K03800	-0.0486
K03803	rseC	sigma-E factor negative regulatory protein RseC	G379	K03615	0.0611	K01739	-0.0411
K03806	ampD	AmpD protein	G173	K00523	0.0380	K09765	-0.0354
K03808	pqiA	paraquat-inducible protein A	G1278	K09857	0.1325	K11177	-0.0498
K03809	wrbA	Trp repressor binding protein	G13	K09927	0.0553	K04063	-0.0479
K03811	pnuC	nicotinamide mononucleotide transporter	G190	K00952	0.0786	K07491	-0.0555
K03814	mtgA	monofunctional biosynthetic peptidoglycan transglycosylase	G789	K03777	0.0468	K07814	-0.0389

KEGG identifier (gene *i*)	Name	Description	Correlog group	KEGG identifier (gene *j*)	w_{ij}	KEGG identifier (gene *j'*)	$w_{ij'}$
K03816	xpt	xanthine phosphoribosyltransferase	G226	K03458	0.0662	K08974	-0.0386
K03820	lnt	apolipoprotein N-acyltransferase	G487	K01586	0.0554	K04042	-0.0419
K03821	phbC, phaC	polyhydroxyalkanoate synthase	G364	K01724	0.0720	K00567	-0.0865
K03823	pat	phosphinothricin acetyltransferase	G179	K00547	0.0708	K05847	-0.0662
K03824	yhbS	putative acetyltransferase	G875	K07004	0.0730	K05521	-0.0633
K03827	yjaB	putative acetyltransferase	G55	K01442	0.0841	K12308	-0.0795
K03831	mog	molybdopterin biosynthesis protein Mog	G297	K00941	0.0451	K03638	-0.0690
K03832	tonB	periplasmic protein TonB	G676	K02014	0.0481	K03672	-0.0328
K03833	selB	selenocysteine-specific elongation factor	G342	K01042	0.2505	K01479	-0.0472
K03837	sdaC	serine transporter	G411	K06956	0.0635	K03777	-0.0490
K03839	fldA	flavodoxin I	G875	K07004	0.0568	K00694	-0.0520
K03841	FBP, fbp	fructose-1,6-bisphosphatase I	G282	K01624	0.0446	K01676	-0.0359
K03856	AROA2, aroA	3-deoxy-7-phosphoheptulonate synthase	G162	K01633	0.0938	K01626	-0.0783
K03885	ndh	NADH dehydrogenase	G52	K03593	0.0549	K03695	-0.0699
K03892	arsR	ArsR family transcriptional regulator	G266	K00818	0.0685	K00680	-0.0783
K03893	arsB	arsenical pump membrane protein	G344	K07156	0.0817	K09922	-0.0767
K03919	alkB	alkylated DNA repair protein	G269	K10680	0.0459	K03534	-0.0550
K03924	moxR	MoxR-like ATPase	G230	K07114	0.0567	K01934	-0.0482
K03925	mraZ	MraZ protein	G373	K05349	0.0526	K08974	-0.0614
K03926	cutA	periplasmic divalent cation tolerance protein	G1192	K03652	0.0758	K07010	-0.0548
K03927	PNBA	carboxylesterase type B	G196	K06016	0.0621	K06999	-0.0629
K03928	yvaK	carboxylesterase	G344	K03893	0.0635	K07009	-0.0541
K03929	K03929	putative esterase	G38	K00121	0.0903	K07011	-0.0579
K03969	pspA	phage shock protein A	G229	K00700	0.0521	K09936	-0.0501
K03973	pspC	phage shock protein C	G281	K00965	0.0792	K09819	-0.0729
K03975	dedA	membrane-associated protein	G50	K00135	0.0627	K04565	-0.0470
K03976	ebsC	putative transcription regulator	G28	K03308	0.0477	K03312	-0.0436
K03978	engB	GTP-binding protein	G292	K06941	0.0687	K03497	-0.0483
K03980	mviN	virulence factor	G1133	K03569	0.0538	K01586	-0.0504
K03981	dsbC	thiol:disulfide interchange protein DsbC	G373	K03760	0.0362	K03672	-0.0303
K04042	glmU	bifunctional protein GlmU	G1076	K03431	0.0738	K06925	-0.0558
K04044	hscA	molecular chaperone HscA	G1313	K04082	0.0858	K09013	-0.0336
K04046	yegD	hypothetical chaperone protein	G752	K02225	0.0412	K06001	-0.0528
K04047	dps	starvation-inducible DNA-binding protein	G12	K00023	0.0600	K01447	-0.0637
K04061	flhB2	flagellar biosynthesis protein	G1110	K06603	0.0791	K01092	-0.0348
K04063	osmC	osmotically inducible protein OsmC	G490	K07645	0.0575	K07506	-0.0620
K04068	nrdG	anaerobic ribonucleoside-triphosphate reductase activating protein	G175	K00527	0.0804	K00837	-0.0600
K04069	pflA, pflC, pflE	pyruvate formate lyase activating enzyme	G216	K00656	0.1421	K10680	-0.0493

KEGG identifier (gene *i*)	Name	Description	Correlog group	KEGG identifier (gene *j*)	w_{ij}	KEGG identifier (gene *j'*)	$w_{ij'}$
K04072	E1.2.1.10A, adhE	acetaldehyde dehydrogenase	G0	K00001	0.0870	K07458	-0.0480
K04074	divIVA	cell division initiation protein	G388	K03478	0.0446	K11175	-0.0497
K04079	htpG, HSP90A	molecular chaperone HtpG	G73	K01919	0.0502	K08223	-0.0505
K04080	ibpA	molecular chaperone IbpA	G292	K06997	0.0309	K00459	-0.0269
K04082	HSCB, HSC20	molecular chaperone HscB	G1313	K04044	0.0858	K09013	-0.0386
K04083	hslO	molecular chaperone Hsp33	G393	K06442	0.0551	K07473	-0.0440
K04084	dsbD	thiol:disulfide interchange protein DsbD	G489	K07034	0.0424	K07684	-0.0451
K04085	tusA, sirA	tRNA 2-thiouridine synthesizing protein A	G55	K00154	0.0484	K07684	-0.0468
K04087	hflC	membrane protease subunit HflC	G1328	K04088	0.1183	K03785	-0.0284
K04088	hflK	membrane protease subunit HflK	G1328	K04087	0.1183	K03785	-0.0296
K04090	E1.2.7.8	indolepyruvate ferredoxin oxidoreductase	G87	K00253	0.0654	K01247	-0.0544
K04091	E1.14.14.5, ssuD	alkanesulfonate monooxygenase	G106	K00299	0.0766	K06996	-0.0603
K04092	TYRA1, tyrA	chorismate mutase	G70	K04517	0.0796	K00864	-0.0422
K04093	PHEA1, pheA	chorismate mutase	G1333	K04518	0.1215	K07737	-0.0381
K04094	gid	glucose inhibited division protein Gid	G252	K00783	0.0549	K01262	-0.0573
K04097	gst	glutathione S-transferase	G351	K07140	0.0616	K11741	-0.0504
K04477	K04477	PHP family protein	G455	K02347	0.1951	K07395	-0.0446
K04486	E3.1.3.15B	histidinol-phosphatase (PHP family)	G265	K00817	0.0606	K03300	-0.0484
K04488	nifU	nitrogen fixation protein NifU and related proteins	G1313	K04044	0.0594	K02426	-0.0669
K04517	TYRA2, tyrA	prephenate dehydrogenase	G70	K04092	0.0796	K00210	-0.0951
K04518	PHEA2, pheA	prephenate dehydratase	G1333	K04093	0.1215	K03761	-0.0445
K04561	norB	nitric-oxide reductase, cytochrome b-containing subunit I	G838	K07218	0.0819	K05595	-0.0688
K04562	flhG, fleN	flagellar biosynthesis protein FlhG	G810	K02404	0.0642	K02106	-0.0328
K04564	E1.15.1.1A, sodA, sodB, SOD2	superoxide dismutase, Fe-Mn family	G1343	K07566	0.0629	K08998	-0.0537
K04565	E1.15.1.1C, sodC, SOD1	Cu/Zn superoxide dismutase	G556	K09967	0.0736	K11105	-0.0618
K04566	lysK	lysyl-tRNA synthetase, class I	G589	K01802	0.0620	K04567	-0.1243
K04567	KARS, lysS	lysyl-tRNA synthetase, class II	G173	K03648	0.0400	K04566	-0.1243
K04568	poxA	lysyl-tRNA synthetase, class II	G606	K01843	0.0816	K07506	-0.0346
K04651	hypA	hydrogenase nickel incorporation protein HypA	G1348	K04652	0.1586	K01269	-0.0289

KEGG identifier (gene *i*)	Name	Description	Correlog group	KEGG identifier (gene *j*)	w_{ij}	KEGG identifier (gene *j'*)	$w_{ij'}$
K04652	hypB	hydrogenase nickel incorporation protein HypB	G1348	K04651	0.1586	K00025	-0.0282
K04653	hypC	hydrogenase expression/formation protein HypC	G1350	K04654	0.1426	K01589	-0.0205
K04654	hypD	hydrogenase expression/formation protein HypD	G1350	K04653	0.1426	K02050	-0.0196
K04655	hypE	hydrogenase expression/formation protein HypE	G1352	K04656	0.1560	K06994	-0.0265
K04656	hypF	hydrogenase maturation protein HypF	G1352	K04655	0.1560	K03973	-0.0386
K04691	hhoB, degS	serine protease DegS	G54	K01826	0.0441	K01259	-0.0324
K04719	E1.16.8.1	cob(II)yrinic acid a,c-diamide reductase	G752	K02225	0.0545	K09975	-0.0504
K04744	lptD, imp, ostA	LPS-assembly protein	G162	K03060	0.0370	K07171	-0.0244
K04750	phnB	PhnB protein	G444	K06979	0.0724	K05966	-0.0577
K04751	glnB	nitrogen regulatory protein P-II 1	G266	K03892	0.0669	K08974	-0.0486
K04752	glnK	nitrogen regulatory protein P-II 2	G89	K03287	0.0418	K00547	-0.0445
K04754	vacJ	lipoprotein	G149	K07323	0.0574	K08978	-0.0315
K04755	fdx	ferredoxin, 2Fe-2S	G1166	K05540	0.0822	K06903	-0.0593
K04757	rsbW	anti-sigma B factor	G749	K03090	0.0813	K02371	-0.0558
K04758	feoA	ferrous iron transport protein A	G1363	K04759	0.2508	K07223	-0.0503
K04759	feoB	ferrous iron transport protein B	G1363	K04758	0.2508	K07243	-0.0529
K04760	greB	transcription elongation factor GreB	G94	K06958	0.0337	K07126	-0.0351
K04761	oxyR	LysR family transcriptional regulator, hydrogen peroxide-inducible	G37	K00257	0.0436	K05592	-0.0431
K04762	hslR	ribosome-associated heat shock protein Hsp15	G54	K09858	0.0460	K00842	-0.0418
K04764	ihfA, himA	integration host factor subunit alpha	G52	K00930	0.0581	K09125	-0.0509
K04767	acuB	acetoin utilization protein AcuB	G67	K02614	0.0646	K07182	-0.0776
K04768	acuC	acetoin utilization protein AcuC	G471	K03712	0.0466	K12410	-0.0529
K04773	sppA	protease IV	G315	K01029	0.0598	K07115	-0.0606
K04774	sohB	serine protease SohB	G1242	K03751	0.0519	K03926	-0.0327
K05337	fer	ferredoxin	G161	K02919	0.0634	K00050	-0.0613
K05343	E5.4.99.16	maltose alpha-D-glucosyltransferase	G39	K06044	0.1101	K07341	-0.0626
K05349	bglX	beta-glucosidase	G373	K01804	0.0583	K08987	-0.0680
K05350	bglB	beta-glucosidase	G396	K03534	0.0740	K01750	-0.0671
K05365	mrcB	penicillin-binding protein 1B	G26	K03646	0.0368	K09749	-0.0265
K05366	mrcA	penicillin-binding protein 1A	G249	K03797	0.0500	K01930	-0.0464
K05367	pbpC	penicillin-binding protein 1C	G1379	K06894	0.3083	K07080	-0.0474
K05375	mbtH	MbtH protein	G139	K00366	0.0813	K08156	-0.0547
K05396	dcyD	D-cysteine desulfhydrase	G351	K01053	0.0706	K06876	-0.0611
K05499	cytR	LacI family transcriptional regulator, repressor for deo operon,	G223	K01439	0.0558	K01611	-0.0549
K05501	slmA, ttk	TetR/AcrR family transcriptional regulator	G54	K02510	0.0385	K01795	-0.0401
K05515	mrdA	penicillin-binding protein 2	G1133	K05837	0.1050	K07037	-0.0678

KEGG identifier (gene *i*)	Name	Description	Correlog group	KEGG identifier (gene *j*)	w_{ij}	KEGG identifier (gene *j'*)	$w_{ij'}$
K05516	cbpA	curved DNA-binding protein	G1157	K03672	0.0591	K03565	-0.0570
K05520	pfpI	protease I	G475	K01551	0.0721	K01761	-0.0628
K05521	draG	ADP-ribosylglycohydrolase	G1017	K05995	0.0889	K03824	-0.0633
K05522	nei	endonuclease VIII	G396	K01674	0.0548	K05801	-0.0433
K05524	fdxA	ferredoxin	G459	K01501	0.0390	K01058	-0.0337
K05527	bolA	BolA protein	G1390	K06190	0.0312	K03435	-0.0285
K05539	dusA	tRNA-dihydrouridine synthase A	G53	K09008	0.0388	K01259	-0.0356
K05540	dusB	tRNA-dihydrouridine synthase B	G1166	K04755	0.0822	K10563	-0.0658
K05541	dusC	tRNA-dihydrouridine synthase C	G162	K07814	0.0502	K03606	-0.0387
K05559	phaA	multicomponent K+:H+ antiporter subunit A	G1394	K05563	0.1111	K03119	-0.0398
K05560	phaC	multicomponent K+:H+ antiporter subunit C	G1394	K05561	0.1490	K11811	-0.0286
K05561	phaD	multicomponent K+:H+ antiporter subunit D	G1394	K05560	0.1490	K03284	-0.0310
K05562	phaE	multicomponent K+:H+ antiporter subunit E	G1394	K05560	0.1324	K06048	-0.0314
K05563	phaF	multicomponent K+:H+ antiporter subunit F	G1394	K05562	0.1233	K03442	-0.0329
K05564	phaG	multicomponent K+:H+ antiporter subunit G	G1394	K05562	0.1251	K03919	-0.0302
K05566	mnhB	multicomponent Na+:H+ antiporter subunit B	G90	K05569	0.1501	K07021	-0.0520
K05567	mnhC	multicomponent Na+:H+ antiporter subunit C	G1401	K05568	0.1619	K00540	-0.0332
K05568	mnhD	multicomponent Na+:H+ antiporter subunit D	G1401	K05567	0.1619	K06137	-0.0407
K05569	mnhE	multicomponent Na+:H+ antiporter subunit E	G90	K05570	0.1599	K02669	-0.0432
K05570	mnhF	multicomponent Na+:H+ antiporter subunit F	G90	K05571	0.1809	K00818	-0.0563
K05571	mnhG	multicomponent Na+:H+ antiporter subunit G	G90	K05570	0.1809	K09803	-0.0435
K05589	ftsB	cell division protein FtsB	G226	K03549	0.0386	K01624	-0.0337
K05591	dbpA	ATP-independent RNA helicase DbpA	G72	K00219	0.0500	K07334	-0.0627
K05592	deaD	ATP-dependent RNA helicase DeaD	G397	K09698	0.0658	K03203	-0.0501
K05595	marC	multiple antibiotic resistance protein	G226	K03458	0.0571	K04561	-0.0688
K05596	iciA	LysR family transcriptional regulator, chromosome initiation	G1410	K06895	0.1422	K01825	-0.0483
K05685	ABC.MAC.P	macrolide transport system permease protein	G1411	K05686	0.3765	K07006	-0.0458
K05686	ABC.MAC.A	macrolide transport system ATP-binding protein	G1411	K05685	0.3765	K07006	-0.0385
K05710	hcaC	ferredoxin subunit of phenylpropionate dioxygenase	G30	K12308	0.0783	K07722	-0.0645
K05772	ABC.TG.S	putative tungstate transport system substrate-binding protein	G1414	K05773	0.2862	K11209	-0.0485
K05773	ABC.TG.P	putative tungstate transport system permease protein	G1414	K05772	0.2862	K02477	-0.0381

KEGG identifier (gene *i*)	Name	Description	Correlog group	KEGG identifier (gene *j*)	w_{ij}	KEGG identifier (gene *j'*)	$w_{ij'}$
K05782	benE	benzoate membrane transport protein	G226	K03458	0.0637	K00042	-0.0882
K05786	rarD	chloramphenicol-sensitive protein RarD	G1081	K07150	0.0647	K09958	-0.0486
K05788	ihfB, himD	integration host factor subunit beta	G1418	K06194	0.0509	K05837	-0.0551
K05794	terC	tellurite resistance protein TerC	G414	K01447	0.0878	K09861	-0.0575
K05799	pdhR	GntR family transcriptional regulator, transcriptional repressor for	G489	K01628	0.0567	K05349	-0.0600
K05800	ybaO	Lrp/AsnC family transcriptional regulator	G406	K01284	0.0660	K03589	-0.0408
K05801	djlA	DnaJ like chaperone protein	G54	K00824	0.0416	K07456	-0.0787
K05805	creA	CreA protein	G1423	K06918	0.0351	K01768	-0.0414
K05807	comL	putative lipoprotein	G52	K04764	0.0257	K03602	-0.0249
K05808	yhbH	putative sigma-54 modulation protein	G1425	K09825	0.0690	K03778	-0.0438
K05810	yfiH	conserved hypothetical protein	G174	K03647	0.0807	K03087	-0.0779
K05812	yfiP	conserved hypothetical protein	G240	K00758	0.0672	K00558	-0.0489
K05813	ugpB	sn-glycerol 3-phosphate transport system substrate-binding protein	G1428	K05815	0.1936	K03973	-0.0479
K05814	ugpA	sn-glycerol 3-phosphate transport system permease protein	G1428	K05815	0.2479	K03091	-0.0391
K05815	ugpE	sn-glycerol 3-phosphate transport system permease protein	G1428	K05814	0.2479	K03091	-0.0389
K05816	ugpC	sn-glycerol 3-phosphate transport system ATP-binding protein	G1428	K05815	0.1253	K06888	-0.0399
K05820	hcaT	MFS transporter, PPP family, 3-phenylpropionic acid transporter	G2	K00005	0.0555	K02348	-0.0617
K05832	ABC.X4.P	putative ABC transport system permease protein	G653	K05833	0.3062	K01262	-0.0338
K05833	ABC.X4.A	putative ABC transport system ATP-binding protein	G653	K05832	0.3062	K10563	-0.0616
K05836	hutC	GntR family transcriptional regulator, histidine utilization	G441	K01712	0.0625	K01805	-0.0409
K05837	rodA, mrdB	rod shape determining protein RodA	G1133	K03569	0.1199	K05788	-0.0551
K05838	ybbN	putative thioredoxin	G172	K00520	0.0539	K07567	-0.0406
K05844	rimK	ribosomal protein S6 modification protein	G788	K02361	0.0597	K03328	-0.0598
K05845	ABC.BCP1.S	osmoprotectant transport system substrate-binding protein	G1439	K05846	0.2776	K07483	-0.0509
K05846	ABC.BCP1.P	osmoprotectant transport system permease protein	G1439	K05845	0.2776	K00042	-0.0416
K05847	ABC.BCP1.A	osmoprotectant transport system ATP-binding protein	G1439	K05846	0.1421	K03823	-0.0662
K05878	dhaK	dihydroxyacetone kinase, N-terminal domain	G1442	K05879	0.4037	K02055	-0.0425
K05879	dhaL	dihydroxyacetone kinase, C-terminal domain	G1442	K05878	0.4037	K07473	-0.0438
K05895	E1.3.1.54, cobK, cbiJ	precorrin-6X reductase	G737	K06042	0.0750	K01055	-0.0425

KEGG identifier (gene *i*)	Name	Description	Correlog group	KEGG identifier (gene *j*)	w_{ij}	KEGG identifier (gene *j'*)	$w_{ij'}$
K05896	scpA	segregation and condensation protein A	G1111	K03529	0.1535	K03087	-0.0582
K05916	E1.14.12.17, YHB1, hmp	nitric oxide dioxygenase	G532	K09803	0.0540	K07115	-0.0629
K05934	E2.1.1.131, cobJ, cbiH	precorrin-3B C17-methyltransferase	G737	K02189	0.1061	K00428	-0.0388
K05936	E2.1.1.133, cobM, cbiF	precorrin-4 C11-methyltransferase	G737	K06042	0.1358	K01975	-0.0265
K05939	E2.3.1.40, aas	acyl-[acyl-carrier-protein]-phospholipid O-acyltransferase	G624	K01909	0.2418	K07799	-0.0500
K05946	E2.4.1.187, tagA	N-acetylglucosaminyldiphosphoundecaprenol	G43	K01791	0.0718	K01119	-0.0575
K05966	E2.7.8.25, citG	triphosphoribosyl-dephospho-CoA synthase	G398	K01644	0.1713	K00598	-0.0635
K05982	E3.1.21.7, nfi	deoxyribonuclease V	G351	K07140	0.0605	K00523	-0.0494
K05985	E3.1.26.8, rnmV	ribonuclease M5	G1453	K06967	0.0803	K03499	-0.0535
K05993	E3.3.2.1	isochorismatase	G187	K01795	0.0813	K11747	-0.0656
K05995	pepE	dipeptidase E	G1017	K05521	0.0889	K00030	-0.0606
K06001	E4.2.1.20B B, trpB	tryptophan synthase beta chain	G24	K03409	0.0741	K03973	-0.0715
K06013	E3.4.24.84	STE24 endopeptidase	G529	K01676	0.0532	K07238	-0.0768
K06016	E3.5.1.87	N-carbamoyl-L-amino-acid hydrolase	G196	K01464	0.0688	K03216	-0.0507
K06023	HPRK, ptsK	HPr kinase/phosphorylase	G1453	K05985	0.0560	K07473	-0.0392
K06024	scpB	segregation and condensation protein B	G1111	K03529	0.1507	K04564	-0.0436
K06041	E5.3.1.13	arabinose-5-phosphate isomerase	G332	K01627	0.0858	K02380	-0.0319
K06042	E5.4.1.2, cobH, cbiC	precorrin-8X methylmutase	G737	K05936	0.1358	K07737	-0.0226
K06044	E5.4.99.15, treY, glgY	(1->4)-alpha-D-glucan 1-alpha-D-glucosylmutase	G39	K01236	0.1930	K00971	-0.0685
K06045	E5.4.99.17, sqhC, shc	squalene-hopene cyclase	G179	K07282	0.0507	K09125	-0.0479
K06048	ybdK	carboxylate-amine ligase	G139	K00366	0.0449	K01188	-0.0430
K06075	slyA	MarR family transcriptional regulator, transcriptional regulator for	G490	K07645	0.0649	K01685	-0.0504
K06076	fadL	long-chain fatty acid transport protein	G11	K00632	0.0593	K01674	-0.0654
K06077	slyB	outer membrane lipoprotein SlyB	G161	K09939	0.0544	K05591	-0.0481
K06131	cls	cardiolipin synthase	G1129	K03704	0.0564	K09705	-0.0523
K06134	COQ7	ubiquinone biosynthesis monooxygenase Coq7	G459	K07018	0.0354	K03184	-0.0546
K06137	pqqC	pyrroloquinoline-quinone synthase	G286	K00855	0.0620	K02010	-0.0703
K06140	rnk	regulator of nucleoside diphosphate kinase	G1472	K11741	0.0791	K01053	-0.0581

KEGG identifier (gene *i*)	Name	Description	Correlog group	KEGG identifier (gene *j*)	w_{ij}	KEGG identifier (gene *j'*)	$w_{ij'}$
K06142	hlpA, ompH	outer membrane protein	G36	K03629	0.0672	K01159	-0.0797
K06145	gntR	LacI family transcriptional regulator, gluconate utilization system	G150	K06876	0.0551	K07093	-0.0446
K06147	ABCB-BAC	ATP-binding cassette, subfamily B, bacterial	G113	K07021	0.0751	K00756	-0.0743
K06148	ABCC-BAC	ATP-binding cassette, subfamily C, bacterial	G190	K01529	0.0738	K02032	-0.0732
K06149	uspA	universal stress protein A	G13	K07483	0.0434	K07213	-0.0470
K06153	E3.6.1.27, bacA	undecaprenyl-diphosphatase	G292	K01776	0.0533	K01778	-0.0525
K06158	ABCF3	ATP-binding cassette, sub-family F, member 3	G28	K03310	0.0451	K00259	-0.0480
K06162	phnM	PhnM protein	G1480	K06167	0.1044	K00917	-0.0533
K06167	phnP	PhnP protein	G1480	K06162	0.1044	K03060	-0.0510
K06168	miaB	bifunctional enzyme involved in thiolation and methylation of tRNA	G89	K01784	0.0458	K01188	-0.0550
K06169	miaE	tRNA-(ms[2]io[6]A)-hydroxylase	G396	K03606	0.0449	K07004	-0.0482
K06175	truC	tRNA pseudouridine synthase C	G354	K01058	0.0443	K01991	-0.0482
K06176	truD	tRNA pseudouridine synthase D	G1094	K03497	0.0384	K05337	-0.0428
K06177	rluA	ribosomal large subunit pseudouridine synthase A	G1486	K06181	0.0586	K07171	-0.0497
K06179	rluC	ribosomal large subunit pseudouridine synthase C	G833	K02433	0.1134	K03602	-0.0582
K06181	rluE	ribosomal large subunit pseudouridine synthase E	G1486	K06177	0.0586	K05847	-0.0429
K06182	rluF	ribosomal large subunit pseudouridine synthase F	G107	K00302	0.0449	K07005	-0.0519
K06183	rsuA	ribosomal small subunit pseudouridine synthase A	G459	K02663	0.0691	K07506	-0.0678
K06186	smpA	small protein A	G529	K06013	0.0274	K03785	-0.0217
K06188	aqpZ	aquaporin Z	G366	K06897	0.0607	K09701	-0.0582
K06189	corC	magnesium and cobalt transporter	G26	K03646	0.0291	K07334	-0.0244
K06190	ispZ	intracellular septation protein	G1390	K05527	0.0312	K01524	-0.0291
K06191	nrdH	glutaredoxin-like protein NrdH	G174	K03647	0.1304	K03630	-0.0370
K06192	pqiB	paraquat-inducible protein B	G1278	K09857	0.2135	K07727	-0.0496
K06193	phnA	phosphonoacetate hydrolase	G1497	K09768	0.0625	K07263	-0.0474
K06194	nlpD	lipoprotein NlpD	G1418	K05788	0.0509	K07052	-0.0450
K06195	apaG	ApaG protein	G1499	K07657	0.0336	K03197	-0.0372
K06196	ccdA	cytochrome c-type biogenesis protein	G281	K00965	0.0447	K10680	-0.0422
K06199	crcB	CrcB protein	G24	K03741	0.0706	K03503	-0.0631
K06200	cstA	carbon starvation protein	G84	K02106	0.0659	K01273	-0.0659
K06201	cutC	copper homeostasis protein	G468	K01788	0.0715	K10041	-0.0707
K06202	cyaY	CyaY protein	G830	K11749	0.0830	K01423	-0.0645
K06203	cysZ	CysZ protein	G351	K05982	0.0454	K07018	-0.0395
K06204	dksA	DnaK suppressor protein	G525	K01666	0.0302	K00952	-0.0325
K06206	sfsA	sugar fermentation stimulation protein A	G865	K07278	0.0456	K12267	-0.0546
K06213	mgtE	magnesium transporter	G470	K07240	0.0577	K01520	-0.0621

KEGG identifier (gene *i*)	Name	Description	Correlog group	KEGG identifier (gene *j*)	w_{ij}	KEGG identifier (gene *j'*)	$w_{ij'}$
K06215	pdxS, pdx1	pyridoxine biosynthesis protein	G1509	K08681	0.1634	K00097	-0.0352
K06217	phoH, phoL	phosphate starvation-inducible protein PhoH and related proteins	G1195	K03665	0.0884	K03856	-0.0355
K06223	dam	DNA adenine methylase	G52	K00930	0.0810	K01297	-0.0810
K06281	E1.12.99.6L	hydrogenase large subunit	G1512	K06282	0.2640	K00661	-0.0379
K06282	E1.12.99.6S	hydrogenase small subunit	G1512	K06281	0.2640	K00626	-0.0390
K06287	maf	septum formation protein	G182	K03455	0.0600	K03799	-0.0513
K06346	jag	spoIIIJ-associated protein	G1515	K06960	0.0867	K02342	-0.0390
K06381	spoIID	stage II sporulation protein D	G13	K01356	0.0348	K03734	-0.0390
K06412	spoVG	stage V sporulation protein G	G162	K07015	0.0490	K12573	-0.0447
K06442	tlyA	putative hemolysin	G393	K01243	0.0609	K03282	-0.0563
K06445	fadE	acyl-CoA dehydrogenase	G1005	K03203	0.0546	K01295	-0.0436
K06447	astD	succinylglutamic semialdehyde dehydrogenase	G222	K00673	0.1149	K02471	-0.0400
K06518	cidA	holin-like protein	G1521	K09790	0.0646	K11209	-0.0606
K06603	flaG	flagellar protein FlaG	G1110	K04061	0.0791	K08224	-0.0404
K06726	rbsD	D-ribose pyranase	G249	K00852	0.0855	K03695	-0.0578
K06861	lptB	lipopolysaccharide export system ATP-binding protein	G224	K02536	0.0386	K01520	-0.0299
K06864	K06864		G1525	K06898	0.2054	K03118	-0.0334
K06867	K06867		G146	K01046	0.0740	K01706	-0.0798
K06871	K06871		G146	K06867	0.0591	K01451	-0.0665
K06872	K06872		G1236	K03744	0.1367	K01840	-0.0563
K06873	K06873		G204	K00625	0.1515	K03700	-0.0935
K06876	K06876		G150	K09701	0.0901	K07118	-0.0679
K06877	K06877		G13	K09793	0.0543	K03800	-0.0416
K06878	K06878		G138	K06890	0.0632	K02028	-0.0535
K06879	queF	7-cyano-7-deazaguanine reductase	G559	K06920	0.1232	K07124	-0.0449
K06881	K06881		G282	K00850	0.0354	K01679	-0.0347
K06885	K06885		G162	K07584	0.0667	K00756	-0.0704
K06886	glbN	hemoglobin	G183	K01854	0.0543	K06016	-0.0492
K06888	K06888		G787	K02348	0.0600	K04752	-0.0439
K06889	K06889		G21	K01295	0.0629	K07154	-0.0595
K06890	K06890		G138	K01575	0.0636	K01531	-0.0530
K06891	clpS	ATP-dependent Clp protease adaptor protein ClpS	G227	K03694	0.0691	K01676	-0.0308
K06894	K06894		G1379	K05367	0.3083	K03704	-0.0608
K06895	K06895		G1410	K05596	0.1422	K01273	-0.0577
K06896	K06896		G396	K03534	0.0623	K00851	-0.0567
K06897	K06897		G366	K03325	0.0644	K03827	-0.0533
K06898	K06898		G1525	K09121	0.2243	K01304	-0.0323
K06899	K06899		G11	K08984	0.0412	K06013	-0.0317
K06901	pbuG	putative MFS transporter, AGZA family, xanthine/uracil permease	G760	K03465	0.1010	K03307	-0.0558
K06902	UMF1, yxiO	MFS transporter, UMF1 family	G22	K07727	0.0574	K01010	-0.0480
K06903	K06903		G59	K06907	0.3842	K04755	-0.0593
K06904	K06904		G368	K01187	0.0706	K01436	-0.0693
K06907	K06907		G59	K06903	0.3842	K01744	-0.0522

KEGG identifier (gene *i*)	Name	Description	Correlog group	KEGG identifier (gene *j*)	w_{ij}	KEGG identifier (gene *j'*)	$w_{ij'}$
K06910	K06910		G409	K07115	0.0857	K10543	-0.0536
K06911	K06911		G279	K07305	0.0433	K00958	-0.0550
K06915	K06915		G1554	K09946	0.0627	K00949	-0.0499
K06916	K06916		G315	K01028	0.0586	K01659	-0.0518
K06917	selU	tRNA 2-selenouridine synthase	G342	K01008	0.1339	K03885	-0.0458
K06918	K06918		G1423	K08990	0.0821	K01295	-0.0357
K06920	queC	queuosine biosynthesis protein QueC	G559	K06879	0.1232	K00557	-0.0340
K06923	K06923		G46	K00127	0.0434	K09760	-0.0416
K06925	K06925		G468	K01788	0.0901	K03310	-0.0638
K06929	K06929		G13	K07483	0.0779	K01442	-0.0628
K06938	K06938		G226	K05782	0.0481	K07243	-0.0386
K06940	K06940		G13	K07341	0.0517	K00019	-0.0591
K06941	rlmN	ribosomal RNA large subunit methyltransferase N	G292	K01776	0.0759	K03811	-0.0420
K06948	K06948		G871	K02503	0.0829	K09698	-0.0688
K06949	rsgA, engC	ribosome biogenesis GTPase	G200	K00611	0.0470	K01768	-0.0522
K06950	K06950		G190	K00571	0.0542	K00599	-0.0694
K06956	K06956		G411	K03837	0.0635	K03885	-0.0673
K06958	K06958		G94	K00528	0.0454	K01425	-0.0435
K06959	K06959		G616	K01914	0.0507	K03496	-0.0475
K06960	K06960		G1515	K06346	0.0867	K03652	-0.0382
K06962	K06962		G36	K00616	0.0611	K00842	-0.0421
K06966	K06966		G26	K01652	0.0547	K00758	-0.0542
K06967	K06967		G1453	K05985	0.0803	K01299	-0.0560
K06968	K06968		G210	K01738	0.0339	K03098	-0.0461
K06969	K06969	putative SAM-dependent methyltransferase	G669	K02007	0.0481	K09118	-0.0705
K06973	K06973		G249	K03797	0.0515	K03320	-0.0536
K06975	K06975		G1157	K05516	0.0549	K07341	-0.0796
K06978	K06978		G96	K00274	0.0605	K01304	-0.0569
K06979	K06979		G444	K04750	0.0724	K03821	-0.0504
K06980	K06980		G223	K01439	0.0360	K03092	-0.0299
K06985	K06985		G1582	K09985	0.0758	K03587	-0.0743
K06987	K06987		G18	K01681	0.0561	K09973	-0.0556
K06988	K06988		G490	K04063	0.0575	K07089	-0.0768
K06990	K06990		G216	K04069	0.0854	K07093	-0.0516
K06994	K06994	putative drug exporter of the RND superfamily	G409	K06910	0.0522	K01750	-0.0484
K06995	K06995		G396	K05350	0.0537	K00274	-0.0480
K06996	K06996		G269	K09471	0.0608	K04091	-0.0603
K06997	K06997		G292	K06941	0.0540	K02478	-0.0384
K06998	K06998		G396	K05350	0.0639	K00966	-0.0496
K06999	K06999		G107	K00302	0.0665	K03927	-0.0629
K07000	K07000		G654	K01992	0.0460	K02106	-0.0456
K07001	K07001		G364	K03821	0.0659	K02428	-0.0742
K07002	K07002		G672	K11312	0.0724	K11105	-0.0651
K07003	K07003		G13	K07491	0.0903	K00852	-0.0561
K07004	K07004		G875	K03824	0.0730	K10536	-0.0667
K07005	K07005		G145	K00375	0.0824	K01442	-0.0569
K07006	K07006		G179	K03823	0.0582	K07095	-0.0615
K07007	K07007		G486	K10536	0.0485	K07037	-0.0398

KEGG identifier (gene i)	Name	Description	Correlog group	KEGG identifier (gene j)	w_{ij}	KEGG identifier (gene j')	$w_{ij'}$
K07008	K07008		G150	K07039	0.0481	K00661	-0.0430
K07009	K07009		G70	K04092	0.0407	K03928	-0.0541
K07010	K07010	putative glutamine amidotransferase	G1602	K07147	0.0673	K03926	-0.0548
K07011	K07011		G257	K00795	0.0767	K01114	-0.0632
K07012	K07012		G1604	K07464	0.2543	K07644	-0.0584
K07015	K07015		G162	K06885	0.0575	K03695	-0.0467
K07018	K07018		G459	K05524	0.0376	K06203	-0.0395
K07019	K07019		G1486	K06181	0.0430	K00571	-0.0466
K07020	K07020		G145	K07005	0.0517	K09803	-0.0632
K07021	K07021		G113	K00329	0.0816	K12574	-0.1215
K07023	K07023	putative hydrolases of HD superfamily	G1017	K05521	0.0694	K09803	-0.0651
K07024	K07024		G1213	K03700	0.0771	K03704	-0.0751
K07025	K07025	putative hydrolase of the HAD superfamily	G468	K07101	0.0647	K10543	-0.0620
K07027	K07027		G52	K03284	0.0598	K06223	-0.0794
K07028	K07028		G476	K01552	0.0906	K01188	-0.0568
K07029	K07029		G442	K03192	0.0532	K11312	-0.0543
K07030	K07030		G1174	K08591	0.0546	K02058	-0.0393
K07032	K07032		G28	K07238	0.0607	K07862	-0.0701
K07034	K07034		G489	K01628	0.0694	K00812	-0.0530
K07037	K07037		G197	K08998	0.0709	K05515	-0.0678
K07038	K07038		G471	K03453	0.0624	K07089	-0.0539
K07039	K07039		G150	K03098	0.0567	K06988	-0.0644
K07040	K07040		G1195	K03665	0.0519	K00936	-0.0438
K07043	K07043		G409	K06910	0.0682	K06956	-0.0583
K07046	K07046		G789	K03777	0.0729	K07313	-0.0679
K07047	K07047		G1081	K07150	0.0724	K03893	-0.0485
K07050	K07050		G217	K00657	0.0594	K02484	-0.0500
K07052	K07052		G54	K09017	0.0625	K07263	-0.0594
K07053	K07053		G527	K01673	0.0417	K01440	-0.0367
K07054	K07054		G1017	K03282	0.0541	K01552	-0.0709
K07058	K07058		G1582	K09985	0.0759	K09936	-0.0654
K07062	K07062		G683	K07154	0.0845	K08984	-0.0627
K07067	K07067		G238	K00756	0.0490	K03644	-0.0500
K07071	K07071		G52	K07175	0.0472	K01443	-0.0446
K07075	K07075		G316	K00949	0.0850	K08281	-0.0639
K07080	K07080		G838	K07218	0.0647	K00372	-0.0530
K07082	K07082		G965	K02907	0.0543	K01738	-0.0471
K07084	K07084		G223	K01760	0.0564	K02761	-0.0440
K07085	K07085		G24	K03312	0.0574	K02474	-0.0459
K07088	K07088		G13	K07749	0.0661	K07027	-0.0539
K07089	K07089		G548	K02377	0.0624	K06988	-0.0768
K07090	K07090		G90	K02342	0.0644	K09815	-0.0442
K07091	lptF	lipopolysaccharide export system permease protein	G1186	K11720	0.0441	K01893	-0.0255
K07093	K07093		G396	K03606	0.0587	K09931	-0.0536
K07095	K07095		G1157	K03672	0.0598	K07006	-0.0615
K07098	K07098		G468	K01531	0.0556	K00372	-0.0604
K07101	K07101		G468	K01788	0.0959	K03694	-0.0466
K07102	K07102		G589	K01802	0.0693	K07106	-0.0479

KEGG identifier (gene *i*)	Name	Description	Correlog group	KEGG identifier (gene *j*)	w_{ij}	KEGG identifier (gene *j'*)	$w_{ij'}$
K07104	K07104		G28	K07032	0.0561	K03741	-0.0541
K07106	murQ	N-acetylmuramic acid 6-phosphate etherase	G1649	K09001	0.0653	K06223	-0.0569
K07107	K07107		G174	K00526	0.0501	K00611	-0.0474
K07110	K07110		G508	K01637	0.0518	K06200	-0.0404
K07112	K07112		G16	K01623	0.0703	K02824	-0.0566
K07113	K07113		G269	K09471	0.0463	K00257	-0.0480
K07114	K07114		G230	K01485	0.0675	K03712	-0.0574
K07115	K07115		G409	K06910	0.0857	K05916	-0.0629
K07118	K07118		G30	K07407	0.0802	K03777	-0.0773
K07119	K07119		G444	K06979	0.0558	K12942	-0.0736
K07120	K07120		G1658	K09788	0.0622	K03119	-0.0748
K07121	K07121		G476	K03567	0.0308	K01114	-0.0317
K07122	K07122		G1140	K08310	0.0407	K07300	-0.0497
K07124	K07124		G532	K01991	0.0531	K02029	-0.0537
K07126	K07126		G13	K07497	0.0838	K01795	-0.0664
K07127	K07127		G1056	K03382	0.0794	K07334	-0.0680
K07130	K07130		G351	K01053	0.0697	K03297	-0.0750
K07137	K07137		G0	K00128	0.0618	K07336	-0.0532
K07139	K07139		G80	K00243	0.0512	K06199	-0.0505
K07140	K07140		G351	K01053	0.0636	K01104	-0.0480
K07141	K07141		G1668	K07402	0.1386	K00721	-0.0741
K07145	K07145		G34	K01487	0.0586	K00219	-0.0630
K07146	K07146		G186	K00563	0.0561	K01934	-0.0543
K07147	K07147		G1602	K07010	0.0673	K01514	-0.0465
K07148	K07148		G43	K02472	0.0551	K09931	-0.0525
K07150	K07150		G1081	K07047	0.0724	K00005	-0.0552
K07152	K07152		G1674	K09796	0.0502	K06001	-0.0338
K07153	K07153		G226	K08316	0.0305	K01011	-0.0248
K07154	K07154		G683	K07062	0.0845	K01154	-0.0730
K07156	pcoC		G344	K07245	0.1864	K03712	-0.0445
K07157	K07157		G980	K07223	0.0465	K01804	-0.0404
K07160	K07160		G523	K01664	0.0486	K09922	-0.0458
K07161	K07161		G1102	K03519	0.1114	K00372	-0.0564
K07164	K07164		G1192	K03652	0.0412	K07457	-0.0371
K07166	K07166	ACT domain-containing protein	G358	K09157	0.1985	K07552	-0.0435
K07170	K07170	GAF domain-containing protein	G161	K02919	0.0555	K00563	-0.0499
K07171	K07171		G149	K07473	0.1014	K08994	-0.0734
K07173	luxS	S-ribosylhomocysteine lyase	G393	K01243	0.0621	K01251	-0.0756
K07175	phoH2	PhoH-like ATPase	G52	K03284	0.0476	K00046	-0.0468
K07177	K07177	PDZ domain-containing protein	G409	K06994	0.0479	K00965	-0.0502
K07180	prkA	serine protein kinase	G1688	K09786	0.2049	K09946	-0.0423
K07181	K07181	putative signal transduction protein containing EAL and modified	G773	K03415	0.0768	K07313	-0.0556
K07182	K07182	CBS domain-containing protein	G24	K06001	0.0601	K04767	-0.0776
K07183	nasT	response regulator NasT	G136	K00362	0.0661	K02074	-0.0488
K07184	ygiM	SH3 domain protein	G174	K03548	0.0358	K03478	-0.0338
K07185	tspO	tryptophan-rich sensory protein	G184	K01286	0.0626	K00299	-0.0603
K07213	K07213		G85	K00249	0.0878	K06889	-0.0517
K07218	nosD	nitrous oxidase accessory protein	G838	K04561	0.0819	K03098	-0.0613
K07220	K07220	hypothetical protein	G1036	K03306	0.1765	K09803	-0.0534

KEGG identifier (gene *i*)	Name	Description	Correlog group	KEGG identifier (gene *j*)	w_{ij}	KEGG identifier (gene *j'*)	$w_{ij'}$
K07222	K07222	putative flavoprotein involved in K+ transport	G54	K02510	0.0651	K02919	-0.0575
K07223	K07223	putative iron-dependent peroxidase	G980	K03116	0.0788	K00026	-0.0555
K07226	K07226	hypothetical protein	G15	K07319	0.0586	K02474	-0.0679
K07229	K07229		G1081	K07047	0.0610	K03457	-0.0520
K07234	K07234	uncharacterized protein involved in response to NO	G838	K04561	0.0723	K03665	-0.0400
K07235	tusD, dsrE	tRNA 2-thiouridine synthesizing protein D	G1702	K07236	0.0911	K00001	-0.0277
K07236	tusC, dsrF	tRNA 2-thiouridine synthesizing protein C	G1702	K07237	0.1256	K03672	-0.0370
K07237	tusB, dsrH	tRNA 2-thiouridine synthesizing protein B	G1702	K07236	0.1256	K00694	-0.0486
K07238	TC.ZIP	zinc transporter, ZIP family	G28	K00058	0.0615	K06013	-0.0768
K07239	TC.HME	heavy-metal exporter, HME family	G354	K07787	0.0614	K01551	-0.0437
K07240	chrA	chromate transporter	G470	K06213	0.0577	K00065	-0.0485
K07243	FTR1	high-affinity iron transporter	G1708	K09765	0.0663	K05710	-0.0611
K07245	pcoD	putative copper resistance protein D	G344	K07156	0.1864	K06889	-0.0430
K07246	E1.1.1.83	D-malate dehydrogenase (decarboxylating)	G27	K07247	0.3521	K00598	-0.0395
K07247	E4.1.1.73	tartrate decarboxylase	G27	K07246	0.3521	K00598	-0.0395
K07250	E2.6.1.22, gabT	(S)-3-amino-2-methylpropionate transaminase	G270	K00823	0.2120	K05520	-0.0568
K07258	dacC, dacA, dacD	D-alanyl-D-alanine carboxypeptidase (penicillin-binding protein 5/6)	G13	K01953	0.0489	K06990	-0.0490
K07259	dacB	D-alanyl-D-alanine carboxypeptidase	G1714	K07399	0.0441	K09771	-0.0455
K07260	vanY	D-alanyl-D-alanine carboxypeptidase	G468	K07025	0.0512	K01581	-0.0466
K07261	mepA	penicillin-insensitive murein endopeptidase	G1423	K08990	0.0693	K02303	-0.0345
K07262	pbpG	D-alanyl-D-alanine endopeptidase (penicillin-binding protein 7)	G13	K07483	0.0455	K09930	-0.0427
K07263	pqqL	zinc protease	G200	K01580	0.0603	K06200	-0.0607
K07274	K07274	outer membrane protein	G683	K07154	0.0593	K00005	-0.0525
K07275	ompW	outer membrane protein	G1379	K07735	0.0400	K03313	-0.0612
K07277	yaeT	outer membrane protein	G468	K06925	0.0576	K03313	-0.0578
K07278	ytfM	outer membrane protein	G865	K09800	0.0640	K08998	-0.0451
K07282	pgsA, capA	poly-gamma-glutamate synthesis protein (capsule biosynthesis	G179	K00547	0.0590	K00100	-0.0673
K07284	srtA	sortase A	G1724	K10126	0.0472	K07243	-0.0407
K07287	nlpB	lipoprotein-34	G85	K07213	0.0460	K11747	-0.0369
K07288	tspA	uncharacterized membrane protein	G442	K03192	0.0521	K09967	-0.0368
K07289	asmA	AsmA protein	G606	K01934	0.0595	K03548	-0.0535
K07300	chaA	Ca2+:H+ antiporter	G73	K00842	0.0623	K01607	-0.0593
K07301	yrbG		G525	K01666	0.0568	K03672	-0.0555
K07302	E1.3.99.16A	isoquinoline 1-oxidoreductase, alpha subunit	G1730	K07303	0.2649	K06871	-0.0547

KEGG identifier (gene *i*)	Name	Description	Correlog group	KEGG identifier (gene *j*)	w_{ij}	KEGG identifier (gene *j'*)	$w_{ij'}$
K07303	E1.3.99.16B	isoquinoline 1-oxidoreductase, beta subunit	G1730	K07302	0.2649	K07093	-0.0379
K07304	msrA	peptide-methionine (S)-S-oxide reductase	G279	K07305	0.1125	K12267	-0.1174
K07305	msrB	peptide-methionine (R)-S-oxide reductase	G279	K07304	0.1125	K12267	-0.1071
K07313	pphA	serine/threonine protein phosphatase 1	G13	K07341	0.0715	K07046	-0.0679
K07315	rsbU	sigma-B regulation protein RsbU (phosphoserine phosphatase)	G875	K03824	0.0601	K00949	-0.0522
K07319	yhdJ	putative adenine-specific DNA-methyltransferase	G15	K03427	0.0697	K00557	-0.0866
K07320	prmB	putative adenine-specific DNA-methyltransferase	G26	K03646	0.0319	K09959	-0.0268
K07322	ytfE, scdA	regulator of cell morphogenesis and NO signaling	G217	K07050	0.0532	K01753	-0.0691
K07323	ttg2	putative toluene tolerance protein	G149	K07171	0.0843	K07391	-0.0603
K07334	higA	proteic killer suppression protein	G532	K09803	0.0945	K09939	-0.0728
K07335	bmpA, bmpB, tmpC	basic membrane protein A and related proteins	G715	K02057	0.0934	K04488	-0.0545
K07336	K07336	PKHD-type hydroxylase	G228	K11811	0.0753	K01750	-0.0623
K07337	K07337	hypothetical protein	G749	K02217	0.0540	K07722	-0.0560
K07340	K07340	hypothetical protein	G196	K00598	0.0597	K01555	-0.0451
K07341	doc	death on curing protein	G13	K07491	0.0812	K06975	-0.0796
K07347	fimD	outer membrane usher protein	G1176	K03630	0.0859	K02488	-0.0809
K07386	pepO	putative endopeptidase	G833	K02435	0.0760	K02055	-0.0443
K07390	grxD, GLRX5	monothiol glutaredoxin	G414	K01447	0.0512	K00567	-0.0561
K07391	comM	magnesium chelatase family protein	G1749	K09125	0.0676	K00325	-0.0690
K07393	ECM4, yqjG	putative glutathione S-transferase	G532	K09803	0.0609	K07645	-0.0520
K07395	K07395	putative proteasome-type protease	G5	K01753	0.0664	K00925	-0.0593
K07397	yhfA	putative redox protein	G146	K03439	0.0499	K01665	-0.0520
K07399	resB, ccs1	cytochrome c biogenesis protein	G1714	K07259	0.0441	K11472	-0.0371
K07400	nfuA	Fe/S biogenesis protein NfuA	G1313	K04488	0.0449	K06140	-0.0386
K07402	xdhC	xanthine dehydrogenase accessory factor	G1668	K07141	0.1386	K07088	-0.0446
K07403	nfeD	membrane-bound serine protease (ClpP class)	G649	K09165	0.0849	K00030	-0.0667
K07404	pgl	6-phosphogluconolactonase	G190	K03811	0.0639	K01479	-0.0609
K07407	E3.2.1.22B, galA, rafA	alpha-galactosidase	G30	K12308	0.0803	K00694	-0.0542
K07442	E2.1.1.36, GCD14	tRNA (adenine-N1-)-methyltransferase	G161	K05337	0.0531	K04079	-0.0418
K07443	ybaZ	methylated-DNA-protein-cysteine methyltransferase related protein	G980	K07223	0.0504	K01436	-0.0546
K07444	ycbY	putative N6-adenine-specific DNA methylase	G52	K06223	0.0456	K03321	-0.0441

KEGG identifier (gene *i*)	Name	Description	Correlog group	KEGG identifier (gene *j*)	w_{ij}	KEGG identifier (gene *j'*)	$w_{ij'}$
K07456	mutS2	DNA mismatch repair protein MutS2	G39	K00705	0.0726	K05801	-0.0787
K07457	K07457	endonuclease III related protein	G65	K00176	0.0562	K07668	-0.0495
K07458	vsr	DNA mismatch endonuclease, patch repair protein	G184	K00558	0.1726	K00219	-0.0689
K07460	yraN	putative endonuclease	G89	K03287	0.0623	K02445	-0.0526
K07461	K07461	putative endonuclease	G500	K01620	0.0611	K07185	-0.0596
K07462	recJ	single-stranded-DNA-specific exonuclease	G803	K03555	0.0382	K01439	-0.0322
K07464	K07464	putative RecB family exonuclease	G1604	K07012	0.2543	K03303	-0.0596
K07473	dinJ	DNA-damage-inducible protein J	G149	K07171	0.1014	K01297	-0.0796
K07483	K07483	transposase	G13	K07497	0.1978	K01907	-0.0602
K07491	K07491	putative transposase	G13	K07483	0.0928	K00680	-0.0668
K07497	K07497	putative transposase	G13	K07483	0.1978	K08984	-0.0680
K07506	K07506	AraC family transcriptional regulator	G526	K02068	0.0602	K06183	-0.0678
K07507	mgtC	putative Mg2+ transporter-C (MgtC) family protein	G13	K07483	0.0654	K00616	-0.0505
K07552	bcr	MFS transporter, DHA1 family, bicyclomycin/chloramphenicol	G161	K02919	0.0559	K00567	-0.0578
K07560	dtd	D-tyrosyl-tRNA(Tyr) deacylase	G414	K03574	0.0460	K07075	-0.0427
K07566	SUA5	putative translation factor	G1343	K04564	0.0629	K07095	-0.0467
K07567	tdcF	TdcF protein	G1017	K05995	0.0513	K01669	-0.0555
K07568	queA	S-adenosylmethionine:tRNA ribosyltransferase-isomerase	G965	K02907	0.0711	K05916	-0.0561
K07574	K07574	putative RNA-binding protein containing KH domain	G368	K01118	0.0357	K01531	-0.0358
K07576	K07576	metallo-beta-lactamase family protein	G471	K01537	0.0693	K06223	-0.0583
K07584	K07584	hypothetical protein	G162	K00951	0.0724	K12257	-0.0561
K07588	argK	LAO/AO transport system kinase	G608	K01847	0.1139	K09788	-0.0472
K07636	phoR	two-component system, OmpR family, phosphate regulon sensor	G1499	K07658	0.0859	K00449	-0.0494
K07638	envZ	two-component system, OmpR family, osmolarity sensor histidine	G1785	K07659	0.1127	K00721	-0.0372
K07644	cusS, copS	two-component system, OmpR family, heavy metal sensor histidine	G1786	K07665	0.2486	K07012	-0.0584
K07645	qseC	two-component system, OmpR family, sensor histidine kinase QseC	G490	K06075	0.0649	K00249	-0.0546
K07646	kdpD	two-component system, OmpR family, sensor histidine kinase KdpD	G472	K01547	0.1447	K01953	-0.0359
K07649	tctE	two-component system, OmpR family, sensor histidine kinase TctE	G1789	K07795	0.0782	K07171	-0.0474
K07652	vicK	two-component system, OmpR family, sensor histidine kinase VicK	G1790	K07668	0.0707	K07240	-0.0372

KEGG identifier (gene *i*)	Name	Description	Correlog group	KEGG identifier (gene *j*)	w_{ij}	KEGG identifier (gene *j'*)	$w_{ij'}$
K07657	phoB	two-component system, OmpR family, phosphate regulon response	G1499	K07636	0.0598	K07115	-0.0619
K07658	phoB1, phoP	two-component system, OmpR family, alkaline phosphatase synthesis	G1499	K07636	0.0859	K06901	-0.0473
K07659	ompR	two-component system, OmpR family, phosphate regulon response	G1785	K07638	0.1127	K00721	-0.0446
K07662	cpxR	two-component system, OmpR family, response regulator CpxR	G525	K01666	0.0612	K09165	-0.0507
K07665	cusR	two-component system, OmpR family, copper resistance phosphate	G1786	K07644	0.2486	K01520	-0.0490
K07667	kdpE	two-component system, OmpR family, KDP operon response regulator	G472	K07646	0.1293	K00782	-0.0459
K07668	vicR	two-component system, OmpR family, response regulator VicR	G1790	K07652	0.0707	K07457	-0.0495
K07678	barA	two-component system, NarL family, sensor histidine kinase BarA	G13	K06149	0.0422	K08987	-0.0305
K07684	narL	two-component system, NarL family, nitrate/nitrite response	G143	K00373	0.0665	K07222	-0.0561
K07708	glnL, ntrB	two-component system, NtrC family, nitrogen regulation sensor	G617	K07712	0.0586	K08978	-0.0229
K07712	glnG, ntrC	two-component system, NtrC family, nitrogen regulation response	G617	K07708	0.0586	K04757	-0.0288
K07722	nikR	CopG family transcriptional regulator, nickel-responsive regulator	G303	K03620	0.0737	K05710	-0.0645
K07727	K07727	putative transcriptional regulator	G22	K00661	0.0780	K00012	-0.0522
K07734	paiB	transcriptional regulator	G425	K01426	0.0527	K00219	-0.0621
K07735	algH	putative transcriptional regulator	G1379	K06894	0.0516	K07154	-0.0606
K07736	K07736	CarD family transcriptional regulator	G1379	K06894	0.0433	K07347	-0.0453
K07737	K07737	putative transcriptional regulator	G221	K00666	0.0673	K03328	-0.0716
K07738	nrdR	transcriptional repressor NrdR	G976	K03091	0.0513	K01529	-0.0497
K07742	K07742	hypothetical protein	G1809	K12574	0.0584	K07021	-0.0523
K07749	E2.8.3.16	formyl-CoA transferase	G13	K07483	0.0730	K01259	-0.0662
K07778	desK	two-component system, NarL family, sensor histidine kinase DesK	G190	K00571	0.0751	K03574	-0.0739
K07787	cusA	Cu(I)/Ag(I) efflux system membrane protein CusA	G354	K07798	0.2707	K09959	-0.0485
K07788	mdtB	RND superfamily, multidrug transport protein MdtB	G1813	K07789	0.1656	K06876	-0.0342
K07789	mdtC	RND superfamily, multidrug transport protein MdtC	G1813	K07788	0.1656	K04561	-0.0353
K07795	tctC	putative tricarboxylic transport membrane protein	G1789	K07649	0.0782	K03724	-0.0552

KEGG identifier (gene *i*)	Name	Description	Correlog group	KEGG identifier (gene *j*)	w_{ij}	KEGG identifier (gene *j'*)	$w_{ij'}$
K07798	cusB	Cu(I)/Ag(I) efflux system membrane protein CusB	G354	K07787	0.2707	K07491	-0.0508
K07799	mdtA	putative multidrug efflux transporter MdtA	G1813	K07789	0.1209	K05939	-0.0500
K07814	rpfG	putative two-component system response regulator	G162	K08987	0.0648	K01816	-0.0580
K07862	sstT	serine/threonine transporter	G1819	K11928	0.0657	K07032	-0.0701
K08084	fimT	type IV fimbrial biogenesis protein FimT	G920	K02672	0.0758	K09936	-0.0437
K08156	araJ	MFS transporter, DHA1 family, arabinose polymer transporter	G636	K01938	0.0747	K03496	-0.0642
K08218	ampG	MFS transporter, PAT family, beta-lactamase induction signal	G1602	K09181	0.0507	K09928	-0.0434
K08223	fsr	MFS transporter, FSR family, fosmidomycin resistance protein	G444	K01476	0.0566	K00632	-0.0533
K08224	ynfM	MFS transporter, YNFM family, putative membrane transport protein	G50	K00135	0.0610	K00363	-0.0568
K08234	yaeR	glyoxylase I family protein	G172	K00520	0.0614	K01239	-0.0586
K08281	pncA	pyrazinamidase	G244	K01440	0.2300	K07075	-0.0639
K08289	purT	phosphoribosylglycinamide formyltransferase 2	G1554	K06915	0.0540	K07473	-0.0497
K08296	sixA	phosphohistidine phosphatase	G489	K01628	0.0434	K03453	-0.0479
K08300	rne	ribonuclease E	G223	K06980	0.0343	K00564	-0.0325
K08301	rng, cafA	ribonuclease G	G241	K03602	0.1013	K00832	-0.0509
K08303	K08303	putative protease	G200	K01478	0.0626	K11811	-0.0491
K08304	mltA	membrane-bound lytic murein transglycosylase A	G525	K07301	0.0481	K01524	-0.0454
K08305	mltB	membrane-bound lytic murein transglycosylase B	G525	K01666	0.0575	K01916	-0.0556
K08307	mltD, dniR	membrane-bound lytic murein transglycosylase D	G750	K03589	0.0312	K06949	-0.0303
K08309	slt	soluble lytic murein transglycosylase	G200	K00611	0.0702	K01139	-0.0722
K08310	ntpA, nudB	dATP pyrophosphohydrolase	G1140	K03577	0.0457	K07407	-0.0395
K08311	nudH	putative (di)nucleoside polyphosphate hydrolase	G163	K00449	0.0343	K03744	-0.0319
K08312	nudE	ADP-ribose diphosphatase	G30	K03556	0.0368	K07458	-0.0305
K08316	rsmD	ribosomal RNA small subunit methyltransferase D	G226	K05589	0.0366	K09861	-0.0274
K08369	ydjE	MFS transporter, putative metabolite:H+ symporter	G13	K07483	0.0713	K02474	-0.0527
K08483	PTS-EI.PTSI, ptsI	phosphotransferase system, enzyme I, PtsI	G8	K00627	0.0679	K01286	-0.0442
K08484	PTS-EI.PTSP, ptsP	phosphotransferase system, enzyme I, PtsP	G1554	K11940	0.0267	K03795	-0.0284
K08591	ygiH	putative membrane protein	G1174	K03621	0.1436	K08998	-0.0360
K08602	pepF, pepB	oligoendopeptidase F	G149	K01299	0.0482	K05592	-0.0474
K08641	vanX	D-alanyl-D-alanine dipeptidase	G194	K03795	0.0686	K01295	-0.0795

KEGG identifier (gene *i*)	Name	Description	Correlog group	KEGG identifier (gene *j*)	w_{ij}	KEGG identifier (gene *j'*)	$w_{ij'}$
K08680	menH	2-succinyl-6-hydroxy-2,4-cyclohexadiene-1-carboxylate synthase	G882	K02552	0.0932	K09861	-0.0349
K08681	pdxT, pdx2	glutamine amidotransferase	G1509	K06215	0.1634	K06213	-0.0464
K08738	CYC	cytochrome c	G269	K09471	0.0463	K02058	-0.0391
K08884	K08884	serine/threonine protein kinase, bacterial	G204	K06873	0.0648	K03700	-0.0722
K08963	mtnA	methylthioribose-1-phosphate isomerase	G1850	K08967	0.1225	K01243	-0.0463
K08967	mtnD, mtnZ, ADI1	1,2-dihydroxy-3-keto-5-methylthiopentene dioxygenase	G1850	K08963	0.1225	K09771	-0.0474
K08972	K08972	putative membrane protein	G1089	K03469	0.0587	K07182	-0.0418
K08973	K08973	putative membrane protein	G90	K03724	0.0418	K00231	-0.0568
K08974	K08974	putative membrane protein	G480	K01572	0.0510	K03925	-0.0614
K08978	K08978	putative membrane protein	G471	K03453	0.0840	K02069	-0.0641
K08981	K08981	putative membrane protein	G1856	K09167	0.2002	K01753	-0.0426
K08984	yjdF	putative membrane protein	G11	K06076	0.0579	K07497	-0.0680
K08987	K08987	putative membrane protein	G162	K01930	0.0671	K01190	-0.0753
K08990	ycjF	putative membrane protein	G1423	K06918	0.0821	K01709	-0.0396
K08994	yneE	putative membrane protein	G73	K00917	0.0679	K07171	-0.0734
K08998	K08998	hypothetical protein	G197	K00599	0.0828	K01159	-0.0774
K08999	K08999	hypothetical protein	G468	K06925	0.0477	K01207	-0.0398
K09001	anmK	anhydro-N-acetylmuramic acid kinase	G1649	K07106	0.0653	K07080	-0.0452
K09005	K09005	hypothetical protein	G980	K07157	0.0460	K07005	-0.0487
K09007	K09007	hypothetical protein	G396	K03606	0.0509	K01495	-0.0872
K09008	K09008	hypothetical protein	G53	K00286	0.0537	K03570	-0.0566
K09013	sufC	Fe-S cluster assembly ATP-binding protein	G69	K09014	0.1693	K05349	-0.0497
K09014	sufB	Fe-S cluster assembly protein SufB	G69	K09013	0.1693	K01916	-0.0347
K09015	sufD	Fe-S cluster assembly protein SufD	G69	K09014	0.1064	K01467	-0.0467
K09017	rutR	TetR/AcrR family transcriptional regulator	G54	K07222	0.0648	K02068	-0.0849
K09117	K09117	hypothetical protein	G636	K01938	0.0526	K00074	-0.0538
K09118	K09118	hypothetical protein	G235	K00966	0.0471	K06969	-0.0705
K09121	K09121	hypothetical protein	G1525	K06898	0.2243	K01495	-0.0379
K09125	K09125	hypothetical protein	G1749	K07391	0.0676	K03284	-0.0660
K09131	K09131	hypothetical protein	G489	K07034	0.0499	K09165	-0.0600
K09134	K09134	hypothetical protein	G373	K05349	0.0562	K06867	-0.0587
K09136	K09136	hypothetical protein	G190	K01488	0.0569	K01725	-0.0559
K09157	K09157	hypothetical protein	G358	K07166	0.1985	K02428	-0.0481
K09158	K09158	hypothetical protein	G43	K01791	0.0348	K00451	-0.0306
K09159	K09159	hypothetical protein	G1166	K05540	0.0817	K00100	-0.0670
K09160	K09160	hypothetical protein	G188	K02428	0.0448	K07102	-0.0312
K09162	K09162	hypothetical protein	G1213	K07024	0.0272	K00228	-0.0312
K09165	K09165	hypothetical protein	G649	K07403	0.0849	K09131	-0.0600
K09167	K09167	hypothetical protein	G1856	K08981	0.2002	K06871	-0.0485
K09181	yfiQ	hypothetical protein	G1602	K07147	0.0559	K09017	-0.0611
K09456	aidB	putative acyl-CoA dehydrogenase	G30	K05710	0.0568	K06988	-0.0479

KEGG identifier (gene *i*)	Name	Description	Correlog group	KEGG identifier (gene *j*)	w_{ij}	KEGG identifier (gene *j'*)	$w_{ij'}$
K09457	K09457, queF	7-cyano-7-deazaguanine reductase	G559	K06879	0.0321	K03287	-0.0281
K09471	puuB, ordL	gamma-glutamylputrescine oxidase	G269	K09472	0.0897	K11811	-0.0584
K09472	puuC, aldH	gamma-glutamyl-gamma-aminobutyraldehyde dehydrogenase	G269	K09471	0.0897	K00694	-0.0555
K09685	K09685, purR	purine operon repressor	G1790	K07652	0.0498	K09765	-0.0361
K09686	ABC-2.AB.P	antibiotic transport system permease protein	G1891	K09687	0.0907	K00949	-0.0615
K09687	ABC-2.AB.A	antibiotic transport system ATP-binding protein	G1891	K09686	0.0907	K01624	-0.0527
K09690	ABC-2.LPSE.P	lipopolysaccharide transport system permease protein	G1893	K09691	0.3484	K01190	-0.0445
K09691	ABC-2.LPSE.A	lipopolysaccharide transport system ATP-binding protein	G1893	K09690	0.3484	K10536	-0.0446
K09698	gltX1	nondiscriminating glutamyl-tRNA synthetase	G397	K05592	0.0658	K06948	-0.0688
K09699	E2.3.1.168, bkdB	2-oxoisovalerate dehydrogenase E2 component (dihydrolipoyl	G61	K00166	0.1784	K09967	-0.0338
K09701	K09701	hypothetical protein	G150	K03098	0.1059	K03534	-0.0596
K09705	K09705	hypothetical protein	G672	K11312	0.0572	K00974	-0.0594
K09749	K09749	hypothetical protein	G24	K03409	0.0483	K02919	-0.0413
K09760	rmuC	DNA recombination protein RmuC	G1129	K03704	0.0715	K07027	-0.0688
K09762	K09762	hypothetical protein	G988	K03151	0.0679	K03700	-0.0620
K09763	K09763	hypothetical protein	G146	K00937	0.0408	K07043	-0.0390
K09764	K09764	hypothetical protein	G460	K01512	0.0474	K03205	-0.0438
K09765	K09765	hypothetical protein	G1708	K07243	0.0663	K01485	-0.0554
K09767	K09767	hypothetical protein	G1017	K03315	0.0424	K07024	-0.0440
K09768	K09768	hypothetical protein	G1497	K06193	0.0625	K01512	-0.0456
K09769	K09769	hypothetical protein	G1907	K09798	0.0489	K01754	-0.0436
K09771	K09771	hypothetical protein	G475	K01551	0.0692	K01532	-0.0522
K09772	K09772	hypothetical protein	G396	K03711	0.0484	K12257	-0.0427
K09773	K09773	hypothetical protein	G340	K01006	0.0599	K06990	-0.0331
K09774	lptA	lipopolysaccharide export system protein LptA	G858	K02474	0.0337	K07150	-0.0297
K09777	K09777	hypothetical protein	G471	K01537	0.0425	K03312	-0.0398
K09778	K09778	hypothetical protein	G876	K09949	0.0599	K02567	-0.0384
K09780	K09780	hypothetical protein	G269	K09472	0.0614	K03827	-0.0574
K09781	K09781	hypothetical protein	G471	K03453	0.0714	K06988	-0.0646
K09786	K09786	hypothetical protein	G1688	K07180	0.2049	K09967	-0.0323
K09787	K09787	hypothetical protein	G616	K01914	0.0545	K00882	-0.0534
K09788	K09788	hypothetical protein	G1658	K09975	0.0683	K07012	-0.0488
K09790	K09790	hypothetical protein	G1521	K06518	0.0646	K01423	-0.0499
K09791	K09791	hypothetical protein	G162	K01091	0.0500	K05966	-0.0471
K09792	K09792	hypothetical protein	G152	K00404	0.1140	K03620	-0.0445
K09793	K09793	hypothetical protein	G13	K07341	0.0561	K02919	-0.0567
K09794	K09794	hypothetical protein	G13	K07749	0.0653	K07334	-0.0588
K09796	K09796	hypothetical protein	G1674	K07152	0.0502	K02474	-0.0471
K09798	K09798	hypothetical protein	G1907	K09769	0.0489	K03699	-0.0430

KEGG identifier (gene *i*)	Name	Description	Correlog group	KEGG identifier (gene *j*)	w_{ij}	KEGG identifier (gene *j'*)	$w_{ij'}$
K09800	K09800	hypothetical protein	G865	K07278	0.0640	K00759	-0.0474
K09801	K09801	hypothetical protein	G1171	K03616	0.0594	K09888	-0.0282
K09803	K09803	hypothetical protein	G532	K07334	0.0945	K07023	-0.0651
K09806	K09806	hypothetical protein	G92	K03446	0.0599	K06153	-0.0519
K09807	K09807	hypothetical protein	G351	K07130	0.0497	K07011	-0.0503
K09808	ABC.LPT.P, lolC, lolE	lipoprotein-releasing system permease protein	G36	K06142	0.0470	K07003	-0.0438
K09810	ABC.LPT.A , lolD	lipoprotein-releasing system ATP-binding protein	G327	K01840	0.0870	K02003	-0.0676
K09811	ftsX	cell division transport system permease protein	G1933	K09812	0.1721	K00020	-0.0355
K09812	ftsE	cell division transport system ATP-binding protein	G1933	K09811	0.1721	K00261	-0.0486
K09815	znuA	zinc transport system substrate-binding protein	G863	K09817	0.1657	K07657	-0.0483
K09816	znuB	zinc transport system permease protein	G863	K09817	0.1850	K07347	-0.0474
K09817	znuC	zinc transport system ATP-binding protein	G863	K09816	0.1850	K01685	-0.0374
K09818	ABC.MN.S	manganese/iron transport system substrate-binding protein	G1938	K09820	0.1931	K07005	-0.0541
K09819	ABC.MN.P	manganese/iron transport system permease protein	G1938	K09820	0.3393	K03973	-0.0729
K09820	ABC.MN.A	manganese/iron transport system ATP-binding protein	G1938	K09819	0.3393	K07684	-0.0424
K09823	zur	Fur family transcriptional regulator, zinc uptake regulator	G221	K03297	0.0374	K02217	-0.0364
K09825	perR	Fur family transcriptional regulator, peroxide stress response	G1425	K05808	0.0690	K00978	-0.0511
K09857	K09857	hypothetical protein	G1278	K06192	0.2135	K07814	-0.0409
K09858	K09858	SEC-C motif domain protein	G54	K00151	0.0521	K01739	-0.0428
K09861	K09861	hypothetical protein	G188	K00567	0.0655	K05794	-0.0575
K09862	K09862	hypothetical protein	G161	K00428	0.0560	K07171	-0.0377
K09882	cobS	cobaltochelatase CobS	G184	K07458	0.0569	K00952	-0.0471
K09888	K09888	hypothetical protein	G437	K09924	0.0600	K07120	-0.0579
K09889	K09889	hypothetical protein	G54	K09930	0.0310	K00957	-0.0252
K09895	K09895	hypothetical protein	G36	K03629	0.0259	K03602	-0.0174
K09898	K09898	hypothetical protein	G11	K06899	0.0384	K07080	-0.0399
K09902	K09902	hypothetical protein	G36	K09895	0.0249	K01077	-0.0181
K09908	K09908	hypothetical protein	G442	K07288	0.0338	K03747	-0.0298
K09913	K09913	hypothetical protein	G217	K00657	0.0489	K01912	-0.0440
K09914	K09914	putative lipoprotein	G54	K02509	0.0561	K01734	-0.0485
K09915	K09915	hypothetical protein	G683	K02022	0.0525	K07567	-0.0370
K09919	K09919	hypothetical protein	G344	K01011	0.0385	K03737	-0.0401
K09921	K09921	hypothetical protein	G327	K01809	0.0349	K07098	-0.0279
K09922	K09922	hypothetical protein	G1521	K09790	0.0643	K03893	-0.0767
K09923	K09923	hypothetical protein	G672	K11312	0.0401	K01685	-0.0501
K09924	K09924	hypothetical protein	G437	K09888	0.0600	K03409	-0.0520
K09927	K09927	hypothetical protein	G13	K07483	0.0633	K01659	-0.0526
K09928	K09928	hypothetical protein	G327	K00971	0.0563	K11065	-0.0493
K09930	K09930	hypothetical protein	G54	K00151	0.0652	K02624	-0.0624
K09931	K09931	hypothetical protein	G250	K00772	0.0819	K01055	-0.0537

KEGG identifier (gene *i*)	Name	Description	Correlog group	KEGG identifier (gene *j*)	w_{ij}	KEGG identifier (gene *j'*)	$w_{ij'}$
K09933	K09933	hypothetical protein	G649	K01975	0.0402	K00965	-0.0474
K09936	K09936	hypothetical protein	G55	K03827	0.0776	K07058	-0.0654
K09937	K09937	hypothetical protein	G437	K09924	0.0529	K06871	-0.0664
K09939	K09939	hypothetical protein	G161	K02919	0.0677	K07334	-0.0728
K09941	K09941	hypothetical protein	G672	K03554	0.0374	K03192	-0.0400
K09946	K09946	hypothetical protein	G1554	K12373	0.0686	K01532	-0.0630
K09949	K09949	hypothetical protein	G876	K09778	0.0599	K03269	-0.0540
K09954	K09954	hypothetical protein	G230	K07114	0.0434	K01953	-0.0395
K09958	K09958	hypothetical protein	G525	K01725	0.0889	K09937	-0.0656
K09959	K09959	hypothetical protein	G96	K00274	0.0733	K03404	-0.0505
K09967	K09967	hypothetical protein	G556	K04565	0.0736	K08994	-0.0698
K09969	aapJ, bztA	general L-amino acid transport system substrate-binding protein	G1977	K09971	0.1650	K07011	-0.0445
K09970	aapQ, bztB	general L-amino acid transport system permease protein	G1977	K09971	0.1948	K00261	-0.0317
K09971	aapM, bztC	general L-amino acid transport system permease protein	G1977	K09970	0.1948	K01055	-0.0371
K09972	aapP, bztD	general L-amino acid transport system ATP-binding protein	G1977	K09970	0.1430	K07127	-0.0437
K09973	K09973	hypothetical protein	G96	K09959	0.0474	K06987	-0.0556
K09975	K09975	hypothetical protein	G1658	K09788	0.0683	K04719	-0.0504
K09985	K09985	hypothetical protein	G1582	K07058	0.0759	K01710	-0.0684
K09986	K09986	hypothetical protein	G93	K00263	0.0331	K01737	-0.0365
K09987	K09987	hypothetical protein	G1582	K09985	0.0289	K04567	-0.0219
K09989	K09989	hypothetical protein	G54	K01744	0.0538	K05794	-0.0451
K10001	gltI	glutamate/aspartate transport system substrate-binding protein	G1987	K10004	0.1223	K07458	-0.0357
K10002	gltK	glutamate/aspartate transport system permease protein	G1987	K10003	0.1331	K03203	-0.0335
K10003	gltJ	glutamate/aspartate transport system permease protein	G1987	K10002	0.1331	K01259	-0.0384
K10004	gltL	glutamate/aspartate transport system ATP-binding protein	G1987	K10003	0.1278	K06016	-0.0321
K10026	queE, ykvL, ygcF	queuosine biosynthesis protein QueE	G559	K06920	0.0643	K05591	-0.0463
K10041	ABC.GLN1.A	putative glutamine transport system ATP-binding protein	G715	K07335	0.0599	K06201	-0.0707
K10108	malE	maltose/maltodextrin transport system substrate-binding protein	G381	K10109	0.2185	K07497	-0.0525
K10109	malF	maltose/maltodextrin transport system permease protein	G381	K10110	0.2418	K00249	-0.0383
K10110	malG	maltose/maltodextrin transport system permease protein	G381	K10109	0.2418	K03449	-0.0342
K10112	msmX, msmK	maltose/maltodextrin transport system ATP-binding protein	G381	K10109	0.0845	K01190	-0.0586
K10125	dctB	two-component system, NtrC family, C4-dicarboxylate transport sensor	G1724	K10126	0.2375	K07154	-0.0408
K10126	dctD	two-component system, NtrC family, C4-dicarboxylate transport	G1724	K10125	0.2375	K00040	-0.0484
K10439	rbsB	ribose transport system substrate-binding protein	G1999	K10441	0.1875	K07507	-0.0447

KEGG identifier (gene *i*)	Name	Description	Correlog group	KEGG identifier (gene *j*)	w_{ij}	KEGG identifier (gene *j'*)	$w_{ij'}$
K10440	rbsC	ribose transport system permease protein	G1999	K10441	0.2348	K01479	-0.0459
K10441	rbsA	ribose transport system ATP-binding protein	G1999	K10440	0.2348	K09765	-0.0418
K10536	E3.5.3.12	agmatine deiminase	G486	K12251	0.1898	K01480	-0.0880
K10543	xylF	D-xylose transport system substrate-binding protein	G285	K01805	0.0802	K01761	-0.0676
K10563	mutM, fpg	formamidopyrimidine-DNA glycosylase	G1410	K06895	0.0867	K05540	-0.0658
K10680	nemA	N-ethylmaleimide reductase	G269	K09472	0.0640	K01709	-0.0608
K10763	hda	DnaA-homolog protein	G373	K03925	0.0273	K05794	-0.0217
K10764	metZ	O-succinylhomoserine sulfhydrylase	G223	K05499	0.0375	K07005	-0.0362
K10778	ADA	AraC family transcriptional regulator, regulatory protein of	G395	K01247	0.1036	K01725	-0.0447
K10805	tesB	acyl-CoA thioesterase II	G358	K03426	0.0397	K07576	-0.0476
K10806	yciA	acyl-CoA thioesterase YciA	G316	K07075	0.0549	K00759	-0.0405
K10907	K10907	aminotransferase	G486	K10536	0.0482	K12942	-0.0387
K10914	crp	CRP/FNR family transcriptional regulator, cyclic AMP receptor	G980	K07443	0.0337	K02433	-0.0370
K10947	padR	PadR family transcriptional regulator, regulatory protein PadR	G239	K00805	0.0660	K02068	-0.0744
K10979	ku	DNA end-binding protein Ku	G648	K01971	0.1686	K07814	-0.0502
K11065	tpx	thiol peroxidase, atypical 2-Cys peroxiredoxin	G425	K01532	0.0807	K01514	-0.0544
K11068	hlyIII	hemolysin III	G39	K07456	0.0654	K05801	-0.0545
K11069	potD	spermidine/putrescine transport system substrate-binding protein	G2017	K11071	0.2073	K00756	-0.0374
K11070	potC	spermidine/putrescine transport system permease protein	G2017	K11071	0.2338	K02053	-0.0708
K11071	potB	spermidine/putrescine transport system permease protein	G2017	K11070	0.2338	K02054	-0.0664
K11072	potA	spermidine/putrescine transport system ATP-binding protein	G2017	K11071	0.1433	K01779	-0.0539
K11073	potF	putrescine transport system substrate-binding protein	G2021	K11075	0.1362	K07050	-0.0363
K11074	potI	putrescine transport system permease protein	G2021	K11076	0.1425	K01993	-0.0362
K11075	potH	putrescine transport system permease protein	G2021	K11074	0.1400	K07050	-0.0323
K11076	potG	putrescine transport system ATP-binding protein	G2021	K11074	0.1425	K00660	-0.0374
K11085	msbA	ATP-binding cassette, subfamily B, bacterial MsbA	G339	K00998	0.0563	K01776	-0.0496
K11103	dctA	aerobic C4-dicarboxylate transport protein	G200	K03761	0.0510	K06925	-0.0514
K11105	cvrA, nhaP2	cell volume regulation protein A	G301	K01433	0.0719	K07002	-0.0651
K11145	K11145	ribonuclease III family protein	G459	K06183	0.0341	K12506	-0.0333
K11175	purN	phosphoribosylglycinamide formyltransferase 1	G488	K01589	0.0592	K01575	-0.0535

KEGG identifier (gene *i*)	Name	Description	Correlog group	KEGG identifier (gene *j*)	w_{ij}	KEGG identifier (gene *j'*)	$w_{ij'}$
K11177	yagR	xanthine dehydrogenase YagR molybdenum-binding subunit	G1668	K07141	0.1024	K01666	-0.0662
K11179	tusE, dsrC	tRNA 2-thiouridine synthesizing protein E	G1702	K07235	0.0574	K01858	-0.0356
K11183	PTS-HPR.FRUB, fruB, fpr	phosphocarrier protein FPr	G867	K02768	0.1486	K03436	-0.0465
K11189	PTS-HPR	phosphocarrier protein	G871	K02503	0.0732	K09134	-0.0574
K11209	yghU	GST-like protein	G38	K03929	0.0594	K06518	-0.0606
K11312	K11312	cupin 2 domain-containing protein	G672	K02010	0.0745	K01139	-0.0672
K11472	glcE	glycolate oxidase FAD binding subunit	G40	K11473	0.1348	K03777	-0.0386
K11473	glcF	glycolate oxidase iron-sulfur subunit	G40	K11472	0.1348	K09930	-0.0384
K11717	sufS	cysteine desulfurase	G69	K09014	0.1242	K01081	-0.0630
K11719	lptC	lipopolysaccharide export system protein LptC	G1186	K07091	0.0300	K00970	-0.0205
K11720	lptG	lipopolysaccharide export system permease protein	G1186	K07091	0.0441	K06904	-0.0234
K11741	sugE	quaternary ammonium compound-resistance protein SugE	G1472	K06140	0.0791	K02429	-0.0719
K11747	kefB	glutathione-regulated potassium-efflux system protein KefB	G11	K06076	0.0580	K05993	-0.0656
K11749	rseP	regulator of sigma E protease	G830	K06202	0.0830	K01417	-0.0922
K11752	ribD	diaminohydroxyphosphoribosylamino pyrimidine deaminase	G255	K00793	0.1405	K05966	-0.0399
K11754	folC	dihydrofolate synthase	G113	K07021	0.0758	K01930	-0.1534
K11755	hisIE	phosphoribosyl-ATP pyrophosphohydrolase	G361	K01089	0.0570	K01496	-0.1079
K11811	arsH	arsenical resistance protein ArsH	G228	K07336	0.0753	K02509	-0.0713
K11891	impL, vasK, icmF	type VI secretion system protein ImpL	G2048	K11900	0.1332	K01286	-0.0346
K11892	impK, ompA, vasF, dotU	type VI secretion system protein ImpK	G2049	K11893	0.1070	K00219	-0.0243
K11893	impJ, vasE	type VI secretion system protein ImpJ	G2049	K11901	0.1149	K03317	-0.0225
K11895	impH, vasB	type VI secretion system protein ImpH	G2051	K11896	0.1274	K05782	-0.0225
K11896	impG, vasA	type VI secretion system protein ImpG	G2051	K11895	0.1274	K07798	-0.0270
K11900	impC	type VI secretion system protein ImpC	G2048	K11891	0.1332	K07334	-0.0355
K11901	impB	type VI secretion system protein ImpB	G2049	K11893	0.1149	K00025	-0.0234
K11903	K11903, hcp	type VI secretion system secreted protein Hcp	G2049	K11892	0.1024	K01501	-0.0433
K11906	vasD, lip	type VI secretion system protein VasD	G2051	K11907	0.0790	K01854	-0.0466
K11907	vasG, clpV	type VI secretion system protein VasG	G2051	K11895	0.0996	K07722	-0.0339

KEGG identifier (gene *i*)	Name	Description	Correlog group	KEGG identifier (gene *j*)	w_{ij}	KEGG identifier (gene *j'*)	$w_{ij'}$
K11927	rhlE	ATP-dependent RNA helicase RhlE	G149	K07473	0.0671	K11209	-0.0469
K11928	putP	sodium/proline symporter	G1819	K07862	0.0657	K00077	-0.0466
K11940	hspQ	heat shock protein HspQ	G1554	K09946	0.0335	K03181	-0.0373
K12251	aguB	N-carbamoylputrescine amidase	G486	K10536	0.1898	K01480	-0.0741
K12257	secDF	SecD/SecF fusion protein	G21	K00046	0.0393	K03072	-0.1074
K12262	cybB	cytochrome b561	G143	K07684	0.0621	K00680	-0.0561
K12267	msrAB	peptide methionine sulfoxide reductase msrA/msrB	G616	K01914	0.0612	K07304	-0.1174
K12297	rlmL	ribosomal RNA large subunit methyltransferase L	G1141	K03684	0.0349	K07444	-0.0425
K12308	bgaB, lacA	beta-galactosidase	G30	K01190	0.0893	K03827	-0.0795
K12339	cysM	cysteine synthase B	G150	K00389	0.0566	K01738	-0.0432
K12340	tolC	outer membrane channel protein TolC	G1030	K03296	0.0882	K03287	-0.0655
K12368	dppA	dipeptide transport system substrate-binding protein	G2069	K12372	0.1296	K03386	-0.0418
K12369	dppB	dipeptide transport system permease protein	G2069	K12370	0.1128	K01436	-0.0442
K12370	dppC	dipeptide transport system permease protein	G2069	K12371	0.1338	K00154	-0.0363
K12371	dppD	dipeptide transport system ATP-binding protein	G2069	K12372	0.1339	K06904	-0.0281
K12372	dppF	dipeptide transport system ATP-binding protein	G2069	K12371	0.1339	K02670	-0.0316
K12373	HEX	beta-hexosaminidase	G1554	K09946	0.0686	K01239	-0.0599
K12410	npdA	NAD-dependent deacetylase	G230	K07114	0.0498	K04768	-0.0529
K12506	ispDF	2-C-methyl-D-erythritol 4-phosphate cytidylyltransferase	G36	K03526	0.0553	K01770	-0.1349
K12510	tadB	tight adherence protein B	G772	K12511	0.1645	K01501	-0.0362
K12511	tadC	tight adherence protein C	G772	K12510	0.1645	K03439	-0.0400
K12524	thrA	bifunctional aspartokinase/homoserine dehydrogenase 1	G223	K00674	0.0410	K00003	-0.0586
K12573	rnr, vacB	ribonuclease R	G200	K08309	0.0486	K07101	-0.0460
K12574	rnj	ribonuclease J	G1809	K07742	0.0584	K07021	-0.1215
K12942	abgT	aminobenzoyl-glutamate transport protein	G1521	K09790	0.0543	K07119	-0.0736
K12972	ghrA	gyoxylate/hydroxypyruvate reductase A	G344	K01011	0.0541	K01236	-0.0477
K13038	coaBC, dfp	phosphopantothenoylcysteine decarboxylase	G292	K01776	0.0773	K09806	-0.0482
