# Supplementary material for: Genetic Co-Occurrence Network across Sequenced Microbes
Source: PLoS Comput Biol. 2011 Dec 29;7(12):e1002340. doi: 10.1371/journal.pcbi.1002340 (PMC3248385; doi:10.1371/journal.pcbi.1002340)
Supplement: Table S4 — Pairs of functions highly likely to be assigned to the same correlog groups (P<10−3; see Methods). The 1st and 2nd columns contain the names of functions in each function pair, the 3rd column records the fraction of correlog groups having both functions in the pair among correlog groups having either of functions in the pair, and the 4th column is for the corresponding P values. (PDF) [file pcbi.1002340.s007.pdf]

Table S4. Pairs of functions highly likely to be assigned to the same correlog groups ($P < 10^{-3}$; see Methods). The 1st and 2nd columns contain the names of functions in each function pair, the 3rd column records the fraction of correlog groups having both functions in the pair among correlog groups having either of functions in the pair, and the 4th column is for the corresponding P values.

Functional category	Functional category	Overlapping ratio	P value
Membrane Transport	Membrane Transport	0.647	$< 10^{-4}$
Cell Motility	Cell Motility	0.633	$< 10^{-4}$
Carbohydrate Metabolism	Carbohydrate Metabolism	0.626	$< 10^{-4}$
Amino Acid Metabolism	Amino Acid Metabolism	0.589	$< 10^{-4}$
Energy Metabolism	Energy Metabolism	0.522	$< 10^{-4}$
Metabolism of Cofactors and Vitamins	Metabolism of Cofactors and Vitamins	0.500	$< 10^{-4}$
Biosynthesis of Polyketides and Terpenoids	Biosynthesis of Polyketides and Terpenoids	0.467	$< 10^{-4}$
Signal Transduction	Signal Transduction	0.450	$< 10^{-4}$
Replication and Repair	Replication and Repair	0.418	$< 10^{-4}$
Xenobiotics Biodegradation and Metabolism	Xenobiotics Biodegradation and Metabolism	0.378	$< 10^{-4}$
Lipid Metabolism	Lipid Metabolism	0.349	$< 10^{-4}$
Nucleotide Metabolism	Nucleotide Metabolism	0.341	$< 10^{-4}$
Translation	Translation	0.333	$< 10^{-4}$
Metabolism of Other Amino Acids	Metabolism of Other Amino Acids	0.317	4.0×10^{-4}
Glycan Biosynthesis and Metabolism	Glycan Biosynthesis and Metabolism	0.292	$< 10^{-4}$
Kinase and Peptidase	Signal Transduction	0.214	$< 10^{-4}$
Metabolism of Other Amino Acids	Folding, Sorting and Degradation	0.213	$< 10^{-4}$
Biosynthesis of Polyketides and Terpenoids	Biosynthesis of Other Secondary Metabolites	0.174	1.0×10^{-4}
Xenobiotics Biodegradation and Metabolism	Folding, Sorting and Degradation	0.167	1.0×10^{-4}
