# Supplementary material for: Genetic Co-Occurrence Network across Sequenced Microbes
Source: PLoS Comput Biol. 2011 Dec 29;7(12):e1002340. doi: 10.1371/journal.pcbi.1002340 (PMC3248385; doi:10.1371/journal.pcbi.1002340)
Supplement: Table S5 — Pairs of correlog groups associated via significant anti-correlogy with Z>10 (see Text S1). For each index of correlog groups, the corresponding genes can be found in Table S3. (PDF) [file pcbi.1002340.s008.pdf]

Table S5. Pairs of correlog groups associated via significant anti-correlogy with $Z>10$ (see Text S1). For each index of correlog groups, the corresponding genes can be found in Table S3.

Correlog group	Correlog group	*Z* score	Correlog group	Correlog group	*Z* score	Correlog group	Correlog group	*Z* score	Correlog group	Correlog group	*Z* score	Correlog group	Correlog group	*Z* score
G0	G1425	13.66	G71	G830	23.43	G189	G1186	18.48	G299	G867	17.10	G617	G749	15.11
G0	G1702	13.07	G73	G891	11.21	G190	G691	10.33	G301	G636	15.05	G624	G636	11.67
G1	G223	15.49	G75	G876	22.00	G193	G442	16.04	G301	G672	10.55	G624	G1813	40.68
G1	G437	20.80	G80	G489	12.09	G194	G675	11.67	G302	G917	18.48	G638	G871	13.07
G2	G787	13.91	G81	G1379	18.54	G202	G286	10.64	G303	G2051	10.09	G641	G737	22.68
G3	G833	14.26	G84	G193	13.07	G202	G1582	19.32	G304	G459	15.98	G653	G1410	13.07
G6	G312	12.09	G84	G810	13.07	G204	G279	13.75	G305	G1114	26.26	G654	G2021	11.30
G6	G361	17.75	G85	G1230	12.09	G207	G1174	16.04	G312	G1195	14.33	G666	G1126	13.07
G7	G1328	37.13	G86	G644	45.50	G210	G604	24.26	G316	G1891	15.05	G669	G803	21.38
G8	G1187	13.07	G86	G861	10.09	G210	G965	16.80	G320	G542	18.48	G679	G1786	18.54
G9	G250	14.64	G87	G165	13.07	G211	G676	22.73	G327	G666	12.01	G684	G725	15.11
G10	G354	13.43	G87	G395	10.09	G211	G1180	18.54	G332	G342	13.07	G696	G740	18.54
G10	G1933	18.54	G89	G486	11.16	G216	G340	18.54	G339	G604	16.04	G696	G956	13.66
G11	G99	12.47	G92	G292	11.93	G216	G705	18.54	G339	G1499	14.33	G701	G867	13.07
G12	G414	18.43	G92	G1005	12.12	G217	G2021	20.27	G351	G1472	11.03	G708	G1350	32.17
G12	G1063	11.67	G93	G833	10.70	G221	G332	15.05	G354	G648	10.64	G711	G833	16.15
G12	G1512	18.54	G94	G115	24.82	G223	G935	18.89	G354	G718	21.38	G711	G1442	13.07
G14	G980	13.75	G94	G156	11.67	G224	G654	10.09	G357	G753	13.07	G711	G2017	32.08
G14	G1891	10.95	G94	G1050	23.43	G226	G1000	10.55	G357	G788	12.09	G725	G1133	15.05
G15	G183	11.59	G96	G426	19.78	G227	G444	10.09	G358	G1132	16.04	G741	G1714	39.36
G15	G858	10.12	G103	G760	31.25	G230	G976	10.70	G361	G457	36.43	G749	G789	13.59
G16	G282	13.59	G103	G1111	13.07	G235	G520	10.26	G364	G653	10.64	G752	G1658	23.43
G17	G18	10.33	G104	G737	22.73	G235	G669	11.21	G366	G1236	15.73	G773	G1198	37.13
G18	G96	11.12	G104	G1602	16.04	G235	G1668	11.39	G369	G603	13.07	G787	G1428	11.30
G19	G33	13.66	G106	G184	10.40	G235	G1785	17.10	G373	G896	11.90	G788	G858	10.64
G19	G50	14.08	G106	G269	15.05	G238	G240	14.64	G379	G911	16.04	G788	G882	22.73
G19	G279	11.30	G107	G196	10.05	G238	G1187	16.51	G381	G1390	14.33	G794	G1110	21.38
G21	G970	17.93	G108	G787	13.07	G239	G419	26.22	G388	G488	13.07	G806	G1110	11.67
G22	G704	17.75	G111	G1749	26.22	G239	G666	13.91	G393	G396	10.74	G845	G1789	16.04
G26	G31	12.55	G113	G199	15.05	G239	G875	12.12	G393	G1850	14.33	G858	G1674	12.09
G26	G1063	11.67	G113	G1809	21.46	G240	G312	14.33	G397	G871	14.84	G859	G1414	16.04
G26	G1907	32.17	G114	G364	10.64	G241	G273	30.54	G406	G425	10.09	G861	G882	11.30
G27	G196	15.73	G114	G480	16.51	G241	G696	14.26	G406	G750	18.54	G861	G1063	10.64
G28	G1819	15.73	G115	G266	12.99	G241	G865	13.43	G411	G787	10.64	G863	G1176	26.68
G29	G441	16.04	G116	G266	11.30	G244	G316	13.59	G411	G1525	13.07	G863	G1499	10.09
G30	G795	12.68	G116	G649	10.64	G244	G527	22.73	G413	G1453	23.43	G867	G1390	16.04
G33	G548	10.64	G129	G334	32.71	G246	G273	18.54	G437	G1171	18.54	G871	G1907	16.04
G33	G1819	17.15	G136	G1102	10.09	G246	G833	10.09	G441	G1999	13.07	G876	G891	27.39
G34	G649	11.49	G139	G489	11.69	G248	G395	13.07	G442	G1180	13.07	G882	G993	13.07
G35	G1509	40.68	G139	G1394	10.64	G250	G379	19.17	G444	G1141	11.67	G920	G1030	14.33
G36	G215	12.99	G140	G183	16.04	G251	G1604	13.75	G455	G608	34.37	G920	G2069	25.18
G36	G337	22.03	G143	G1123	14.33	G252	G711	13.07	G458	G1094	10.64	G951	G1094	10.64
G38	G251	12.01	G143	G1938	10.35	G254	G270	18.48	G460	G1497	18.54	G976	G1428	22.68
G38	G735	11.30	G145	G654	15.98	G254	G518	10.64	G470	G1163	10.64	G980	G1363	12.09
G38	G1414	11.30	G145	G1938	10.64	G254	G985	13.91	G470	G1509	11.67	G980	G1525	16.80
G40	G789	15.05	G146	G830	10.13	G254	G1171	10.64	G472	G685	32.17	G985	G1891	11.30
G40	G1714	12.09	G149	G1453	10.55	G255	G388	15.11	G476	G1313	15.49	G988	G1213	22.68
G41	G638	11.67	G150	G1036	14.45	G257	G753	10.64	G480	G641	17.75	G993	G1094	12.23
G42	G70	10.95	G152	G303	10.09	G257	G813	10.64	G486	G1893	13.07	G1005	G1987	13.07
G43	G369	12.47	G152	G1140	13.07	G259	G334	19.32	G487	G1076	16.04	G1006	G1499	10.09
G43	G845	10.09	G156	G342	26.22	G265	G398	11.30	G487	G1133	17.18	G1056	G1977	11.30
G45	G229	32.17	G159	G172	14.33	G266	G268	17.10	G488	G1350	22.73	G1063	G1081	10.95
G46	G172	10.64	G163	G740	13.07	G270	G805	16.04	G490	G548	11.21	G1081	G1394	10.64
G46	G357	10.09	G167	G344	12.09	G279	G616	17.04	G500	G1582	17.75	G1111	G1343	22.73
G46	G750	11.67	G167	G1602	16.04	G281	G297	13.75	G508	G612	45.48	G1123	G1418	22.73
G50	G136	10.70	G172	G701	11.67	G281	G859	12.12	G518	G523	18.54	G1129	G1213	12.73
G50	G794	12.23	G173	G589	15.73	G281	G1352	10.64	G521	G993	29.03	G1133	G1418	14.64
G52	G903	11.12	G173	G1425	20.27	G281	G1938	15.49	G521	G1180	18.54	G1166	G1410	16.24
G52	G941	10.55	G179	G1411	13.91	G282	G1891	10.64	G523	G701	15.11	G1192	G1242	11.30
G53	G1126	18.54	G179	G1439	10.05	G286	G672	10.26	G525	G1668	11.79	G1192	G1515	11.30
G59	G1166	20.80	G180	G254	10.64	G286	G1050	12.55	G542	G1423	16.04	G1192	G1602	13.59
G60	G219	26.22	G181	G207	22.73	G286	G1401	14.33	G548	G1056	10.09	G1278	G1668	11.67
G61	G381	11.67	G181	G985	16.04	G289	G320	27.78	G556	G1688	10.64	G1363	G1708	10.64
G61	G556	17.41	G182	G944	13.07	G289	G488	11.30	G589	G1649	12.55	G1439	G1486	18.54
G63	G226	15.05	G182	G1141	15.11	G292	G339	16.51	G603	G650	15.11	G1554	G1688	14.33
G65	G976	10.64	G182	G1236	10.64	G292	G697	13.07	G606	G763	11.03	G1604	G1786	21.38
G65	G1790	30.29	G183	G2051	11.30	G292	G965	15.28	G606	G1005	10.95			
G69	G1313	19.32	G188	G364	20.43	G295	G1089	40.68	G608	G1658	13.66			
G70	G344	14.64	G188	G414	11.64	G297	G863	11.30	G615	G833	18.11			
G70	G844	21.38	G189	G1166	12.23	G299	G616	11.30	G616	G684	12.60			
