# Supplementary material for: Genetic Co-Occurrence Network across Sequenced Microbes
Source: PLoS Comput Biol. 2011 Dec 29;7(12):e1002340. doi: 10.1371/journal.pcbi.1002340 (PMC3248385; doi:10.1371/journal.pcbi.1002340)
Supplement: Text S1 — Supporting methods and results. (PDF) [file pcbi.1002340.s009.pdf]

Supporting Information of "Genetic Co-Occurrence Network Across Sequenced Microbes"

Pan-Jun Kim and Nathan D. Price

Quantification of correlogy and anti-correlogy

We downloaded orthology data from the Kyoto Encyclopedia of Genes and Genomes (KEGG, November 2009) [1], and surveyed the presence or absence of each ortholog across different bacterial species. To avoid possible biases arising from redundant genomes, we considered only one subspecies from each species if it contains multiple subspecies.* Also, we excluded a few species because of their suspicious genome annotations (see Table S1 for the full list of the 588 bacterial species analyzed in this study). Because we are interested in functional interactions of genes reflected in their co-occurrence patterns across species, we take into account the genes (i.e., orthologs) not too lowly nor too highly prevalent across species; in the case of too lowly (highly) prevalent genes, there do not exist so many species with (without) the genes, making it hard to judge whether these few co-presences (co-absences) of the genes actually come from their functional interactions. In other words, without filtering, spurious correlations from non-functional origins may emerge, simply by vertical co-inheritance of genes or by chance. Specifically, if E_i denotes the number of species containing gene i and N denotes the total number of species, one can define $X_i = \min(E_i, N-E_i)$ for each gene i. The probability density of X_i approximately follows the power-law decay as long as $X_i \geq X_{th}$=80, and we chose the genes with $X_i \geq X_{th}$ to prevent spurious correlations that could occur at low X_i deviating from the power-law trend observed at large X_i. As such, the number of the resultant genes considered here was 2085 out of 5896 total. We also tried other lower bounds of X_i, ranging from 50 to 100, and found no qualitative difference from our main results.

The next step was to extract direct gene associations from the co-occurrence patterns. It should be noted that simple correlations calculated from the co-occurrence patterns can suffer from numerous indirect correlations between genes, caused by transitivity of direct correlations. Filtering out these indirect correlations has been of critical issues in the field of inferring transcriptional regulatory networks from microarray data [2,3]. Here, we applied the partial correlation method employed in graphical Gaussian models [3], of which superiority over many other methods was demonstrated in reverse engineering of transcriptional regulatory networks [2]. To implement this method, first we calculated the Pearson correlation r_{ij} for binary variables of presence and absence of genes i and j:

$$r_{ij} = \frac{C_{ij}N - E_iE_j}{\sqrt{E_iE_j(N-E_i)(N-E_j)}},$$

where C_{ij} is the number of species containing both genes i and j. Second, to reduce indirect correlations between genes i and j, we calculated the partial correlation w_{ij} using r_{ij}:

$$w_{ij} = -\frac{p_{ij}}{\sqrt{p_{ii} p_{jj}}},$$

where p_{ij} is the $(i, j)^{\text{th}}$ component of an inverse matrix of r_{ij}. However, in our case, the number of genes is much larger than the number of species, yielding an ill-conditioned problem for matrix r_{ij}. To overcome this problem, we applied the shrinkage estimation derived by Schäfer and Strimmer [3]. Specifically, Schäfer and Strimmer obtained a regularized estimator of r_{ij} combining analytic determination of shrinkage intensity from the Ledoit-Wolf theorem [4]. The following is the resultant estimator r^*_{ij} that simply substitutes for r_{ij} in the above calculation of w_{ij}:

$$r^*_{ij} = \delta_{ij} + r_{ij}(1-\delta_{ij})\min[1, \max(0, 1-\lambda)],$$

where δ_{ij} is the Kronecker delta symbol and λ is given by

$$\lambda = \frac{\sum_{i \neq j} \left\langle \left[\left(x_{ki} - \langle x_{ki} \rangle_k\right)\left(x_{kj} - \langle x_{kj} \rangle_k\right) \Big/ \left(\frac{N-1}{N} \sigma_i \sigma_j \right) - r_{ij} \right]^2 \right\rangle_k}{(N-1) \times \sum_{i \neq j} r_{ij}^2}.$$

Here $x_{ki} = 1$ if gene i is present in species k, otherwise, $x_{ki} = 0$, $\langle\cdots\rangle_k$ denotes the average over species k's, and σ_i is the standard deviation of x_{ki} over species k's.

We expect that the calculation of w_{ij} will also alleviate vertical co-inheritance effects, as indirect correlations from redundant genetic backgrounds can be reduced so. As a result, the obtained w_{ij} indicates correlogy ($w_{ij} > 0$) or anti-correlogy ($w_{ij} < 0$) between genes i and j, and $|w_{ij}|$ quantifies the magnitude of the correlogy and anti-correlogy. The significance range of w_{ij} was assessed by dissociating gene relationships with randomly-permuted presences of each gene across species: $P < 0.001$ as long as $|w_{ij}| > 0.0008$. For comparative analysis, the mutual information I_{ij} of genes i and j between $\{x_{ki}\}$ and $\{x_{kj}\}$ across species k's [5] was also calculated.

Significance analysis of correlation between w_{ij} (or r_{ij}, I_{ij}) and protein interaction

We calculated the average of w_{ij} (w^{ppi}) from the pairs of physically-binding proteins in *E. coli* [6], and obtained its P value by generating the distribution of average w_{ij} (w^{null}) from the same number of, but arbitrarily-mated pairs of the proteins as distant as the given shortest path length in the protein interaction network. The central limit theorem ensured that this null distribution converged well to the Gaussian distribution, providing the P value for how frequently w^{null} exceeds w^{ppi}. The smaller the P value, the more significantly large w_{ij} physically-binding proteins tend to have. Similar analyses were also performed for r_{ij} and I_{ij}.

Two distinct regimes of protein interactions

From w_{ij} ~ 0.045, the probability density of w_{ij} for interacting protein pairs in *E. coli* starts to record higher values than that for arbitrary pairs of proteins (Figure 1A). We hypothesize that such distinct w_{ij}'s in probability densities might represent distinct regimes enriched with obligatory or non-obligatory protein interactions. To investigate this possibility, we collected the operon data based on publicly available information [7], and found that the fraction belonging to the same operons among the gene pairs of given w_{ij} increases steeply at w_{ij} ~ 0.045 for both interacting and non-interacting protein pairs, but this increase appears more profound for interacting protein pairs (Figure S1, left). One might raise the possibility that the distinct regimes shown in Figure 1A can merely be due to the levels of operon pairs rather than due to the degree of functional coherence in protein interactions. However, even if we exclude the pairs belonging to the same operons, the trends similar to those in Figure 1A still persist although rather weakened (Figure S1, center; $P = 4.8\times10^{-7}$). The further analysis of these non-operonic pairs with the Affymetrix *E. coli* oligonucleotide array data [8] reveals that the gene pairs with w_{ij} > 0.045 have higher Pearson correlation coefficient ρ_{ij}'s of transcript profiles than the others, as especially manifested for the genes encoding interacting protein pairs (Figure S1, right); for interacting protein pairs, the average ρ_{ij} of w_{ij} > 0.045 is 4.08 times larger than that of w_{ij} < 0.045, while for arbitrary protein pairs, 2.05 times larger. Taken together, our results indicate the overall enrichment of functionally-obligatory interacting proteins at w_{ij} > 0.045.

Functional enrichment of correlogy and anti-correlogy

For a given pair of functional categories $c1$ and $c2$, we can quantify how correlogously genes in $c1$ and $c2$ are associated by:

$$\Omega^{p}_{c1,c2} = \frac{\sum_{i,j}^{i\neq j} \frac{w_{ij}}{b_i b_j} a_{i,c1} \delta_{j,g^{p}_{i,c2}}}{\sum_{i,j}^{i\neq j} \frac{a_{i,c1}\delta_{j,g^{p}_{i,c2}}}{b_i b_j}},$$

where i and j are indices of genes, $a_{i,c}$ is 1 if gene i belongs to functional category c, otherwise 0, $\delta_{i,j}$ is the Kronecker delta symbol, $g^{p}_{i,c}$ is the index of gene satisfying $a_{g^{p}_{i,c},c} = 1$ and $w_{i,g^{p}_{i,c}} = \max(w_{ij} a_{j,c})$ for given i and c, $b_i = \sum_c a_{i,c}$, and the summation over the pairs of i and j does not double-count each pair of i and j. $\Omega^{p}_{c1,c2}$ is a weighted average of w_{ij} > 0 according to how exclusively genes i and j belong to $c1$ and $c2$. Likewise, we can define $\Omega^{n}_{c1,c2}$ to quantify how anti-correlogously genes in $c1$ and $c2$ are associated:

$$\Omega^{n}_{c1,c2} = \frac{\sum_{i,j}^{i\neq j} \frac{|w_{ij}|}{b_i b_j} a_{i,c1} \delta_{j,g^{n}_{i,c2}}}{\sum_{i,j}^{i\neq j} \frac{a_{i,c1}\delta_{j,g^{n}_{i,c2}}}{b_i b_j}},$$

where $g^n_{i,c}$ is the index of gene satisfying $a_{g^n_{i,c},c} = 1$ and $w_{i,g^n_{i,c}} = \min(w_{ij} a_{j,c})$ for given i and c. $\Omega^n_{c1,c2}$ is a weighted average of $|w_{ij}|$ ($w_{ij} < 0$) according to how exclusively genes i and j belong to $c1$ and $c2$. Note that for both $\Omega^p_{c1,c2}$ and $\Omega^n_{c1,c2}$, the cases with $c1 = c2$ as well as with $c1 \neq c2$ are all allowed. Here, we generally followed the functional classification of orthologs taken by KEGG, but slightly modified it to better consider bacterium-specific physiology.

Figure S2 clearly shows high $\Omega^p_{c1,c2}$ and $\Omega^n_{c1,c2}$ when $c1 = c2$, indicating the enrichment of correlogy and anti-correlogy between genes of similar biological functions. For example, genes *rrf*, *rrs*, and *rrl* in common functional category *Translation* encode 5S rRNA, 16S rRNA, and 23S rRNA, respectively, and are very strongly correlogous to each other; $w_{ij} = 0.343$ for *rrf* and *rrs* (the 12th largest $w_{ij} > 0$ among all gene pairs), $w_{ij} = 0.307$ for *rrf* and *rrl* (the 22nd largest $w_{ij} > 0$), and $w_{ij} = 0.3975$ for *rrs* and *rrl* (the 3rd largest $w_{ij} > 0$). In the same functional category, on the other hand, highly anti-correlogous are *lysK* and *lysS* encoding lysyl-tRNA synthetase ($w_{ij} = -0.124$, the 8th smallest $w_{ij} < 0$ among all gene pairs) as well as genes encoding glycyl-tRNA synthetase [$w_{ij} = -0.123$ for *glyQ* and *glyS1* (the 9th smallest $w_{ij} < 0$), and $w_{ij} = -0.120$ for *glyS* and *glyS1* (the 11th smallest $w_{ij} < 0$)].

Characterization of S_i^p and S_i^n

In order to quantify how tightly each gene i is correlogously associated to other genes, we defined $S_i^p = \sum_j^{w_{ij}>0} w_{ij}$, where the summation was taken over all other gene j's satisfying $w_{ij} > 0$. In a similar way, we defined $S_i^n = \sum_j^{w_{ij}<0} |w_{ij}|$ for anti-correlogous couplings around gene i.

Correlation between S_i^p and S_i^n

With respect to the organizational property of correlogy versus anti-correlogy, S_i^p and S_i^n of each gene i are remarkably positively correlated ($r = 0.99$; Figure S3, left). This result does not seem to be caused by a methodological artifact, as S_i^p and S_i^n from surrogate data with randomly shuffled ortholog profiles show clear negative correlations [$r = -0.48 \pm 0.03$ (average ± s.d.)]. In surrogate data, S_i^p and S_i^n of gene i are roughly proportional to the numbers of other gene j's with $w_{ij} > 0$ and $w_{ij} < 0$, respectively, while the total number of gene j's is always finite. This zero-sum relation between S_i^p and S_i^n in surrogate data leads to such negative correlations. In other words, the positive correlation between S_i^p and S_i^n out of real ortholog profiles can only be accounted for by nontrivial fine-level structures behind w_{ij}'s.

To address this issue, we measured the Shannon disparities of $w_{ij} > 0$ and $w_{ij} < 0$ for each gene i (D_i^p and D_i^n, respectively) [9]:

$$D_i^p = \prod_j^{w_{ij}>0} \tilde{w}_{ij}^{p\,-\tilde{w}_{ij}^p}, \quad D_i^n = \prod_j^{w_{ij}<0} \tilde{w}_{ij}^{n\,-\tilde{w}_{ij}^n},$$

where $\tilde{w}_{ij}^{p} = w_{ij} / \sum_{j}^{w_{ij}>0} w_{ij}$ and $\tilde{w}_{ij}^{n} = |w_{ij}| / \sum_{j}^{w_{ij}<0} |w_{ij}|$. One can notice that D_i^p and D_i^n are the exponentials of the Shannon entropies of $\tilde{w}_{ij}^{p}$ and $\tilde{w}_{ij}^{n}$. The more homogeneously distributed $w_{ij} > 0$ (< 0) around gene i, the larger D_i^p (D_i^n). D_i^p and D_i^n quantify how many genes are 'effectively' associated to gene i correlogously and anti-correlogously, respectively. The coefficient of variation (CV) of D_i^p over gene i's was 0.058 and that of D_i^n was 0.025, indicating that D_i^n is more constant over gene i's than D_i^p. In other words, the effective number of anti-correlogous associations around a gene tends to be constant relative to that of correlogous associations, and S_i^n can be better proportional to individual $|w_{ij}|$'s ($w_{ij} < 0$) around gene i than S_i^p can be to individual w_{ij}'s ($w_{ij} > 0$) around that gene. Indeed, it turns out that if the largest $|w_{ij}|$ ($w_{ij} < 0$) around gene i is w_i^{nmax} and the largest w_{ij} ($w_{ij} > 0$) around that gene is w_i^{pmax}, $r = 0.59$ for w_i^{nmax} and S_i^n while $r = -0.10$ for w_i^{pmax} and S_i^p.

Because S_i^n is substantially determined by individual w_{ij}'s although S_i^p is not, we suggest that the positive correlation between S_i^p and S_i^n originates from the correlation between S_i^p and individual w_{ij}'s ($w_{ij} < 0$), as shown by $r = 0.56$ for S_i^p and w_i^{nmax} (Figure S3, right; $Z = 28.71$ from surrogate data with randomly-shuffled ortholog profiles). Therefore, genes with higher S_i^p are also likely to encounter more severely anti-correlogous genes. Why are genes in strong correlogous associations likely to encounter more anti-correlogous genes? We offer the possibility that if a certain gene *A* in a cell collaborates with other genes more tightly, harboring its anti-correlog *B* can antagonistically interfere with the entire functions performed by these collaborating genes, thereby making *A* and *B* more anti-correlogous to each other.

Significance analysis of correlation between S_i^p and phylum-level dispersion

Let n^{phyla} be the number of different phyla where genes are present. For genes with $n^{phyla} < 7$ (Figure 2B), we obtained the slope of S_i^p against n^{phyla} by linear regression, and normalized it by multiplying $\langle n^{phyla} \rangle / \langle S_i^p \rangle$. From surrogate data with randomly-permuted gene presences across species, we also generated an ensemble of such normalized slopes for $n^{phyla} < 7$, and calculated the Z score of the actual value. The larger the Z score, the more significantly large the slope of S_i^p against n^{phyla}.

Characterization of the maximum relatedness subnetwork (MRS)

For any given weighted network, one can simplify its structure by constructing the MRS composed only of highly weighted edges in the network [9]. Specifically, in the MRS of this study, each gene i points to only two genes j and j' by different categories of edges that represent the most correlogous ($\max_j w_{ij} > 0$) and anti-correlogous ($\min_{j'} w_{ij'} < 0$) genes to gene i, respectively. Here, all genes in the MRS turned out to be decomposed into 483 different small subgroups, of which each includes correlogously associated genes yet not linked correlogously to any genes in the other subgroups. These subgroups in the MRS were termed correlog groups.

Functional coherence of correlog groups in the MRS

For given correlog group g in the MRS and given functional category c of genes, we can calculate $f_c^g = \tilde{N}_c^g/\tilde{N}^g$, where $\tilde{N}_c^g$ is the number of genes affiliated to both correlog group g and functional category c, and $\tilde{N}^g$ is the total number of genes affiliated at least to one functional category in correlog group g. Therefore, f_c^g represents the uniformity of gene functions in a correlog group. Majority (57.1%) of correlog groups with $\tilde{N}^g > 1$ were shown to have at least one functional category c satisfying $f_c^g = 1$ in each g. To calculate the corresponding Z score, we generated an ensemble of correlog groups with $\tilde{N}^g > 1$ by randomly exchanging genes of the same number of the affiliated functional categories. The larger the Z score, the more significantly uniform gene functions each correlog group tends to have.

For a given pair of functional categories $c1$ and $c2$, we can also define their overlapping ratio (Figure 4A and Table S4) as:

$$Y_{c1,c2} = \frac{\sum_{g}^{\tilde{N}^g>1} H\left(\sum_{i,j}^{i\neq j} a_{i,c1} a_{j,c2} d_{i,g} d_{j,g}\right)}{\sum_{g}^{\tilde{N}^g>1} H\left[\sum_{i}\left(a_{i,c1} + a_{i,c2}\right) d_{i,g}\right]},$$

where i and j are indices of genes, $a_{i,c}$ is 1 if gene i belongs to functional category c, otherwise 0, $d_{i,g}$ is 1 if gene i belongs to correlog group g, otherwise 0, and $H(x)$ is 1 if $x > 0$, otherwise 0. $Y_{c1,c2}$ quantifies how likely genes in $c1$ and $c2$ belong to the same correlog groups ($0 \leq Y_{c1,c2} \leq 1$). The corresponding P value was obtained by generating the null distribution in the same way as in the case of f_c^g above. As mentioned before, we here employed the modified version of the KEGG functional categories suited to bacterium-specific physiology.

Anti-correlogous associations between different correlog groups in the MRS

Correlog groups in the MRS are seamlessly bridged by anti-correlogy links, allowing us to identify which correlog groups are significantly associated anti-correlogously with each other. For a given pair of correlog groups $g1$ and $g2$, we can count the number of anti-correlogy links ($L_{g1,g2}$) bridging $g1$ and $g2$. We compared $L_{g1,g2}$ with the values expected from the configuration model [10,11] to have the given numbers of incoming and outgoing anti-correlogy links for $g1$ and $g2$, and calculated the Z score of $L_{g1,g2}$:

$$Z = \frac{L_{g1,g2} - \dfrac{k_{g1}^{in} k_{g2}^{out} + k_{g1}^{out} k_{g2}^{in}}{m}}{\sqrt{\dfrac{k_{g1}^{in} k_{g2}^{out}}{m}\left(1 - \dfrac{k_{g1}^{in} k_{g2}^{out}}{m^2}\right) + \dfrac{k_{g1}^{out} k_{g2}^{in}}{m}\left(1 - \dfrac{k_{g1}^{out} k_{g2}^{in}}{m^2}\right)}},$$

where k_g^{in} is the number of anti-correlogy links of correlog group g incoming from the other correlog groups, k_g^{out} is the number of those outgoing to the other correlog groups, and m is the total number of anti-correlogy links bridging different correlog groups. The larger the Z score, the more significantly two correlog groups are associated anti-correlogously (Table S5).

Effectiveness of correlog groups for different domains of life and environmental samples

To map orthologs in MRS to genes in archaea and eukaryotes, we considered 60 different archaeal species and 106 different eukaryotic species in KEGG. For this, we considered only one subspecies from each species to avoid biased analysis for any particular species.* For the mapping for environmental samples, we considered the data from twelve various environmental sources, available at the integrated microbial genomes (IMG) system [12], and selected only one sample from each source to prevent biasing the analysis for any particular source.† The twelve environmental samples were from human gut microbial communities [13], methane-oxidizing archaea from deep-sea sediments [14], hypersaline microbial mats [15], marine planktonic communities [16], acid mine drainages [17], mouse gut microbial communities [18], termite gut microbial communities [19], deep-sea whale fall carcasses [20], uranium contaminated groundwater [21], indoor atmosphere [22], agricultural soil [20], and lake sediments [23].

For each species or environmental sample, we counted the number (n) of correlog groups harboring the genes mapped to the MRS. We also obtained the mean (η) and the standard deviation (σ) of such numbers of correlog groups when the same number of genes are randomly mapped to the MRS (Figure 4C-4E):

$$\eta = \bar{n} - \sum_g \left(1 - \frac{N}{\sum_g N_g}\right)^{N_g}, \quad \sigma = \sqrt{\sum_g \left[\left(1 - \frac{N}{\sum_g N_g}\right)^{N_g} - \left(1 - \frac{N}{\sum_g N_g}\right)^{2N_g}\right]},$$

where N is the number of genes mapped to the MRS, g is the index of each correlog group, N_g is the total number of genes in correlog group g, and $\bar{n}$ is the total number of correlog groups in the MRS. Accordingly, we can calculate the Z score of n [$Z = (n-\eta)/\sigma$]. The smaller the Z score below zero, the more significantly clustered the genes around correlog groups.

Footnotes

*The earliest sequenced subspecies from each species was chosen for analysis based on the assumption that genome annotations of earlier sequenced subspecies might be more updated. Selections of other subspecies also did not affect the main results presented here.

†From each source, selected was for analysis the sample containing the largest number of genes mapped to the MRS, based on the assumption that a richer genetic content in the same source might be of better quality. We also tried other selections of samples, but did not find much difference from the results presented here.

References

1. Kanehisa M, Goto S (2000) KEGG: Kyoto Encyclopedia of Genes and Genomes. Nucleic Acids Res 28:27−30.
2. Soranzo N, Bianconi G, Altafini C (2007) Comparing association network algorithms for reverse engineering of large-scale gene regulatory networks: Synthetic versus real data. Bioinformatics 23:1640−1647.
3. Schäfer J, Strimmer K (2005) A shrinkage approach to large-scale covariance matrix estimation and implications for functional genomics. Stat Appl Genet Mol Biol 4:32.
4. Ledoit O, Wolf M (2003) Improved estimation of the covariance matrix of stock returns with an application to portfolio selection. J Empir Finance 10: 603−621.
5. Huynen M, Snel B, Lathe W, III, Bork P (2000) Predicting protein function by genomic context: Quantitative evaluation and qualitative inferences. Genome Res 10:1204−1210.
6. Hu P, Janga SC, Babu M, Díaz-Mejía JJ, Butland G, et al. (2009) Global functional atlas of *Escherichia coli* encompassing previously uncharacterized proteins. PLoS Biol **7**:e1000096.
7. Gama-Castro S, Jiménez-Jacinto V, Peralta-Gil M, Santos-Zavaleta A, Peñaloza-Spinola MI, et al. (2008) RegulonDB (version 6.0): Gene regulation model of *Escherichia coli* K-12 beyond transcription, active (experimental) annotated promoters and Textpresso navigation. Nucleic Acids Res 36:D120−D124.
8. Allen TE, Herrgård MJ, Liu M, Qiu Y, Glasner JD, et al. (2003) Genome-scale analysis of the uses of the *Escherichia coli* genome: Model-driven analysis of heterogeneous data sets. J Bacteriol 185:6392−6399.
9. Lee SH, Kim P-J, Ahn Y-Y, Jeong H (2010) Googling social interactions: Web search engine based social network construction. PLoS ONE 5:e11233.
10. Newman MEJ, Strogatz SH, Watts DJ (2001) Random graphs with arbitrary degree distributions and their applications. Phys Rev E 64:026118.
11. Molloy M, Reed B (1995) A critical point for random graphs with a given degree sequence. Random Struct Algorithms 6:161−179.

12. Markowitz VM, Chen I-MA, Palaniappan K, Chu K, Szeto E, et al. (2010) The integrated microbial genomes system: An expanding comparative analysis resource. Nucleic Acids Res 38:D382−D390.
13. Gill SR, Pop M, DeBoy RT, Eckburg PB, Turnbaugh PJ, et al. (2006) Metagenomic analysis of the human distal gut microbiome. Science 312:1355−1359.
14. Hallam SJ, Putnam N, Preston CM, Detter JC, Rokhsar D, et al. (2004) Reverse methanogenesis: Testing the hypothesis with environmental genomics. Science 305:1457−1462.
15. Kunin V, Raes J, Harris JK, Spear JR, Walker JJ, et al. (2008) Millimeter-scale genetic gradients and community-level molecular convergence in a hypersaline microbial mat. Mol Syst Biol 4:198.
16. DeLong EF, Preston CM, Mincer T, Rich V, Hallam SJ, et al. (2006) Community genomics among stratified microbial assemblages in the ocean's interior. Science 311:496−503.
17. Tyson GW, Chapman J, Hugenholtz P, Allen EE, Ram RJ, et al. (2004) Community structure and metabolism through reconstruction of microbial genomes from the environment. Nature 428:37−43.
18. Turnbaugh PJ, Ley RE, Mahowald MA, Magrini V, Mardis ER, et al. (2006) An obesity-associated gut microbiome with increased capacity for energy harvest. Nature 444:1027−1031.
19. Warnecke F, Luginbühl P, Ivanova N, Ghassemian M, Richardson TH, et al. (2007) Metagenomic and functional analysis of hindgut microbiota of a wood-feeding higher termite. Nature 450:560−565.
20. Tringe SG, von Mering C, Kobayashi A, Salamov AA, Chen K, et al. (2005) Comparative metagenomics of microbial communities. Science 308:554−557.
21. Hemme CL, Deng Y, Gentry TJ, Fields MW, Wu L, et al. (2010) Metagenomic insights into evolution of a heavy metal-contaminated groundwater microbial community. ISME J 4:660−672.
22. Tringe SG, Zhang T, Liu X, Yu Y, Lee WH, et al. (2008) The airborne metagenome in an indoor urban environment. PLoS ONE 3:e1862.
23. Kalyuzhnaya MG, Lapidus A, Ivanova N, Copeland AC, McHardy AC, et al. (2008) High-resolution metagenomics targets specific functional types in complex microbial communities. Nat Biotechnol 26:1029−1034.
